# Notch signaling suppresses neuroendocrine differentiation and alters the immune microenvironment in advanced prostate cancer

**DOI:** 10.1172/JCI175217

**Published:** 2024-07-18

**Authors:** Sheng-Yu Ku, Yanqing Wang, Maria Mica Garcia, Yasutaka Yamada, Kei Mizuno, Mark D. Long, Spencer Rosario, Meenalakshmi Chinnam, Majd Al Assaad, Loredana Puca, Min Jin Kim, Martin K. Bakht, Varadha Balaji Venkadakrishnan, Brian D. Robinson, Andrés M. Acosta, Kristine M. Wadosky, Juan Miguel Mosquera, David W. Goodrich, Himisha Beltran

**Affiliations:** 1 Department of Medical Oncology, Dana-Farber Cancer Institute, Boston, Massachusetts, USA.; 2 Department of Pharmacology and Therapeutics and; 3 Department of Biostatistics and Bioinformatics, Roswell Park Comprehensive Cancer Center, Buffalo, New York, USA.; 4 Department of Pathology and Laboratory Medicine and; 5 Department of Medicine, Weill Cornell Medicine, New York, New York, USA.; 6 Department of Pathology, Brigham and Women’s Hospital, Boston, Massachusetts, USA.; 7 Department of Urology, Roswell Park Comprehensive Cancer Center, Buffalo, New York, USA.

**Keywords:** Oncology, Prostate cancer

## Abstract

Notch signaling can have either an oncogenic or tumor-suppressive function in cancer depending on the cancer type and cellular context. While Notch can be oncogenic in early prostate cancer, we identified significant downregulation of the Notch pathway during prostate cancer progression from adenocarcinoma to neuroendocrine (NE) prostate cancer, where it functions as a tumor suppressor. Activation of Notch in NE and *Rb1*/*Trp53*-deficient prostate cancer models led to phenotypic conversion toward a more indolent, non-NE state with glandular features and expression of luminal lineage markers. This was accompanied by upregulation of MHC and type I IFN and immune cell infiltration. Overall, these data support Notch signaling as a suppressor of NE differentiation in advanced prostate cancer and provide insights into how Notch signaling influences lineage plasticity and the tumor microenvironment (TME).

## Abstract 2

Notch signaling regulates neuroendocrine differentiation in prostate cancer, highlighting a mechanism by which tumor cells exploit normal developmental processes to develop alternative lineages.

## Graphical abstract

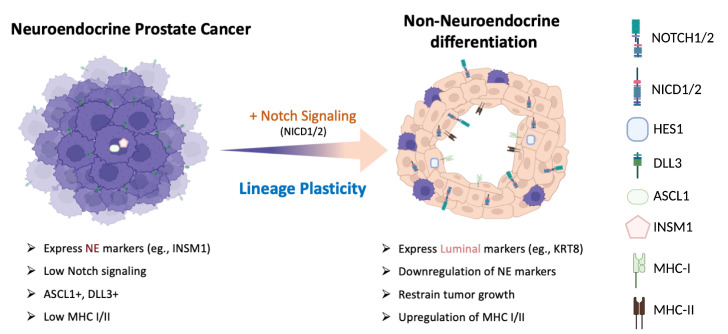

## Introduction

Phenotypic plasticity is a hallmark of cancer (1–3). In advanced prostate cancer, up to 15%–20% of patients develop resistance to androgen receptor–directed (AR-directed) therapies through conversion from an androgen-driven adenocarcinoma to an alternative lineage state such as neuroendocrine prostate cancer (NEPC) (4–6). NEPC does not depend on AR signaling, in part due to loss of AR expression. Prostate cancer lineage plasticity is dynamic, with a spectrum of phenotypes observed during the transition toward NEPC. The development of NEPC is facilitated by genomic loss of retinoblastoma 1 (*RB1*) and tumor protein P53 (*TP53*) (7, 8), downregulation of RE1 Silencing Transcription Factor (REST) (9), and dysregulation of epigenetic regulators and key transcription factors (8, 10–16). NEPC is increasingly recognized in the clinic, but therapeutic options are limited, and the prognosis is poor (4, 17). The underlying mechanisms that drive lineage plasticity and NEPC development are incompletely understood.

In addition to intrinsic cellular drivers, tumor plasticity is also modulated by the tumor microenvironment (TME). Crosstalk between cancer and its tumoral niche facilitates tumor growth and metastasis (18). Inflammation, hypoxia, and an immunosuppressive TME can modulate cellular plasticity to support tumor growth and therapy resistance (18). How prostate cancer lineage plasticity and the TME reciprocally influence one another remains largely unknown.

We previously identified delta-like ligand 3 (DLL3) as a cell-surface target expressed in the majority (>75%) of NEPC cases, a subset of castration-resistant adenocarcinomas (12%), and less than 1% of primary localized prostate adenocarcinomas (PCas) (19). DLL3 is an inhibitory ligand of the Notch signaling pathway that is transcriptionally activated by Achaete-Scute family BHLH transcription factor 1 (ASCL1) (20). The highly conserved Notch signaling pathway is central for normal development, including neuronal lineage commitment. NOTCH has also been implicated in cancer, with divergent roles across different tumor types, and can either promote or suppress tumor growth and affect tumor cell fate choices. There are 4 Notch receptors (*NOTCH1–4*), each with an extracellular region, transmembrane, and intracellular domain (NICD), but only *NOTCH1* and *NOTCH2* have a transcriptional activating domain that is required to activate downstream gene expression (21, 22). The function of NOTCH is dependent on cell-to-cell interactions. Binding of ligand from an adjacent cell to the extracellular domain generates soluble NICD that is transferred to the nucleus. During normal neuronal lineage commitment, Notch signaling is downregulated via lateral inhibition of neural stem cells to initiate neurogenesis and neuronal maturation (23, 24). ASCL1, which is suppressed by Notch signaling, is a driver of a subset of neuroendocrine (NE) carcinomas including small cell lung cancer (25) and NEPC (16). Prior work has demonstrated that the YAP/Notch signaling axis induces rapid degradation of ASCL1 (26) and drives expression of the REST transcription factor to suppress NE lineage commitment (27). Overall, these observations point to a potential functional role of Notch signaling in NEPC.

On the other hand, Notch signaling is considered oncogenic in early prostate cancer (28–30). Activation of Notch signaling contributes to AR-driven therapeutic resistance (31–34). Therefore, the role of Notch signaling may be context dependent in prostate cancer, with an oncogenic role in AR-driven disease and a tumor-suppressive role later in the context of lineage plasticity and conversion to NEPC.

Here, we leveraged both human and mouse models of prostate cancer to resolve these multifaceted and potentially divergent roles of Notch signaling in prostate cancer. We found that Notch signaling altered prostate cancer multilineage plasticity and the immune microenvironment in NEPC, suppressed NEPC development, and had disparate effects on prostate cancer progression depending on the cancer’s genetic background.

## Results

### Notch signaling is downregulated in NEPC.

**Figure 1:**
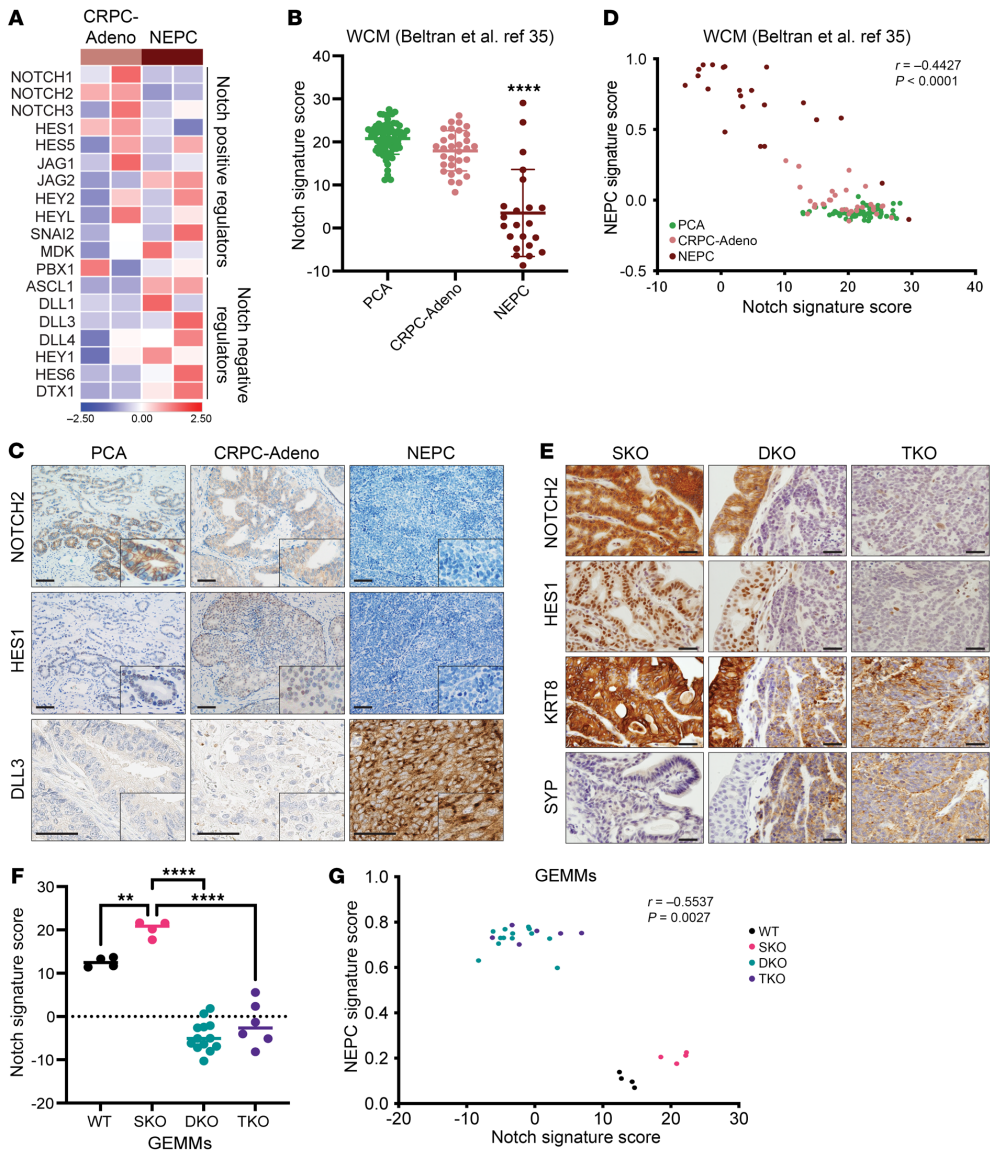
Notch signaling activity during prostate cancer progression. (**A**) Expression of the 19-gene Notch signaling mRNA score. Representative cases of CRPC-Adeno PCa (*n* = 2) and NEPC (*n* = 2) are shown. Expression levels were *Z* transformed. (**B**) The Notch score was significantly lower in NEPC (*n* = 22) than in hormone-naive PCa (*n* = 68) or CRPC-Adeno (*n* = 31) in the Beltran data set (35). ****P < 0.0001, by 1-way ANOVA. (**C**) Clinical specimens of PCa, CRPC-Adeno PCa, and NEPC were stained for protein expression of NOTCH2, HES1, and DLL3. Scale bars: 200 μm. Original magnification, ×10 (NOTCH2, HES1); ×40 (DLL3) (insets). (**D**) Spearman’s correlation analysis of the Notch signaling and NEPC scores showed a significant negative correlation in the Beltran data set (*r* = –0.4427, ****P < 0.0001) (35). (**E**) SKO, DKO, agnd TKO GEMM tumors were stained for NOTCH2, HES1, KRT8, and SYP. Scale bars: 50 μm. (**F**) Notch signaling score in the indicated GEMMs. **P < 0.01 and ****P < 0.0001, by 1-way ANOVA (SKO vs. DKO, SKO vs. TKO). (**G**) The Notch signaling and NEPC scores were negatively correlated in GEMMs (Spearman’s *r* = –0.5537, *P* = 0.0027).

To investigate the role of the Notch pathway in prostate cancer progression, we mined transcriptome data from 3 metastatic castration-resistant prostate cancer patient cohorts including ours (35), the International Stand Up to Cancer–Prostate Cancer Foundation (SU2C/PCF) Dream Team (36), and the Fred Hutchinson Cancer Research Center (FHCRC) (37) (Supplemental Figure 1, A and B; supplemental material available online with this article; https://doi.org/10.1172/JCI175217DS1). Notch signaling status was measured by a 19-gene Notch signaling score (Figure 1A and Supplemental Figure 1C). In all 3 data sets, the Notch signaling score was significantly lower in NEPC compared with localized and castration-resistant (CRPC-Adeno) prostate adenocarcinoma (PCa) (Figure 1B and Supplemental Figure 1, D and E). We observed downregulation of Notch signaling during transdifferentiation from adenocarcinoma to NEPC in the LTL331 patient-derived xenograft (PDX) model (38) (Supplemental Figure 1E). We also evaluated the Notch signaling score in potential intermediary phenotypes, including amphicrine tumors expressing both the AR and NE markers (AR^+^NE^+^) and double negative tumors that were negative for both (AR^–^NE^–^), and found that these subtypes had Notch scores similar to those of AR^+^NE^–^ CRPC-Adeno NEPC tumors and higher scores than AR^–^NE^+^ NEPC tumors (Supplemental Figure 1F). This was also seen in the LuCaP series of PDXs (39) (Supplemental Figure 1G). Low levels of NOTCH2 and Hes family BHLH transcription factor 1 (HES1) along with higher levels of DLL3 were also confirmed at the protein level in NEPC (Figure 1C and Supplemental Figure 2). Notch score negatively correlated with a reported 70-gene NEPC signature score (35) (Figure 1D and Supplemental Figure 3, A–C) and positively correlated with the AR signaling score (Supplemental Figure 3, D–G). In addition, there was an inverse correlation between *ASCL1* expression and the Notch score in all data sets (Supplemental Figure 3, H–K).

We assessed Notch signaling in 3 genetically engineered mouse models (GEMMs) that utilize a *Pbsn*-derived promoter to drive Cre expression specifically in prostate epithelial cells (PBCre4) (40). PBCre4 *Pten^fl/fl^* mice (single-KO [SKO]) develop low-grade PCa that does not progress to NEPC (7, 41); PBCre4 *Pten^fl/fl^* *Rb1^fl/fl^* (double-KO [DKO]) mice develop adenocarcinoma that slowly progress to high-grade carcinoma with NE features. PBCre4 *Pten^fl/fl^* *Rb1^fl/fl^* *Trp53^fl/fl^* (triple-KO [TKO]) mice rapidly progress to NEPC that is resistant to surgical castration (7). RNA-Seq of end-stage tumors revealed that SKO tumors expressed higher levels of Notch receptors, activating ligands, and Notch target genes compared with DKO and TKO tumors (Supplemental Figure 4A). IHC confirmed reduced protein levels of both NOTCH2 and HES1 in DKO and TKO tumors compared with SKO. Reduced Notch signaling correlated with increased protein expression levels of the NE marker synaptophysin (SYP) and inversely with the luminal marker cytokeratin 8 (KRT8) (Figure 1E). We noted that SKO mice had a significantly higher Notch signaling score than did WT mice, consistent with a previous finding that Notch signaling is oncogenic in early PCa development (28). Similar to the patient data, the Notch signaling score was significantly reduced in DKO and TKO tumors compared with SKO tumors (Figure 1F) and negatively correlated with the NEPC score (Figure 1G). The correlation between Notch and AR signaling was not statistically significant (Supplemental Figure 4B).

We performed single-cell transcriptome analysis (scRNA-Seq) of prostate tissues from 3 SKO mice aged 12–58 weeks and 5 TKO mice aged 8–16 weeks. These age ranges spanned early-to-late disease in the respective GEMMs. Neoplastic cells were marked by EGFP expression in both GEMMs using a Cre recombinase reporter (42). Similar to our prior report (43), EGFP^+^ cells differentiated multiple prostate cancer lineages including NEPC, luminal-like, basal-like, and a rarer tuft cell–like variant (Supplemental Figure 4C). NE and tuft-cell like variants were unique to TKO mice, while the luminal-like and basal-like state were shared by both SKO and TKO mice. *Notch1*, *Notch2*, *Hes1*, and jagged canonical Notch ligand 1 (*Jag1*) were expressed in luminal- and basal-like clusters, but not in the NEPC cluster; conversely, *Ascl1*, *Insm1* (insulinoma-associated protein 1), *Foxa2* (Forkhead box A2), and *Dll3* were expressed in the NEPC cluster only (Supplemental Figure 4D). Moreover, luminal- and basal-like cells showed a high Notch score and a low NEPC signature score, while the NEPC cluster displayed the opposite trend (Supplemental Figure 4, E and F). Thus, NE differentiation and Notch signaling activity were mutually exclusive in nearly all prostate cancer cells examined. Consistent with this, protein expression of the NE markers SYP and ASCL1 are mutally exclusive with HES1 based on immunostaining of prostate tissue sections containing early NE lesions (Supplemental Figure 4G). Overall, these data demonstrate that Notch signaling was downregulated during NEPC development in both mouse and human prostate cancer.

### Notch signaling reactivation suppresses NEPC development.

**Figure 2:**
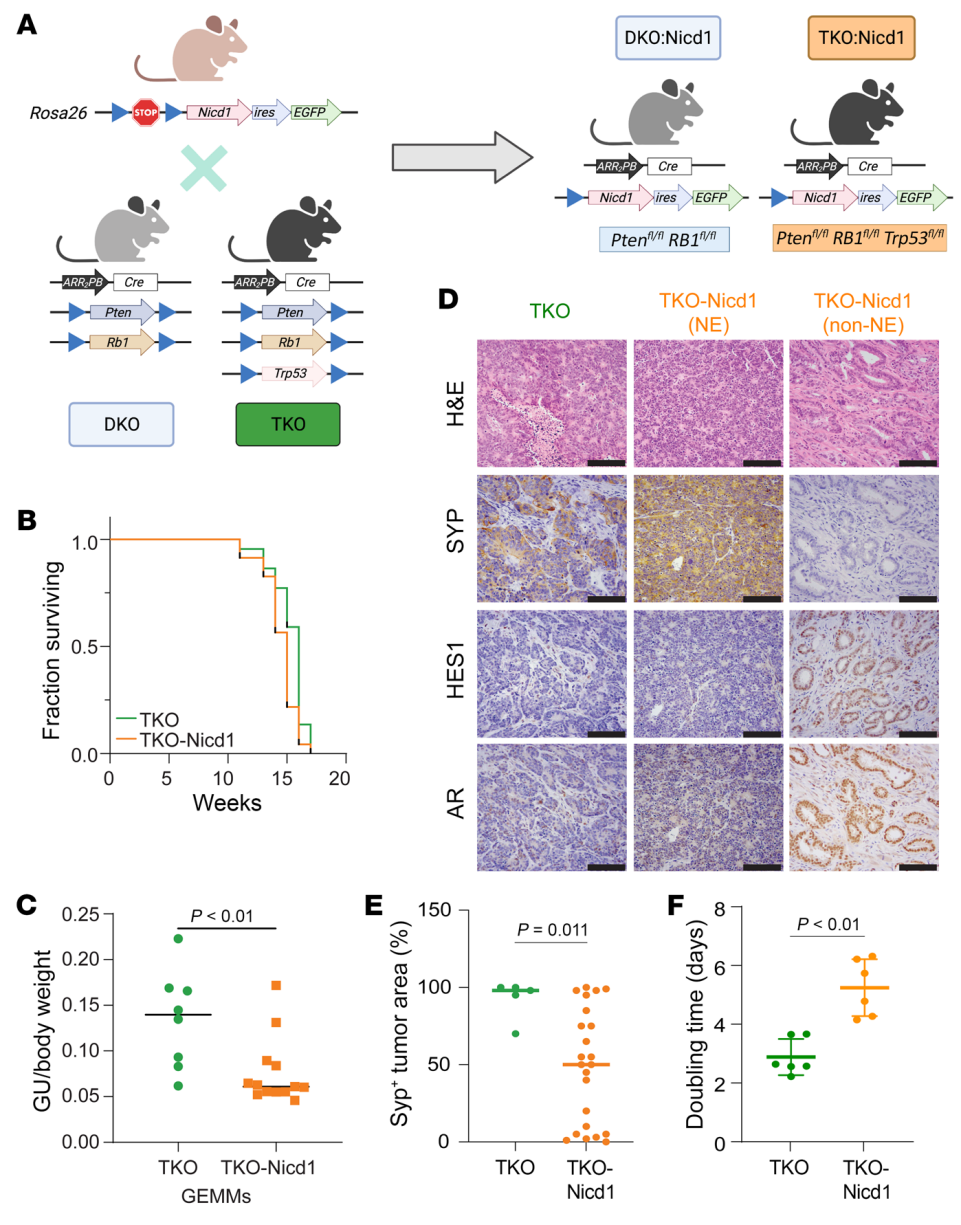
Evaluation of DKO-*Nicd1* and TKO-*Nicd1* GEMMs. (**A**) Schematic of DKO-*Nicd1* and TKO-*Nicd1* GEMMs. Both *Nicd1* and *EGFP* are expressed when the lox-*STOP-lox* cassette is deleted by probasin-driven Cre recombination. (**B**) Survival of TKO and TKO-*Nicd1* mice. The median survival was 15 weeks for TKO-*Nicd1* mice and 16 weeks for TKO mice (log-rank *P* = 0.025). (**C**) Ratio of GU weight/body weight of TKO and TKO-*Nicd1* mice. TKO-*Nicd1* mice had a significantly lower GU weight/body weight ratio (*P* < 0.01, by 2-tailed *t* test). (**D**) End-stage TKO and TKO-*Nicd1* tumors were immunostained for the indicated proteins. TKO tumors expressed SYP and had reduced levels of HES1 and AR. TKO-*Nicd1* tumors could be either NE or non-NE phenotypes. Scale bars: 50 μm. (**E**) Percentage of SYP^+^ tumor area in the indicated genotypes of end-stage mice. TKO-*Nicd1* mice had significantly smaller SYP^+^ areas than did TKO mice (*P* < 0.01, by Mann-Whitney *U* test). (**F**) The doubling time of tumor growth was calculated using a nonlinear regression method, which showed that TKO-*Nicd1* mice had a significantly longer doubling time than did TKO mice (*P* < 0.01, by Mann-Whitney *U* test).

To test whether Notch signaling regulates NE differentiation in prostate cancer, we utilized the *Rosa26*-loxP-STOP-loxP-*Nicd1*-*EGFP* allele that ectopically expresses the Notch1 intracellular domain (*Nicd1*) in mice after Cre-mediated recombination, constitutively activating Notch signaling independent of the ligand (44). Given the availability of Notch transgenes in GEMMs and that both *NOTCH1* and *NOTCH2* are downregulated in NEPC, we chose *Nicd1*. We introduced the *Nicd1* allele into DKO and TKO mice to generate DKO-*Nicd1* and TKO-*Nicd1* GEMMs that coupled Notch activation with tumor suppressor gene deletion in the prostate (Figure 2A and Supplemental Figure 5A). The overall survival of DKO-*Nicd1* mice was not significantly different from that of the DKO mice (median survival, 40 vs. 39 weeks) (Supplemental Figure 5B). Castration significantly extended the survival of the DKO mice (45 vs. 39 weeks, log-rank *P* = 0.01) as reported previously (7), but did not significantly extend the survival of the DKO-*Nicd1* mice (41 vs. 40 weeks) (Supplemental Figure 5C). However, survival data were confounded by large epididymal tumors that developed specifically in *Nicd1*-expressing mice (Supplemental Figure 5D). Epididymal tumors have been documented to form when PBCre4 driven *Nicd1* expression is combined with *Pten* deletion (30). Thus, the lifespan of both intact and castrated DKO-*Nicd1* mice may be limited by epididymal tumors rather than prostate cancer.

Indeed, prostate tumors were small or absent in DKO-*Nicd1* mice, failed to express NE markers such as SYP, and were composed primarily of lower-grade adenocarcinoma or intraductal neoplasia (Supplemental Figure 5E). Metastasis was not detected in DKO-*Nicd1* mice either by gross examination of dissected tissue or microscopic examination of tissue sections (0 of 17 mice examined). DKO-*Nicd1* mice progressing through castration did not show evidence of prostate cancer progression pathologically but instead exhibited large epididymal tumors. In contrast, DKO mice with end-stage disease (end-stage DKO mice) developed large, high-grade primary PCas that expressed NE lineage markers, metastasized with 100% penetrance, and progressed to NEPC after castration (7). These findings indicate that prostate cancer progression was markedly slower in the DKO-*Nicd1* mice than in the DKO mice.

TKO-*Nicd1* mice exhibited a small but statistically significant decrease in median survival compared with TKO mice (15 vs. 16 weeks, log-rank *P* = 0.025) (Figure 2B). Epididymal tumors were also detected in all TKO-*Nicd1* mice and likely confounded the survival data. To determine whether Notch signaling affected prostate tumor burden, we dissected the genitourinary (GU) tract, excluding epididymal tumors, and measured the GU/total body weight ratio (Figure 2C). Relative GU weight was significantly reduced in TKO-*Nicd1* mice compared with TKO mice (0.076 versus 0.134). However, end-stage TKO-*Nicd1* mice developed large prostate tumors with a range of different phenotypes including high-grade, NEPC-like tumors (NE) and low-grade adenocarcinomas lacking NE marker expression (non-NE) (Figure 2D). Non-NE cancer cells exhibited higher Notch signaling activity (i.e., high HES1 and NOTCH1/2 expression) and higher nuclear AR levels (Figure 2D and Supplemental Figure 5F). NE and non-NE cancer cells often existed in close spatial proximity, sometimes intermixing (Supplemental Figure 5G). NE cells exhibited loss of Notch signaling activity relative to nearby non-NE cells, as indicated by Notch target gene expression (e.g., *Hes1*). Based on EGFP expressed from the bicistronic *Nicd1* transgene, non-NE tumors had higher *Nicd1* transgene expression (Supplemental Figure 5H). The preponderance of NE and non-NE prostate cancer at end stage varied among individual mice, but some TKO-*Nicd1* mice had non-NE adenocarcinoma (KRT8^+^SYP^–^INSM1^–^) as the predominant end-stage tumor. NE and non-NE cells were approximately equivalent in TKO-*Nicd1* tumors (Figure 2E). In contrast, end-stage tumors in TKO mice were uniformly NEPCs, with NE lineage marker expression (KRT8^–^SYP^+^INSM1^+^), low Notch signaling, and low nuclear AR expression (Figure 2, D and E, and Supplemental Figure 5I) (7). TKO-*Nicd1* tumors metastasized to lymph nodes and the lung. These metastases were either NE or non-NE. Since non-NE cells in TKO-*Nicd1* mice still had metastatic potential, we queried if non-NE cells in metastatic lesions might possess stem-like features that have facilitated colonization and propagation (45) and found that the cancer stem cell marker CD44 (45–47) was highly expressed in TKO-*Nicd1* non-NE lung lesions but not in TKO primary NE cells (Supplemental Figure 6A). Liver metastasis was less frequent in TKO-*Nicd1* mice and exclusively had an NE phenotype (Supplemental Figure 6, B and C). The reduced NE primary tumor burden in TKO-*Nicd1* mice correlated with reduced liver metastasis compared with TKO mice, suggesting that non-NE prostate cancer developing in these mice did not metastasize to the liver efficiently (Supplemental Figure 6D). In contrast, all metastases detected in TKO mice were NE, as described previously (7).

Organoids from TKO-*Nicd1* prostate cancer tissue were established to better control for the variation in *Nicd1* transgene expression by flow-sorting cells for high or low EGFP expression. TKO-*Nicd1* organoids selected for high EGFP expression were transplanted into severe combined immunodeficiency disease (SCID) male mice and compared with TKO organoid transplants. Tumor growth was variable, but the TKO-*Nicd1* organoid tumors grew slower with significantly longer doubling time than did TKO organoid tumors (Figure 2F and Supplemental Figure 7A). We then implanted TKO-*Nicd1* organoids into either intact or castrated mice and observed that the androgen status did not significantly affect TKO-*Nicd1* tumor growth (Supplemental Figure 7B). Similar results were observed when organoids were implanted into female host mice to mimic low androgen status (Supplemental Figure 7C). The phenotypes of the resulting tumors were markedly different, however. All TKO organoid transplants (*n* = 13) developed NE tumors with low HES1 and AR immunostaining (Supplemental Figure 7D). In contrast, all TKO-*Nicd1* organoid transplants (*n* = 20) developed non-NE (ASCL1^–^) tumors with detectable nuclear HES1 and AR immunostaining (Supplemental Figure 7D). scRNA-Seq analysis indicated that transplant tumor cell transcription clustered by genotype, with smaller differences due to the sex of the host (Supplemental Figure 7E). While TKO tumor cells were primarily NE (ASCL1^+^), TKO-*Nicd1* organoid tumor cells were non-NE and expressed markers of prostate epithelium (*Krt5*^+^, *Krt8*^+^, *Hes1*^+^) (Supplemental Figure 7, F and G). TKO-*Nicd1* organoids selected for low EGFP expression (low NICD1 expression) were also transplanted into male hosts. These organoids developed NE tumors (*n* = 4) (Supplemental Figure 7H), confirming that reduced *Nicd1* transgene expression failed to prevent NEPC development. Consistent with GEMMs, both TKO-*Nicd1* and TKO organoid tumors metastasized to the lung, where they maintained the non-NE (high EGFP TKO-*Nicd1*) or NE (TKO) phenotype of the corresponding primary tumors (Figure 2D). In summary, data from both DKO-*Nicd1* and TKO-*Nicd1* GEMMs and organoids demonstrated that Notch signaling suppressed prostate cancer NE differentiation but had differential effects on prostate cancer progression depending on the genetic background.

### Notch signaling alters prostate lineage in human NEPC models.

**Figure 3:**
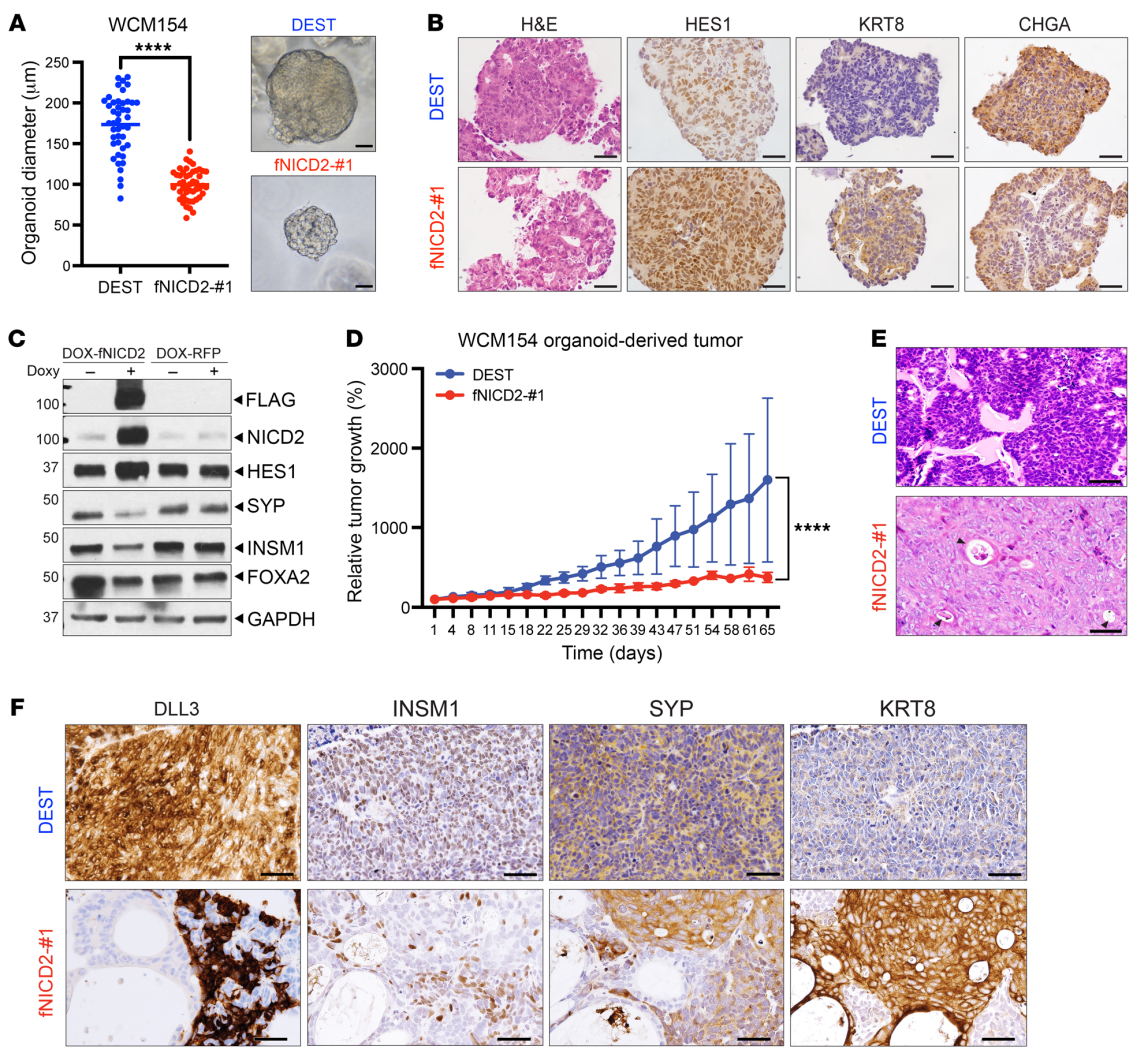
Restoration of Notch signaling in human NEPC models. (**A**) Bright-field images of WCM154-DEST (control) and fNICD2-#1 organoids. The size of an organoid was determined by its diameter on day 12. Ten images were taken from each WCM154-DEST and fNICD2-#1 organoid per biological duplicate, with 3 replicates in total. One or 2 organoids were measured per image. Each dot represents the size of 1 organoid (DEST: *n* = 44; fNICD2-#1: *n* = 47). ****P < 0.0001, by 2-tailed *t* test. Scale bars: 50 μm. (**B**) Immunostaining of WCM154-DEST and fNICD2-#1 organoids for HES1, KRT8, and CHGA. Scale bars: 50 μm. (**C**) fNICD2 expression was induced upon doxycycline treatment after 24 hours in WCM154-DOX-fNICD2 organoids, but not in DOX-RFP (control) organoids. Doxycycline-treated WCM154-DOX-fNICD2 organoids show increased HES1 and decreased SYP, INSM1, and FOXA2 levels. (**D**) WCM154-DEST and fNICD2-#1 organoids were implanted subcutaneously, and tumor volume measurements were initiated at 100 mm^3^ (*n* = 3 per group). The relative tumor size was normalized to day 1. ****P < 0.0001, by 2-way ANOVA. Data represent the mean ± SD. (**E**) H&E staining shows glandular like and luminal differentiation in fNICD2-#1 tumors indicated by arrows. Scale bars: 50 μm. (**F**) fNICD2-#1 tumor exhibits reduced levels of DLL3, INSM1, and SYP but increased KRT8 levels. Scale bars: 50 μm.

To investigate the role of Notch activation in prostate lineage determination in human NEPC models, we induced expression of a FLAG-tagged version of the NOTCH2 intracellular domain (fNICD2) under control of the CMV promoter in the previously described patient-derived NEPC organoid model WCM154 (48). We chose NICD2, as the NOTCH2 receptor was uniformly downregulated in NEPC patient cohorts (Supplemental Figure 1, A and B), and there were technical limitations using NICD1. To minimize fNICD2 heterogeneity, we performed single-cell selection to isolate clonal organoids. We noted that fNICD2 expression and HES1 target gene induction were variable across clones (Supplemental Figure 8, A and B) and chose clone fNICD2-#1 for subsequent studies. Ectopic fNICD2 expression significantly reduced the average organoid diameter (173 μm vs. 102 μm) and cell proliferation (Figure 3A and Supplemental Figure 8, C and D). Moreover, fNICD2 reduced expression of the NE markers SYP, CHGA (chromogranin A), FOXA2, and INSM1, while increasing expression of the luminal epithelial marker KRT8 and HES1 (Figure 3B and Supplemental Figure 8E).

fNICD2 was also expressed using a doxycycline-inducible transcriptional promoter in WCM154 organoids. Doxycycline efficiently induced fNICD2 expression in WCM154-DOX-fNICD2 organoids and downregulated ASCL1 as well as NE markers SYP, CHGA, FOXA2, and INSM1 (Figure 3C and Supplemental Figure 8F), concordant with results for constitutive fNICD2 expression. Moreover, cell death was not induced with fNICD2 (Supplemental Figure 8G). In control WCM154-DOX-RFP organoids, NE marker expression did not change after doxycycline treatment. We also induced the expression of fNICD2 in the NCI-H660 NEPC cell line, which resulted in reduced cell growth and downregulation of the NE markers INSM1, ASCL1, and FOXA2 after 24 hours of doxycycline exposure (Supplemental Figure 8, H and I). Expression of fNICD2 was induced as early as 1 hour after doxycycline, peaked at 24–48 hours, and then declined after 72 hours, in line with doxycycline’s half-life (Supplemental Figure 8J). NICD2 target genes such as *NOTCH1* and *HES1* exhibited similar dynamics over time. In contrast, expression of several NE lineage transcription factors showed an inverse expression pattern, decreasing upon fNICD2 induction and increasing as fNICD2 expression declined (Supplemental Figure 8J). SYP expression did not decline until 48 hours of doxycycline exposure, unlike INSM1 and NEUROD1, implying that some NE genes might be tightly influenced by Notch signaling but that others might be regulated through additional mechanisms.

To determine whether Notch signaling influences NEPC tumor development, we injected both WCM154-DEST (DEST) (control) and fNICD2-#1 organoids orthotopically into the anterior prostate of NSG mice. After 4 months, transplanted DEST organoids developed tumor masses of approximately 8–10 mm in length (100% take rate: 8 of 8), but fNICD2-#1 organoids did not form visible tumors (0 of 8) (Supplemental Figure 9A). The GU weight of mice transplanted with fNICD2-#1 was not significantly different than that of nontumor-bearing WT mice but was significantly less than that of mice transplanted with DEST control organoids (Supplemental Figure 9B). To allow for longer-term experiments, organoids were transplanted subcutaneously. fNICD2-#1 tumors grew 60% slower than did DEST tumors (Figure 3D). Analogous to the GEMMs, fNICD2 expression significantly restrained NEPC tumor growth in vivo.

The histological phenotypes of DEST and fNICD2-#1 transplant tumors exhibited distinct tumor lineages. DEST organoid–derived tumors displayed typical features of NEPC, with a high nucleus/cytoplasm ratio, granular chromatin, a trabecular growth pattern, and diffuse expression of NE markers (SYP and INSM1) as well as of Notch-inhibitory factors (ASCL1 and DLL3) (41) (Figure 3, E and F, and Supplemental Figure 9C). fNICD2-#1 tumors exhibited some tumor foci with a similar NE phenotype, but also harbored non-NE foci with adenocarcinoma-like features (Supplemental Figure 9, C and D). These non-NE foci displayed abundant cytoplasm, prominent nucleoli, multifocal glandular differentiation expressing the luminal markers (KRT8 and NKX3.1), and reduced expression of NE markers (SYP, INSM1, ASCL1, DLL3) (Figure 3F). Although fNICD2-#1 organoids were clonally derived, the tumors still displayed marked intratumoral heterogeneity, similar to the TKO-*Nicd1* GEMM tumors (Figure 2D).

**Figure 4:**
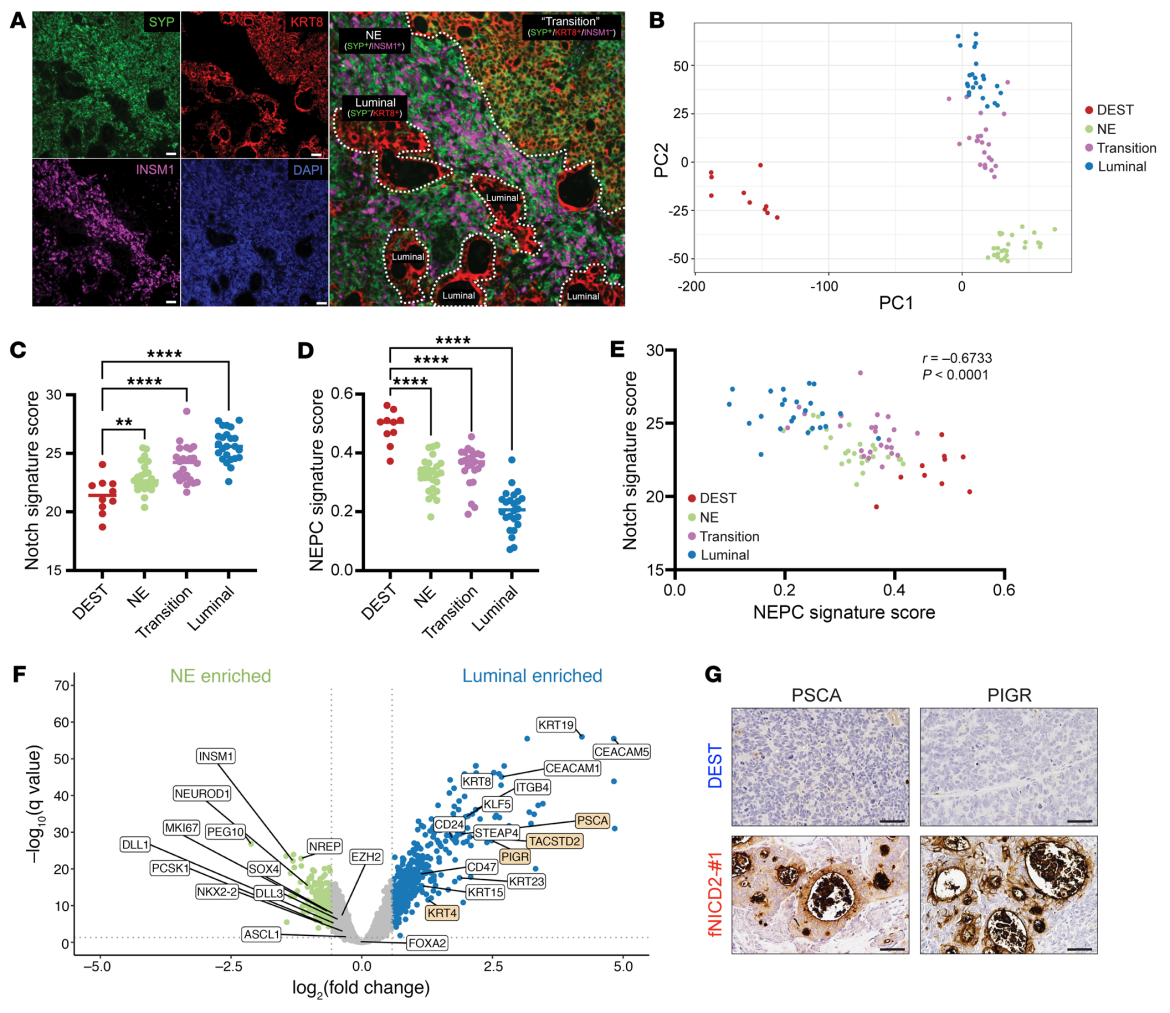
Notch signaling induces distinct lineages in human NEPC models. (**A**) Immunofluorescence staining of a fNICD2-#1 tumor for SYP (green), KRT8 (red), INSM1 (magenta), and DNA (blue). Three distinct lineages are highlighted by dashed lines. The NE lineage is labeled as SYP^+^INSM1^+^KRT8^–^; the transition lineage is labeled as SYP^+^INSM1^–^KRT8^+^; the luminal lineage is labeled as SYP^–^INSM1^–^KRT8^+^. Scale bars: 50 μm. (**B**) PCA differentiated transcriptomes of DEST tumors, NE, and transitional and luminal lineages of fNICD2-#1 tumors. (**C**) The Notch signaling and (**D**) NEPC signature scores were calculated for DEST and fNICD2-#1 tumors. **P < 0.01 and ****P < 0.0001, by one-way ANOVA. (**E**) Spearman’s correlation analysis showed a negative correlation between the Notch signaling score and the NEPC signature score (*r* = –0.6733, *P* < 0.0001). (**F**). Volcano plot indicates genes differentially expressed between the NE and luminal lineages within fNICD2-#1 tumors. (**G**) WCM154-DEST and fNICD2-#1 tumors were stained for the luminal markers PSCA and PIGR to confirm that fNICD2-#1 increased the expression of luminal markers. Scale bars: 50 μm.

To explore this heterogeneity further, we did multiplex immunofluorescence staining for select lineage markers and identified 3 distinct lineages: NE (KRT8^–^SYP^+^INSM1^+^), luminal (KRT8^+^SYP^–^INSM1^–^), and mixed/transition (KRT8^+^SYP^+^INSM1^–^) (Figure 4A). DLL3 expression was limited to NE tumor foci and was mutually exclusive of KRT8 expression (Supplemental Figure 9, E and F). Consistent with the findings in the mouse models, we also detected CD44 upregulation in the luminal lineage but not in the NE lineage regions (Supplemental Figure 9G). These data indicate that Notch signaling not only suppressed NE differentiation but also drove a more luminal and stem-like epithelial lineage state in human NEPC models. We conducted digital spatial profiling (DSP) of DEST and fNICD2-#1 tumors to evaluate heterogeneity at the RNA level (Supplemental Table 1). Principal component analysis (PCA) of transcriptomics data distinguished DEST and fNICD2-#1 tumors on PC1 and further separated the three fNICD2-#1 lineages on PC2 (Figure 4B). The Notch score was significantly higher and the NEPC score was significantly lower in fNICD2-#1 tumors compared with DEST tumors (Notch score: 24.32 vs. 21.4; NEPC: 0.3 vs. 0.5) (Supplemental Figure 10, A and B). Within the lineages detected, the Notch score was lowest in DEST tumors, followed by fNICD2-#1 NE and mixed/transitional lineage tumors and highest in the luminal lineage tumors (Figure 4C). Notch and NEPC scores were inversely correlated (Figure 4, D and E). Differential expression analysis identified significant enrichment of luminal genes (e.g., *KRT4*, *KRT8*, *PSCA*, *PIGR*) in the luminal lineage and NEPC-associated genes (e.g., *INSM1*, *NEUROD1*, *PEG10*) in the NE lineage regions (Figure 4F and Supplemental Figure 10C). Some NEPC-related genes (i.e., *ASCL1*, *DLL3*, *FOXA2*, *EZH2*) were higher in NE than in luminal lineage regions but did not reach statistical significance; this could be related to lower expression levels of these genes in fNICD2-#1 tumors compared with parental DEST tumors (Supplemental Figure 10C). When comparing the relative expression of NEPC-associated transcription factors in DEST versus fNICD2-#1 tumors, we found higher expression of *INSM1*, *PEG10* (paternally expressed 10), and *ONECUT2* (one cut homeobox 2) in DEST tumors. The mixed/transition lineage foci expressed similar transcription factors (e.g., *FOXA2*, *NKX2-2*) with intermediate levels of expression between the NE and luminal lineages (Supplemental Figure 10D). When examining published data sets (10, 35), genes highly expressed in luminal lineage foci from fNICD2-#1 tumors overlapped with genes highly expressed in benign prostate cancer compared with primary or metastatic prostate cancer (Supplemental Figure 10E). Moreover, among the human prostate luminal epithelial cell–type classifiers described previously (49), fNICD2-#1 luminal tumor foci expressed all 4 markers of luminal-C cells (TASCSTD2/PIGR/PSCA/KRT4) (Figure 4, F and G). Luminal-C prostate cancer cells were previously reported to be the potential cell of origin for NEPC in DKO and TKO GEMMs (43). Gene Ontology (GO) analysis indicated that these fNICD2-#1 tumor foci expressed genes related to lumen and granule formation (Supplemental Figure 10F). fNICD2-#1 NE tumor foci, in contrast, expressed genes related to neuronal developmental processes, neurogenesis, and nervous system development (Supplemental Figure 10G). These data suggest that Notch signaling suppressed NE differentiation in human NEPC, potentially returning cells to a type-C–like luminal cell phenotype from which NEPC may arise.

**Figure 5:**
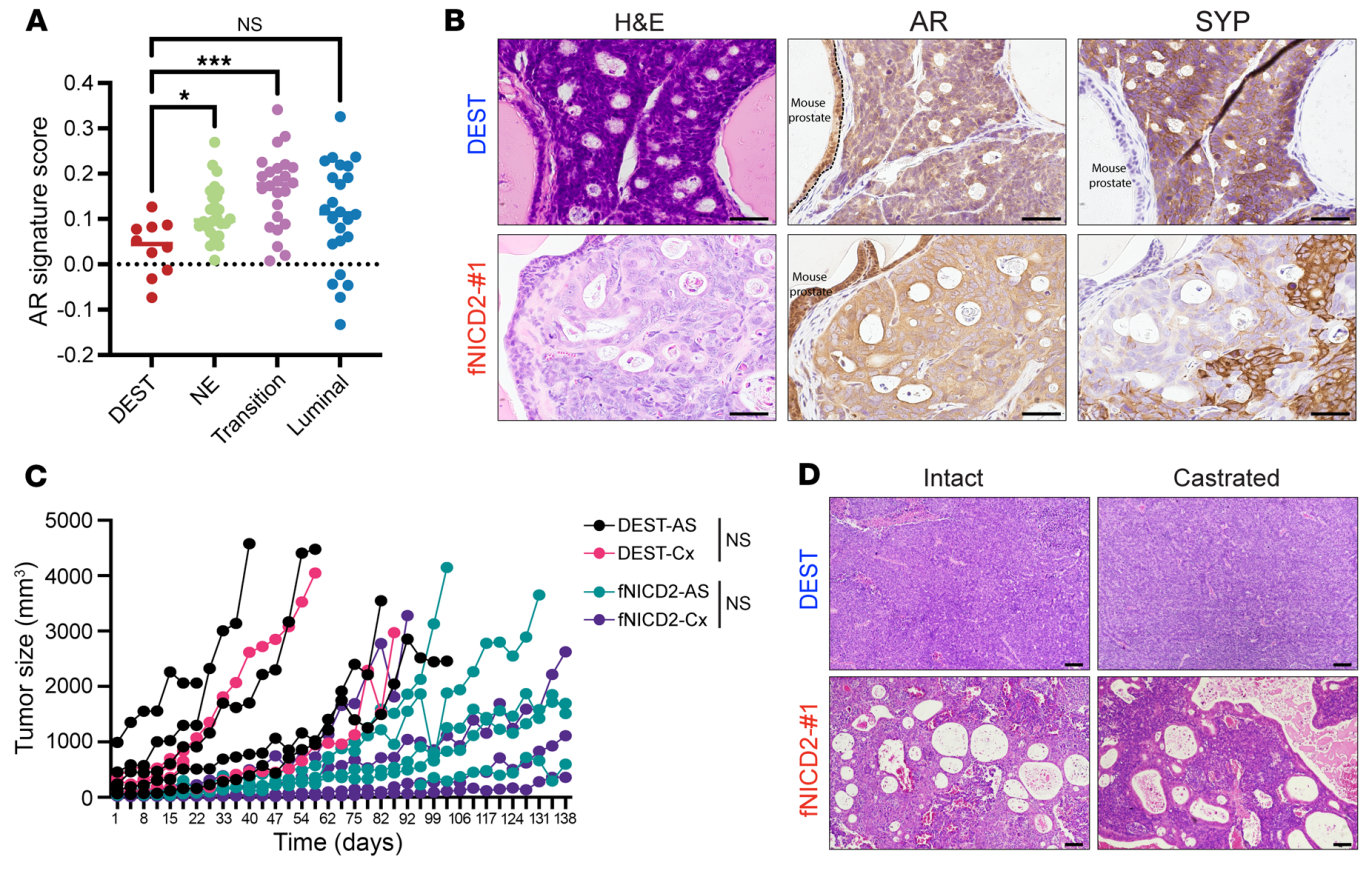
AR signaling in the WCM154-CMV-fNICD2 model. (**A**) AR signature scores were calculated in DEST tumor and NE, transitional, and luminal lineages of fNICD2-#1 tumors. *P < 0.05 and ***P < 0.001, by 1-way ANOVA. (**B**) DEST and fNICD2-#1 tumors were assessed for AR and SYP expression. Mouse prostate epithelial cells were used as an internal control to indicate positive nuclear AR staining and negative SYP staining. Scale bars: 50 μm. (**C**) DEST and fNICD2-#1 tumors were subcutaneously implanted into male mice. When tumor size reached approximately 100 mm^3^, half of the mice were surgically castrated. Tumor size was measured on the indicated days (*n* = 4–5 per group). A 2-way ANOVA was performed to test for significant differences between intact (AS) and castrated (Cx) mice for both DEST and fNICD2-#1. (**D**) Histology of intact and castrated DEST and fNICD2-#1 tumors. Scale bars: 100 μm.

Despite upregulation of select prostate luminal epithelial markers, canonical AR signaling was not significantly rescued in fNICD2-#1 luminal lineage tumor foci (Figure 5A). We confirmed a lack of nuclear AR protein expression in all 3 of the lineage phenotypes observed in the fNICD2-#1 tumors (Figure 5B). Consistent with this observation, there was no significant difference in the growth of fNICD2-#1 tumors in intact and castrated host mice (Figure 5C), although fNICD2-#1 tumors grew slower than DEST tumors in both intact and castrated hosts. Phenotypic differences between fNICD2-#1 tumors growing in castrated or intact mice were not detected. Histologically, tumors from both intact and castrated mice exhibited KRT8^+^NKX3.1^+^ luminal-like regions along with SYP^+^INSM1^+^ NE tumor foci (Figure 5D and Supplemental Figure 11). These data indicate that, while reactivation of Notch signaling in patient-derived NEPC organoids altered prostate cancer lineage phenotypes, the resulting lineage changes were not functionally linked to AR expression or AR signaling dependence.

### ASCL1 suppression activates Notch signaling in NEPC.

**Figure 6:**
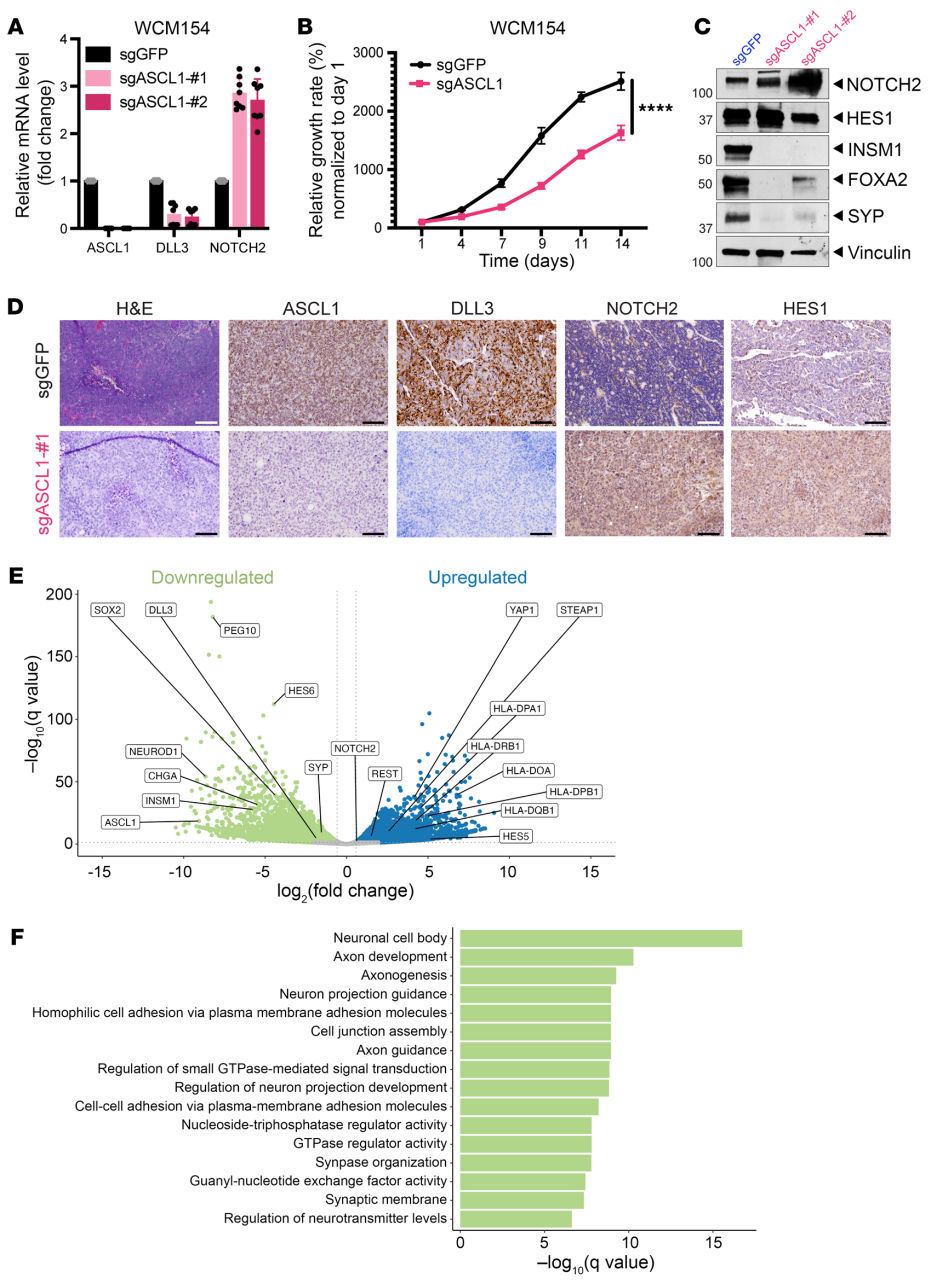
Deletion of *ASCL1* in the WCM154 model. (**A**) Relative mRNA levels of *ASCL1*, *DLL3*, and *NOTCH2* in WCM154-sg*GFP*, -sg*ASCL1*-#1, and -sg*ASCL1*-#2 organoids are shown. Standard deviations were measured from 3 independent replicates. (**B**) Growth of WCM154-sg*GFP* (control) and WCM154-sg*ASCL1* organoids was measured by CellTiter-Glo at the indicated time points and normalized to day 1. The data are from 3 biological replicates and represent the mean ± SD. ****P < 0.0001, by 2-way ANOVA. (**C**) Expression levels of Notch signaling markers (NOTCH2, HES1) and NE markers (INSM1, FOXA2, SYP) in *ASCL1*-KO organoids. (**D**) Histology of sg*GFP* and sg*ASCL1* organoid–derived xenografts. Tumor sections were stained with the Notch negative regulators ASCL1 and DLL3 and the positive regulators NOTCH2 and HES1 to indicate upregulated Notch signaling in the WCM154-sg*ASCL1* tumor. Scale bars: 100 μm. (**E**) Differential gene expression in sg*ASCL1* versus sg*GFP* tumors. Several NE transcription factors, such as INSM1 and PEG10, were downregulated in the sg*ASCL1* tumors. (**F**) GO analysis reveals enriched biological processes after *ASCL1* KO.

ASCL1 is a negative regulator of Notch signaling that drives DLL3 expression (20) and is overexpressed in a subset of poorly differentiated NE carcinomas including NEPC (16). To test whether suppression of *ASCL1* restores Notch signaling and also inhibits NE differentiation, we used CRISPR/Cas9 to target *ASCL1* in WCM154 organoids and then isolated single-cell clones lacking *ASCL1* expression (Figure 6A). Our data indicate that *ASCL1* KO (sgASCL1) significantly reduced NEPC organoid growth (Figure 6B), similar to previous reports (16). *ASCL1* KO also decreased the expression of NE markers including SYP, CHGA, FOXA2, and INSM1 (Figure 6C). We then implanted *ASCL1* KO WCM154 organoids into mice. *ASCL1* KO impeded tumor development compared with control organoids (Supplemental Figure 12A). *ASCL1* KO tumors developed a poorly differentiated carcinoma without detectable expression of the NE lineage markers SYP, INSM1, or DLL3 but showed higher expression of NOTCH2, HES1, and KRT8 (Figure 6D and Supplemental Figure 12B). These tumors did not have the glandular differentiation seen in fNICD2-#1 tumors. Similar to fNICD2-#1 tumors, *ASCL1* KO did not restore nuclear AR expression or exhibit evidence of AR signaling activity (Supplemental Figure 12B). Comparing bulk RNA-Seq data from *ASCL1* KO organoid transplant tumors with control sg*GFP* organoid–derived tumors, we found significant downregulation of genes and biological processes associated with neuronal functions (Figure 6, E and F) and upregulation of genes associated with rRNA processes (Supplemental Figure 12C). Since *ASCL1* KO upregulated Notch signaling and suppressed NE differentiation, we silenced *NOTCH2* in WCM154-sg*ASCL1* organoids to determine whether *NOTCH2* KO neutralizes this effect. We found that *NOTCH2* KO rescued INSM1 levels but did not change the expression levels of other NE markers such as FOXA2 (Supplemental Figure 12D). In addition, *NOTCH2* KO in WCM154-sg*ASCL1* organoids did not increase organoid growth (Supplemental Figure 12E), suggesting that *NOTCH2* KO was not sufficient to suppress all effects of *ASCL1* loss. Overall, these data further support the importance of the NOTCH/ASCL1 signaling axis as a critical determinant of NE differentiation in prostate cancer.

### Suppression of Notch signaling in CRPC.

**Figure 7:**
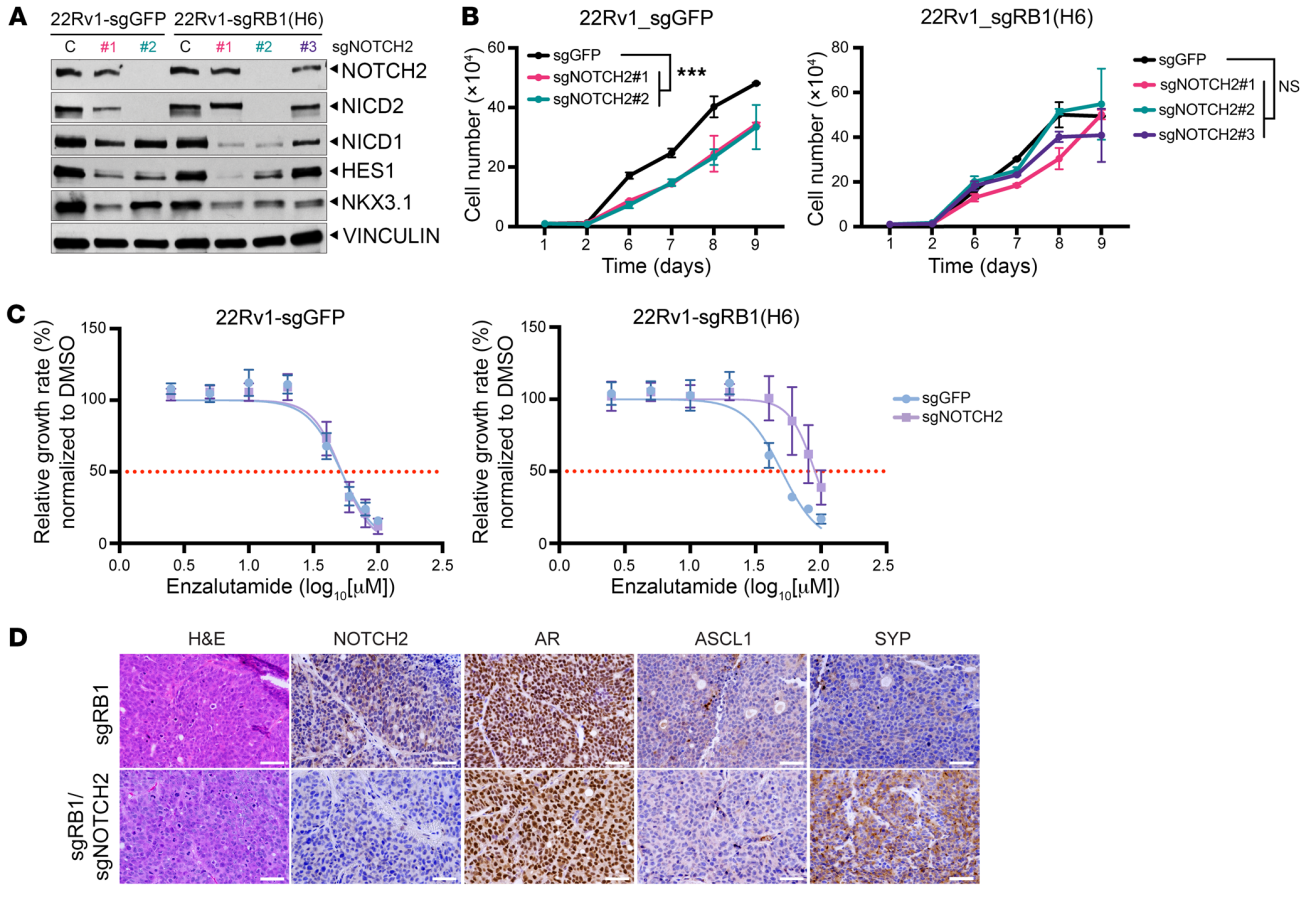
Deletion of *NOTCH2* in 22Rv1 cells in combination with *RB1* loss. (**A**) NOTCH2, NICD1, NICD2 and HES1 expression levels were reduced in 22Rv1-sg*NOTCH2* cells. The NE markers INSM1 and CHGA were undetectable in control (C) and *NOTCH2*-KO cells. (**B**) The growth of *NOTCH2*-KO 22Rv1 cells with (22Rv1-sg*GFP*) and without *RB1* (22Rv1-sg*RB1*) was measured using a hemacytometer. Data are from 4 technical replicates and 2 biological replicates and represent the mean ± SD. ***P < 0.001, by 2-way ANOVA. (**C**) 22Rv1 cells with or without *RB1* and *NOTCH2* loss were treated with DMSO (control) or enzalutamide with the indicated concentrations for 6 days. Relative cell growth was measured by CellTiter-Glo on day 6 and normalized to DMSO. The IC_50_ was determined using GraphPad and is shown on the graph. 22Rv1-sgGFP: 52.1 μM; 22Rv1-sg*RB1*/sg*NOTCH2*: 89.8 μM. The data are from 3 biological replicates with multiple technical replicates and represent the mean ± SD. (**D**) 22Rv1-sg*RB1* and 22Rv1-sg*RB1*/sg*NOTCH2* cells were subcutaneously transplanted into host mice. Tumors were stained with H&E, NOTCH2, AR, ASCL1, and SYP to characterize the phenotypes. Scale bars: 100 μm.

Our observations indicated that restoration of Notch signaling in NEPC can suppress cell proliferation and tumor growth, reduce NE differentiation, and induce luminal and glandular differentiation. This suggests that suppression of Notch signaling might drive NE differentiation in prostate adenocarcinoma. To test this, we used CRISPR/Cas9 to delete the *NOTCH2* gene in the AR^+^ CRPC cell line 22Rv1, with and without concurrent *RB1* deletion (Supplemental Figure 13A). *RB1* was deleted to facilitate plasticity, as suggested by prior studies (7, 8). We identified clones with validated gene deletions, and clones lacking NOTCH2 expression showed downregulation of NOTCH1 and HES1 as expected (Figure 7A and Supplemental Figure 13, B and C). *NOTCH2* loss significantly reduced cell growth (Figure 7B and Supplemental Figure 13D), consistent with a previous report indicating that Notch signaling is oncogenic in PCa (28). However, *NOTCH2* loss did not affect cell growth in 22Rv1 cells lacking *RB1* (Figure 7B and Supplemental Figure 13D), suggesting that NOTCH2-mediated signaling may no longer have been rate limiting for cell growth in the absence of *RB1*. We performed the same experiments in another AR^+^ CRPC cell line, C4-2. In C4-2 cells, *NOTCH2* KO did not affect cell growth (Supplemental Figure 13, E–G) or lead to upregulation of NE lineage markers (e.g., INSM1), either in the presence or the absence of *RB1*. *NOTCH2* loss did reduce the expression of the luminal epithelial markers NKX3.1 and KRT8 in 22Rv1 but not C4-2 cells (Figure 6A and Supplemental Figure 13, E and F). Control 22Rv1-sg*GFP* cells were modestly sensitive to the AR pathway inhibitor enzalutamide (IC_50_ = 52.1 μM) (Figure 7C), and 22Rv1 cells with *NOTCH2* KO and *RB1* loss had a reduced response (IC_50_ = 89.8 μM) (Figure 7C and Supplemental Figure 13H). Sensitivity to enzalutamide was not altered in C4-2 cells upon *NOTCH2* and *RB1* KO (Supplemental Figure 13I). Together, these data suggest that loss of *RB1* and *NOTCH2* might drive CRPC to become less AR dependent, even in the absence of NE differentiation in certain CRPC models.

We subcutaneously injected 22Rv1-sg*RB1* cells treated with sg*GFP* or sg*NOTCH2* into mice to examine tumor phenotypes in vivo. Both sg*RB1* and sg*RB1*/sg*NOTCH2* tumors demonstrated increased tumor growth compared with parental 22Rv1 cells (sg*GFP*) (Supplemental Figure 13J). We observed no significant morphologic phenotype changes upon *NOTCH2* deletion. Both control (sg*RB1*) and sg*RB1*/sg*NOTCH2* tumors expressed nuclear AR and SYP, but lacked expression of NE-associated transcription factors such as ASCL1 (Figure 7D). Overall, these data suggest that, although loss of Notch signaling may be important for regulating NE differentiation in prostate cancer, loss of Notch was not sufficient to drive NE differentiation of PCa even in the context of concurrent *RB1* loss.

### Notch signaling alters the prostate TME.

In small cell lung cancer (SCLC), different lineage subtypes have distinct responses to immunotherapy that correlate with differences in the tumor immune microenvironment (50). We observed marked changes in the prostate TME in conjunction with Notch-mediated changes in the prostate cancer lineage state. We detected tertiary lymphoid structures (TLSs), typically juxtaposed to areas of prostate cancer, in prostate tissue from all DKO-*Nicd1* mice in which this was examined (*n* = 7) (Supplemental Figure 14A). These TLSs contained cells expressing the lymphocyte markers CD3 and CD45, similar to what was observed in regional lymph nodes (Supplemental Figure 14B). TLSs were also detected in 16 of 20 (80%) SKO mice whose PCa also exhibited relatively high Notch signaling activity. Notably, TLSs were observed in only 8 of 22 DKO mice (36%) and 0 of 21 TKO mice whose prostate cancer had lower Notch signaling activity. A high frequency TLS development in SKO and DKO-*Nicd1* prostate cancer with high relative Notch signaling activity correlated with a lack of detectable metastasis at end stage.

**Figure 8:**
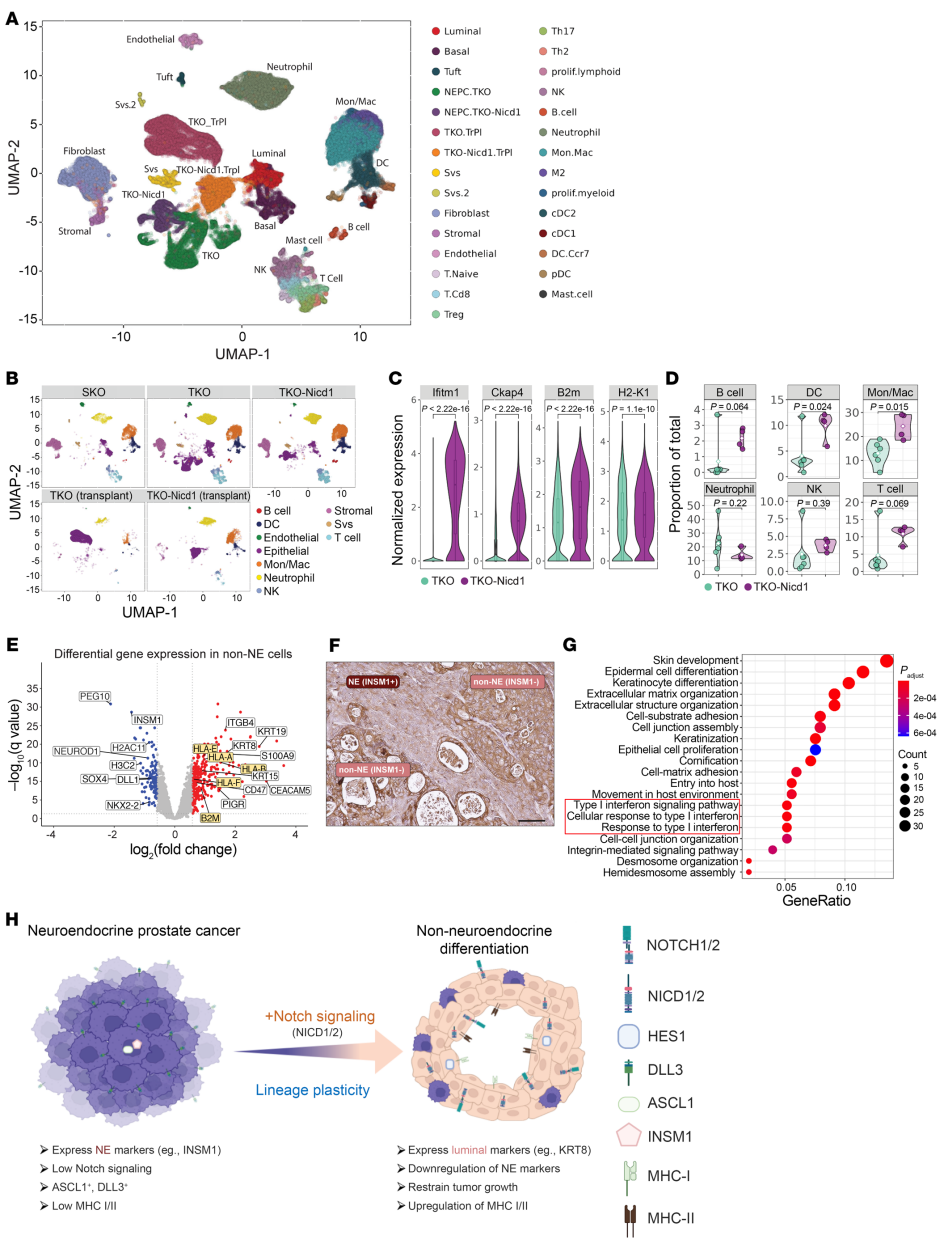
Notch-mediated prostate cancer lineage state influences the tumor immune microenvironment. (**A**) Prostate tissue from SKO (*n* = 3; 18,622 cells), TKO (*n* = 6; 19,485 cells), and TKO-*Nicd1* (*n* = 4; 19,253 cells) GEMMs or TKO (TKO.TrPl, *n* = 2; 11,918 cells) and TKO-*Nicd1* (TKO-Nicd1.TrPl, *n* = 2; 11,691 cells) transplant tumors were analyzed by scRNA-Seq, and the cells were clustered by transcriptional profile. The clusters are color coded on the basis of cell type as determined by the expression of cell-type–specific gene expression markers. UMAP, uniform manifold approximation and projection; prolif., proliferating. (**B**) The cell-type clusters are displayed for each genotype to compare relative cell-type composition of the samples. (**C**) Normalized expression of IFN/inflammatory (*Ifitm1*, *Ckap4*) and MHC genes (B2m, H2-K1) in neoplastic cells from TKO and TKO-*Nicd1* GEMMs was determined by scRNA-Seq (Supplemental Figure 15D). Wilcox tests were used to assess differences between genotypes, and the *P* values are shown. (**D**) The proportion of immune cell subtypes detected within TKO and TKO-*Nicd1* prostate tissue was calculated from scRNA-Seq data. A 2-tailed *t* test was used to assess the differences observed, the *P* values are shown. (**E**) Volcano plots depicting genes differentially expressed between NE and non-NE lineages developing in fNICD2-#1 transplant tumors. MHC-I genes (HLA-A, -B, -E, and -F) and B2M are highlighted, showing upregulation in non-NE cells. (**F**) A fNICD2-#1 transplant tumor section immunostained for HLA-ABC demonstrates upregulation at the protein level in cells with a non-NE lineage phenotype. Scale bar: 100 μm. (**G**) GSEA was performed using the spatial transcriptomics data in the luminal lineage, and type I IFN responses were identified. (**H**) Schematic of Notch signaling in NEPC. Notch signaling suppresses NE differentiation, drives non-NE lineage differentiation, and influences the immune microenvironment. Mon, monocytes; Mac, macrophages.

To test whether Notch signaling within prostate cancer cells influences the tumor immune microenvironment, we performed scRNA-Seq to analyze prostate tissue from SKO, TKO-*Nicd1*, and TKO GEMMs. We also profiled cells from TKO or TKO-*Nicd1* organoid transplant tumors. All cells were graphically clustered, and clusters were assigned to cell types on the basis of lineage-specific marker gene expression (Figure 8, A and B, and Supplemental Figure 15, A–C). Malignant cells from TKO and TKO-*Nicd1* GEMMs, or TKO and TKO-*Nicd1* organoid transplant tumors, mapped to distinct transcriptional clusters with minimal overlap (Supplemental Figure 15D), indicating that their gene expression patterns are largely distinct and variable. TKO-*Nicd1* cancer cells from GEMMs and transplant tumors had higher expression of genes related to inflammatory/IFN gene sets as well as genes relevant to MHC and antigen presentation (Figure 8C, Supplemental Figure 15, E–G, and Supplemental Tables 2–5).

Nonmalignant cells from the different genotypes had largely overlapping gene expression clusters that correlated with cell type, including immune cells, as expected. However, the relative proportion of some immune cells within TKO-*Nicd1* GEMM prostate tissue, including B cells, T cells, DCs, and NK cells, was significantly higher than in TKO tissue (Figure 8D). We noticed one outlier among control TKO mice that also exhibited increased immune cell infiltration (mouse ID T2789). The gene expression phenotype of prostate cancer in this mouse was similar to that in tumors developing in the TKO-*Nicd1* mice, potentially accounting for the higher infiltration of immune cells (Figure 8D and Supplemental Figure 15H). Although organoids were transplanted into immune-deficient mice, TKO-*Nicd1* transplant tumors contained more cells of the innate immune system remaining in these SCID mouse hosts compared with TKO transplant tumors (Supplemental Figure 15I).

In human NEPC models, NE and non-NE lineages (mixed/transition and luminal) of fNICD2-#1 tumors were compared, and MHC expression including HLA-A, HLA-B, and B2M was found to be significantly higher in INSM1^–^ non-NE cells (Figure 8E and Supplemental Figure 16A). This was confirmed at the protein level by immunostaining for HLA-ABC (Figure 8F). GO analysis indicated that gene expression relevant to type I IFN signaling was higher in non-NE cells (Figure 8G). Similar immunological changes were also observed in *ASCL1*-KO tumors (Supplemental Figure 16B). To further support these findings, we analyzed our patient data sets and found that expression of MHC-I and -II genes was lower in tumors of patient with NEPC than in CRPC-Adeno tumors and positively correlated with the Notch signaling score (Supplemental Figure 16, C–E). Gene set enrichment analysis (GSEA) also revealed that MHC-I, -II complex, and type I IFN signaling was relatively higher in CRPC-Adeno than in NEPC tumors (Supplemental Figure16, F and G). Overall, these data suggest that Notch signaling not only altered the prostate cancer lineage state, but had differential effects on the tumor immune microenvironment (Figure 8H).

## Discussion

Notch receptors are central components of an evolutionarily conserved signaling pathway essential for cell fate determination and physiological homeostasis (51, 52). This pathway plays an important role in the normal development of several organs including muscle, the heart, the hematopoietic system, the nervous system, the vasculature, and the pancreas (53). Notch signaling mediates divergent cell fates of neighboring cells through lateral inhibition enforced through feedback regulation. In the nervous system, Notch signaling is active in neural progenitor cells and maintains multipotency but is suppressed as cells commit toward terminal neuronal differentiation (9, 23, 53). NE carcinomas, including SCLC and NEPC, often express neuronal pathway genes reminiscent of mature neurons (54). In cancer, Notch signaling can have context-dependent and divergent functions (55–57). Notch signaling is oncogenic and promotes tumor development in some cancers, such as T cell acute lymphoblastic leukemia (57) and adenoid cystic carcinoma (58). Such findings have provided a rationale for developing drugs that block Notch signaling (56, 59). For some other cancers such as squamous cell carcinoma (21) and SCLC (21), NOTCH functions as a tumor suppressor, as indicated by recurrent loss-of-function *NOTCH1* gene mutations (21). Although Notch signaling can be upregulated and contribute to disease progression in PCa (28, 30), we found that Notch signaling was downregulated in NEPC and acted as a tumor suppressor. These findings have implications for understanding the molecular etiology of NEPC and the role of Notch signaling as a therapeutic target for prostate cancer.

We found that positive regulators and effectors of Notch signaling such as *NOTCH1*, *NOTCH2*, *HES1*, *REST* were downregulated in patients and preclinical models of NEPC. In contrast, the expression of negative regulators of Notch signaling including *ASCL1*, *DLL3*, and *HES6* was increased. Intermediate lineage states along the AR^+^/NE^–^ to AR^–^/NE^+^ continuum, including amphocrine and double-negative prostate cancers, did not show changes in these genes, suggesting that loss of Notch may be specific to the NE phenotype. The earliest emerging NEPC lesions detected in mouse models of NEPC demonstrated loss of Notch signaling activity. This tight inverse correlation between Notch signaling and NE differentiation suggests that the Notch signaling status may act as a key determinant of the NEPC lineage switch.

We generated relevant NEPC GEMMs and human models to decipher the role of Notch signaling in prostate cancer lineage plasticity. Although we did not compare NICD1 and NICD2 in the same model organisms due to experimental limitations, our study demonstrated remarkably similar results with NICD1 and NICD2, suggesting that lineage plasticity is not specific to a particular Notch receptor isoform. In the DKO-*Nicd1* GEMM, forced Notch signaling suppressed prostate cancer progression, as indicated by reduced primary tumor growth, a lower cancer grade, lack of detectable metastasis, and the absence of NEPC compared with DKO mice at the same age. The prostate cancer lineage state may be a key determinant controlling the prostate tumor growth rate, since DKO-*Nicd1* and DKO tumors have the same underlying tumor suppressor gene deletions but exhibit divergent lineage states and growth rates. Forced Notch signaling suppressed prostate cancer progression to a lesser extent in the TKO model. While Notch signaling in TKO-*Nicd1* did not completely suppress NEPC development or metastasis, heterogeneous tumors with both NE and non-NE components were observed. Some end-stage TKO-*Nicd1* mice exhibited a preponderance of non-NE tumor burden, a finding not observed in TKO mice. NE and non-NE prostate cancer cells developing in TKO-*Nicd1* mice were distinguished by differences in Notch signaling activity, likely caused by variation in *Nicd1* transgene expression. Indeed, TKO-*Nicd1* organoids selected for high transgene expression developed only non-NE tumors upon transplantation. Prostate cancer developing in a previously reported SKO-*Nicd1* mouse also showed reduced primary tumor growth but increased metastasis (30). Overall, these observations indicate that the effects of forced Notch signaling on prostate cancer progression vary depending on the tumor’s genetic background.

Activation of Notch signaling in human NEPC organoids and derived xenografts not only hindered tumorigenicity, but also suppressed NE differentiation and induced luminal-like morphologic and molecular features. Notch-expressing NEPC tumors harbored 3 spatially distinct lineage regions including NE, mixed/transition, and luminal-like regions. Although the NICD2 NEPC model was clonally derived, it is possible that the heterogeneity could result from asymmetric division (60), intrinsic silencing of NICD2, or potentially downstream epigenetic mechanisms. Spatial transcriptomics revealed that luminal-like tumor foci harbored a gene expression pattern reminiscent of type-C luminal cells. Human type-C luminal epithelial cells in the normal human prostate are analogous to mouse L2 luminal epithelial cells (61), implicated as the cell of origin of NEPC in DKO and TKO GEMMs (43). Our results, therefore, support the idea of reprogramming of NEPC cancer cells to a more luminal state resembling benign prostate and strengthen the evidence that type-C/L2 prostate luminal cells might serve as a cell of origin for NEPC. As normal type-C/L2 prostate luminal epithelial cells show relatively low AR signaling activity relative to other luminal cell types (49, 61), this potentially accounts for our observation that Notch-mediated suppression of NE differentiation did not restore canonical AR signaling activity or AR signaling dependence in either mouse or human experimental models.

How Notch signaling switches from being oncogenic in PCa to tumor suppressive in NEPC warrants further study. This signaling could be via downstream dysregulation of NE-associated transcription factors (12, 15, 16) or by abrogating the cell-cycle progression seen in other cancer types (21, 62, 63), with differential effects influenced by underlying *RB1* and *TP53* loss. Notch signaling inhibited prostate cancer progression to a greater extent in DKO-*Nicd1* mice than in TKO-*Nicd1* mice, potentially because Notch signaling can induce p53 activation and apoptosis (30). When we suppressed *NOTCH2* expression and Notch signaling activity in 22Rv1 and C4-2 CRPC-Adeno cells, NE differentiation was not induced. Thus, loss of Notch signaling may be necessary for maintaining NE differentiation in prostate cancer, but not sufficient to drive NEPC development in the CRPC models we tested. However, it cannot be excluded that sufficient residual Notch signaling remained in these experimental models to prevent NE differentiation.

ASCL1 is a negative regulator of the Notch pathway and is oncogenic in several poorly differentiated NE carcinomas (16, 64). Our study indicates that genetic ablation of *ASCL1* in NEPC organoids not only affected organoid and tumor growth as expected, but also restored Notch signaling and suppressed NE differentiation. Notably, subsequent knockout of *NOTCH2* did not rescue the effect of *ASCL1* loss. While *ASCL1*-deficient NEPC tumors lost their NE features, they did not acquire the luminal-like features that were seen with Notch activation, suggesting that the roles of Notch signaling and ASCL1 were not completely overlapping. Future studies evaluating the distinct downstream targets of NOTCH (NICD1/-2 and cofactor RBPJ) versus ASCL1 are warranted.

As inhibition of Notch signaling in PCa models was not sufficient to induce NE differentiation, luminal and NE differentiation pathways are not inexorably linked and may be modulated independently. Activation of Notch in NEPC (NE^+^AR^–^) models resulted in a non-NE (NE^–^AR^–^) luminal-like state, which has disease parallels to the clinical observations of double-negative (NE^–^AR^–^) CRPC (6, 65). Future work will be required to test whether this reflects a state within the continuum of transdifferentiation.

Understanding how and when Notch signaling is downregulated during lineage plasticity is important when thinking about how to leverage this pathway therapeutically. While mutations involving Notch pathway genes are not typically seen in prostate cancer, including NEPC (66, 67), epigenetic modifications associated with the suppression of Notch signaling genes may be observed. Previous reports have also pointed to hypoxia as a regulator of NE differentiation and Notch signaling (14, 68). Targeting this dysregulated pathway is also of relevance, as DLL3-targeted T cell engagers are showing promising clinical activity in patients with SCLC, NEPC, or other NE carcinomas (69, 70). While DLL3 is expressed in the majority of NEPC, it is also expressed in up to 12% of CRPC-Adeno tumors, albeit more focal and associated with NE markers and *RB1* loss (19); it is possible that DLL3 focal expression in these cases may represent foci of early lineage plasticity. Our results also suggest that therapeutic manipulation of Notch with Notch inhibitors should be deployed with caution, depending on the nature of the prostate cancer (adenocarcinoma versus NEPC), given its context-dependent function.

Little is currently known regarding the effect of prostate cancer lineage plasticity on the TME. Collectively, our data are consistent with a model of prostate cancer progression from a luminal-differentiated lineage state toward an intermediate, more plastic, stem-like lineage state in which inflammatory genes are expressed and, finally, to a differentiated NE state with low inflammatory gene expression (43). It is likely that Notch signaling is controlled by cell-to-cell contact within the TME to help drive these transitions. Activation of Notch signaling within NEPC cells increases the expression of MHC genes and genes involved in type I IFN and inflammatory signaling. These expression changes correlate with infiltration of immune cells into tumor-bearing prostate tissue, including the formation of tertiary lymphoid structures in some cases. These results are consistent with both preclinical and clinical data in SCLC, in which non-NE tumors with high Notch expression associate with higher MHC and IFN-α/β levels (54, 71). Immunotherapy has yielded modest success in prostate cancer, as it is considered to be an immunologically cold tumor. In SCLC, another relatively cold tumor, plasticity from a NE to a non-NE state has been associated with inflammatory changes as well as an immunotherapeutic response to checkpoint inhibitors. The link between prostate cancer lineage plasticity and the tumor immune microenvironment discovered here may therefore point to new opportunities to leverage the cancer lineage state to improve antitumor immunity.

## Methods

### Sex as a biological variable.

Since prostate cancer occurs in males, all human tissue, data, and models were derived from men. For in vivo experiments, male mice were used except for select experiments, where female mice were used to assess tumor growth in the absence of androgens.

### Clinical RNA-Seq data sets.

The Beltran, International SU2C/PCF Dream Team, LuCaP PDXs, and FHCRC RNA-Seq data sets were previously published (GSE147250, GSE126078) (35–37, 39). Methods for the 70-gene NEPC score and the 30-gene AR signaling score were previously described (35, 72), and values were derived from original publications (36, 37). The Notch score was calculated using log_2_-transformation (1+ reads per kilobase per million mapped reads [RPKM] RNA-Seq reads) of 19 genes involved in canonical Notch signaling (Figure 1A), multiplying dictional factors (+1 = positive regulators; –1 = negative regulator) for each gene (73). The Notch score was validated in a SCLC study of Hes1^+^ (high Notch) and Hes1^–^ (low Notch) models (Supplemental Figure 1C).

### GEMMs.

To generate DKO-*Nicd1* and TKO-*Nicd1* GEMMs, the *Rosa26-loxP-STOP-loxP-Nicd1-EGFP* allele (44) (The Jackson Laboratory [JAX] stock no. 008159) was bred into DKO and TKO mice (7). Experimental mice were on a mixed C57BL/6:129/Sv:FVB genetic background. Mice were monitored daily, euthanized when exhibiting signs of morbidity, and necropsied to verify the diagnosis and collect tissue. Survival analysis was done with the Kaplan-Meier method using GraphPad Prism software (version 9.5.1).

### scNA-Seq analysis.

scRNA-Seq was performed as previously described (43). Mice were euthanized, and half of each prostate, including 1 of each paired lobes, was pooled for tissue dissociation. Tissue was dissociated with collagenase II (ThermoFisher) in media supplemented with 10 μM Y-27632 ROCK inhibitor (Selleck) and 1 nM R1881 (AbMole Bioscience), followed by trypsinization (ThermoFisher, 0.25%). Filtered cell suspensions were sorted for cell viability using DAPI staining, and then cell suspensions were counted after trypan blue staining using a Countess FL automated cell counter (Thermo Fisher Scientific). scRNA-Seq was performed using the 10X platform according to the manufacturer’s recommendations. The resulting sequencing libraries were evaluated on D1000 screentape using a TapeStation 4200 (Agilent Technologies) and quantitated using a Kapa Biosystems qPCR quantitation kit. The resulting library pools were sequenced on a NovaSeq 6000 following the manufacturer’s protocol (Illumina). Data analysis is detailed in the Supplemental Methods.

### Organoids, cell culturing, and growth assays.

Patient-derived organoid culture and seeding methods were described previously (48). The 22Rv1, C4-2, and NCI-H660 cell lines were purchased from American Type Culture Collection (ATCC) and cultured according to the manufacturer’s protocols. Cell authentication was performed using short tandem repeat (STR) analysis, and cells were routinely tested for mycoplasma (InvivoGen). For growth assays, 3,000 cells per well were plated in a 96-well plate for the different time points indicated in the figure legends. Relative cell growth was measured by CellTiter-Glo (Promega) per the manufacturer’s protocol and normalized to day 1. All growth experiments were conducted at least twice biologically with multiple technical replicates. Mouse organoids were generated from TKO or TKO-*Nicd1* prostate tumor tissue isolated from mice at end stage. Tissue was dissociated and cell suspensions cultured in mouse prostate organoid media as described previously (74, 75). For some TKO-*Nicd1* prostate cancer organoids, cell suspensions were flow sorted to select for high or low cells expressing high or low levels of EGFP before organoid culturing.

### FLAG-tagged NICD2-expressing models.

A FLAG-tagged *NICD2* ORF was subcloned into a destination vector pLenti CMV Puro DEST (Addgene plasmid #17452) using Gateway LR Clonase II Enzyme mix (Thermo Fisher Scientific) and then delivered into WCM154 organoids by lentiviral infection. Details can be found in the Supplemental Methods.

### CRISPR/Cas9-KO models.

CRISPR/Cas9 constructs were generated following a published protocol (76) using lentiCRISPR v2 (Addgene plasmid 52961) (see the Supplemental Methods). The sgRNAs sequences targeting *RB1*, *ASCL1*, and *NOTCH2* are listed in Supplemental Table 6.

### Histology.

Tumor tissues and organoids were fixed in 10% neutral buffered formalin, paraffin embedded, and serially sectioned at 4 μm thickness. Consecutive sections were used; when not feasible, sections were still derived from the same batch of experiments and tumor blocks. Sections were deparaffinized in xylene solution and gradually rehydrated in ethanol, and then stained for assessment of histopathology. Results were verified by board-certified GU pathologists. The IHC details are described in the Supplemental Methods. Each image is displayed only once in this study.

### Multiplex immunofluorescence.

Formalin-fixed, paraffin-embedded (FFPE) slides were incubated sequentially with primary antibodies and fluorescence-conjugated secondary antibodies using Tyramide SuperBoost Kits (Thermo Fisher Scientific). Then, slides were stained with NucBlue DAPI (Thermo Fisher Scientific) and mounted with VECTASHIELD Vibrance Antifade Mounting Medium (Vector Laboratories). Slides were imaged within 1 week using the NIS-Elements imaging system (Nikon). Antibodies are listed in Supplemental Table 7, and additional details can be found in the Supplemental Methods.

### Immunoblotting.

The immunoblotting experiments are described in the Supplemental Methods. Each immunoblot image in this study is presented only once, with no duplicate images.

### In vivo studies.

To establish prostate orthotopic transplants, 2 cm incisions were made in the lower part of the abdomen of male mice. A total of 50 μL organoid-Matrigel mix (1:1, Corning) containing 2 × 10^5^ organoid cells was injected into the anterior prostate. The incision was then sutured and clipped. Mice were euthanized after 4 months to assess tumor development. To evaluate the response to androgen deprivation, 1 × 10^7^ organoids were subcutaneously injected into mice to generate donor tumors. When the donor tumor reached 1,000 mm^3^, the tumors were collected, and 2–5 mm single tumor pieces were subcutaneously implanted into mice. When the tumor size reached 100 mm^3^, the mice were randomized. Half of the mice were surgically castrated, and half were left intact. Tumor size was measured by a digital caliper twice a week and calculated using the following formula: volume (*V*) = length (*L*)^2^ × width (*W*) × 0.5. For the 22Rv1 models, 5 × 10^6^ cells mixed with an equal volume of Matrigel (Corning) were subcutaneously injected into castrated mice. Eight-week-old male NOD.Cg-*Prkdc^scid^* Il2rg*^tm1Wjl^*/SzJ (NSG) mice (The Jackson Laboratory) were used for the entire study. The number of mice for each experiment is indicated in the figure legends. For TKO and TKO-Nicd1 organoid xenografts, EGFP^hi^ organoids were sorted, and then 5 × 10^6^ organoid cells were subcutaneously injected into the right flank of male SCID mice. Tumor size was measured with a caliper over time.

### DSP.

FFPE slides (4 μm thick) were freshly sectioned. The first slide was subjected to immunofluorescence staining for SYP, KRT8, and INSM1 to identify tumor lineages. Stained slides were loaded onto a GeoMx instrument (NanoString) and scanned. Twenty-five ROIs (500 μm diameter per lineage) were selected and annotated to guide the locations for RNA-Seq. The following slide was then subjected to a GeoMx Human Whole Transcriptome Atlas (WTA) (NanoString) assay. Briefly, slides were deparaffinized, rehydrated, and hybridized with WTA probes in the oven overnight. After washing in 2× SSC buffer to remove off-target probes, the slides were loaded onto GeoMx DSP to collect ROIs for next-generation sequencing.

### Statistics.

Statistics were performed using GraphPad Prism software (version 9.5.1) and included 1-way and 2-way ANOVA, Spearman’s correlation analysis, log-rank test, Mann-Whitney *U* test, 2-tailed *t* test. Data are presented as the mean ± SD. *P* values of less than 0.05 were considered significant.

### Study approval.

All experiments were performed in compliance with IACUC guidelines at Dana-Farber Cancer Institute (protocol no. 18-020) and Roswell Park Comprehensive Cancer Center (842M, 1341M) and the IRB at Dana-Farber Cancer Institute (protocol no. 19-883).

### Data availability.

Raw and processed sequencing data were deposited in the Gene Expression Omnibus (GEO) database (scRNA-Seq of TKO GEMMs: GSE210358; scRNA-Seq of TKO-*Nicd1* GEMMs, TKO, and TKO-*Nicd1* transplant tumors: GSE235036; WCM154-sg*GFP* and WCM154-sg*ASCL1* bulk RNA-Seq: GSE234819). DSP data for WCM154-DEST and fNICD2-#1 tumors are provided in Supplemental Table 1. GEMM scRNA-Seq data are presented in Supplemental Tables 2–5. Raw data are disclosed in the Supporting Data Values file.

## Author contributions

SYK, DWG, and HB conceived and designed the study. SYK, YW, MMG, YY, MC, and MJK performed in vitro and in vivo experiments. SYK, YW, MMG, MAA, LP, MKB, VBV, and KMW performed data analysis. KM, MDL, and SR conducted bioinformatics analysis. MAA, BDR, AMA, and JMM reviewed the pathology. SYK, DWG, and HB wrote the original draft of the manuscript. SYK and YW conducted most of the experiments and contributed equally to the work. SYK also designed the study and wrote the manuscript and is thus listed as first co-author. All authors contributed to the writing and editing of the revised manuscript and approved the manuscript. DWG and HB obtained funding and supervised the work.

## Supplementary Material

Supplemental data

Unedited blot and gel images

Supplemental table 1

Supplemental table 2

Supplemental table 3

Supplemental table 4

Supplemental table 5

Supplemental table 6

Supplemental table 7

Supporting data values

## Version 1 07/18/2024

In-Press Preview

## Version 2 09/03/2024

Electronic publication
