# Supplementary material for: Notch signaling suppresses neuroendocrine differentiation and alters the immune microenvironment in advanced prostate cancer
Source: J Clin Invest. 2024 Jul 18;134(17):e175217. doi: 10.1172/JCI175217 (PMC11364388; doi:10.1172/JCI175217)
Supplement: Supplemental data [file jci-134-175217-s226.pdf]

Supplemental Material

Notch signaling suppresses neuroendocrine differentiation and alters the immune microenvironment in advanced prostate cancer

Sheng-Yu Ku[1], Yanqing Wang[2], Maria Mica Garcia[1], Yasutaka Yamada[1], Kei Mizuno[1], Mark D. Long[3], Spencer Rosario[2,3], Meenalakshmi Chinnam[2], Majd Al Assaad[4], Loredana Puca[5], Min Jin Kim[1], Martin K. Bakht[1], Varadha Balaji Venkadakrishnan[1], Brian D. Robinson[4], Andrés M. Acosta[6], Kristine M. Wadosky[2], Juan Miguel Mosquera[4], David W. Goodrich[2,7], Himisha Beltran[1]

[1]Department of Medical Oncology, Dana-Farber Cancer Institute, Boston, MA, USA
[2]Department of Pharmacology and Therapeutics, Roswell Park Comprehensive Cancer Center, Buffalo, NY, USA
[3]Department of Biostatistics and Bioinformatics, Roswell Park Comprehensive Cancer Center, Buffalo, NY, USA
[4]Department of Pathology and Laboratory Medicine, Weill Cornell Medicine, New York, NY, USA
[5]Department of Medicine, Weill Cornell Medicine, New York, NY, USA
[6]Department of Pathology, Brigham and Women's Hospital, Boston, MA, USA
[7]Department of Urology, Roswell Park Comprehensive Cancer Center, Buffalo, NY, USA

This PDF file includes:

Supplemental Figures 1-17
Supplemental Methods
Supplemental Tables

Supplemental Figures

Supplemental Figure 1

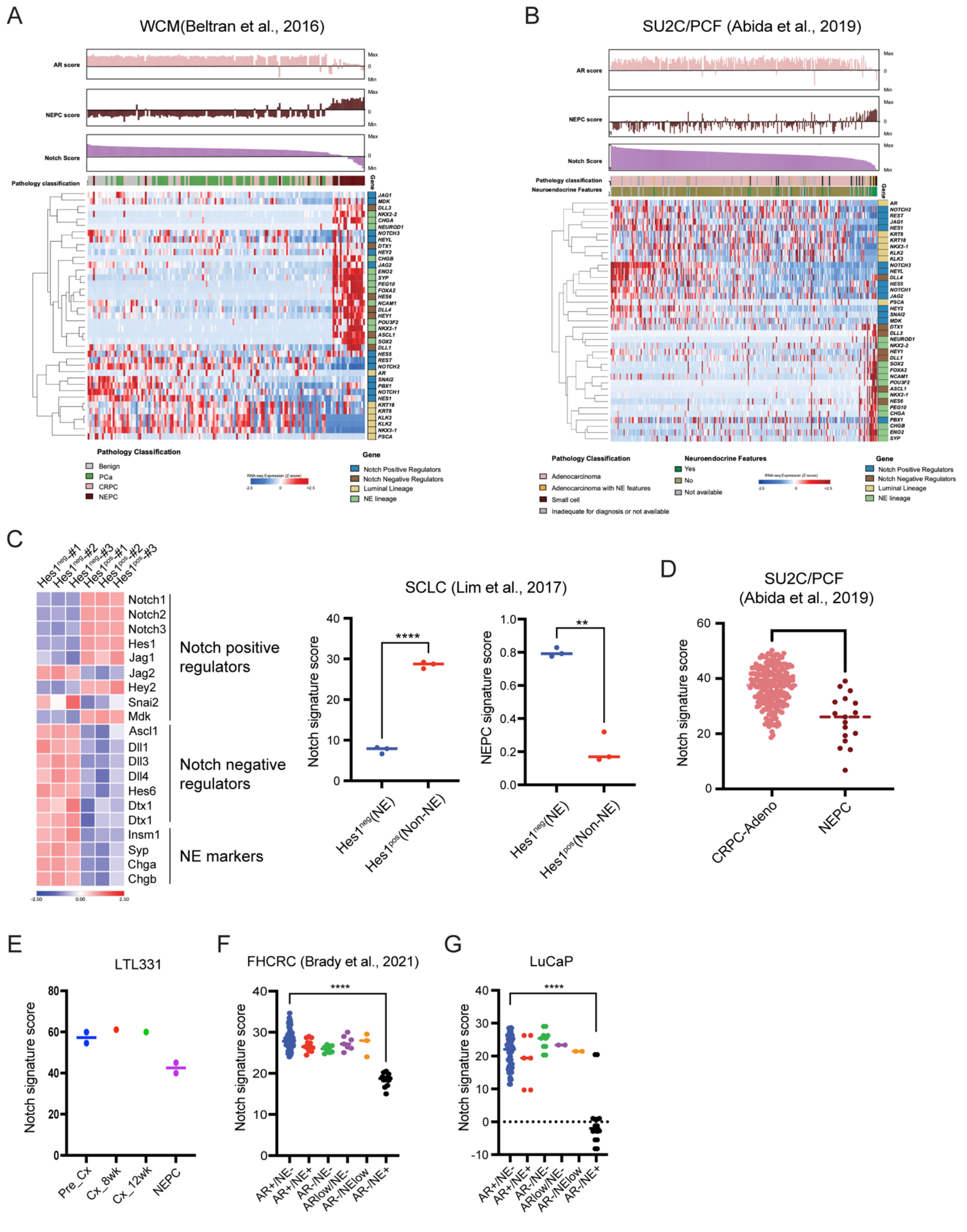

Supplemental Figure 1. Notch signaling in clinical datasets. Heatmap of Notch signaling, luminal markers, and neuroendocrine (NE) markers in the Beltran dataset **(A)** and SU2C/PCF dataset **(B).** Heatmaps generated using morpheus software. **(C)** Validation of Notch signaling signature score and NEPC score in SCLC cells with and without Hes1 overexpression. **(D)** Notch signaling score in CRPC-Adeno (n=218) and NEPC (n=17) patient tumors in the SU2C/PCF dataset. CRPC-Adeno was classified as adenocarcinoma pathologically; NEPC included mixed adenocarcinoma /NE carcinoma and small cell NE carcinoma. Statistical significance determined by the Mann-Whitney test (****$p<0.0001$). **(E)** Notch signaling score in the LTL331 patient derived xenograft (PDX) model that transdifferentiates from adenocarcinoma (Pre-Cx) to NEPC after androgen deprivation (1). **(F)** Notch signaling score in subtypes of prostate cancer in the FHCRC dataset. AR-/NE+ has significantly lower Notch score compared with the AR+/NE- subtype (One-Way ANOVA, ****$p<0.0001$). There was no significant differences observed within other subtypes. Sample size for each subtype: AR+/NE- (n=99); AR+/NE+ (n=12); AR-/NE- (n=8); ARlow/NE- (n=7); AR-/NElow (n=3); AR-/NE+ (n=13). **(G)** Notch signaling score in LuCaP PDX models(2). AR-/NE+ PDX tumors had significantly lower Notch score versus the AR+/NE- subtype (One-Way ANOVA, ****$p<0.0001$). Sample size for each subtype: AR+/NE- (n=37); AR+/NE+ (n=3); AR-/NE- (n=5); ARlow/NE- (n=1); AR-/NElow (n=1); AR-/NE+ (n=8).

Supplemental Figure 2

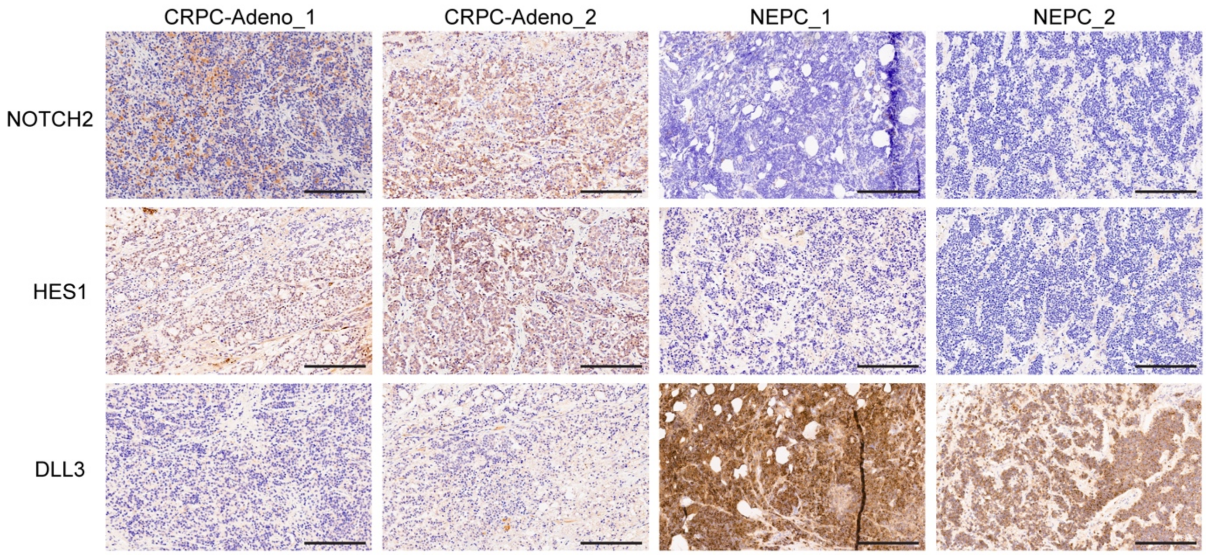

Supplemental Figure 2. Expression of Notch signaling in clinical specimens. An independent cohort of CRPC-Adeno and NEPC patient tumor samples was stained by immunohistochemistry to evaluate protein expression of the indicated markers. Two CRPC-Adeno and two NEPC cases are represented. Scale bar is 200μm.

Supplemental Figure 3

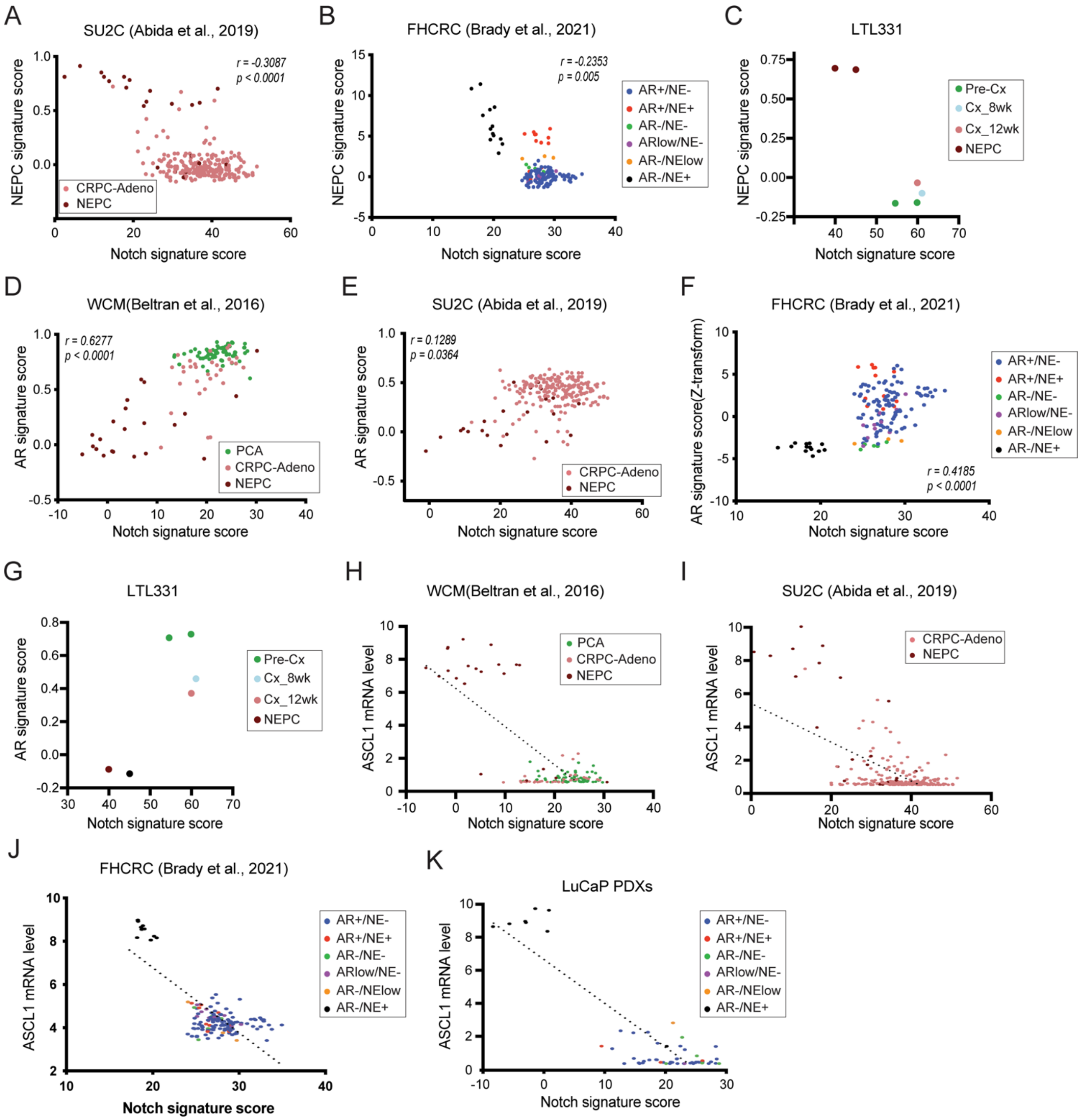

Supplemental Figure 3. Correlations with Notch signature score in clinical datasets. (A-C) Correlation of Notch signaling score and NEPC score in SU2C/PCF (Spearman: r=-0.3087, p<0.0001), FHCRC (Spearman: r=-0.2353, p=0.005), and LTL331 datasets. **(D-G)** Correlation of Notch signaling score and AR signaling score in the Beltran (Spearman: r=0.6277, p<0.0001), SU2C/PCF (Spearman: r=0.1289, p=0.0364) and FHCRC (Spearman: r=0.4185, p<0.0001) datasets. AR signaling scores in SU2C/PCF and FHCRC were derived from the original reports. **(H-K)** Correlation of Notch signaling score and *ASCL1* mRNA expression in the Beltran (Pearson: r=-0.78, p<2.2e-16), SU2C/PCF (Pearson: r=-0.60, p<2.2e-16), FHCRC (Pearson: r=-0.72, p<2.2e-16), and LuCaP PDXs (Pearson: r=-0.88, p<2.2e-16) datasets.

Supplemental Figure 4

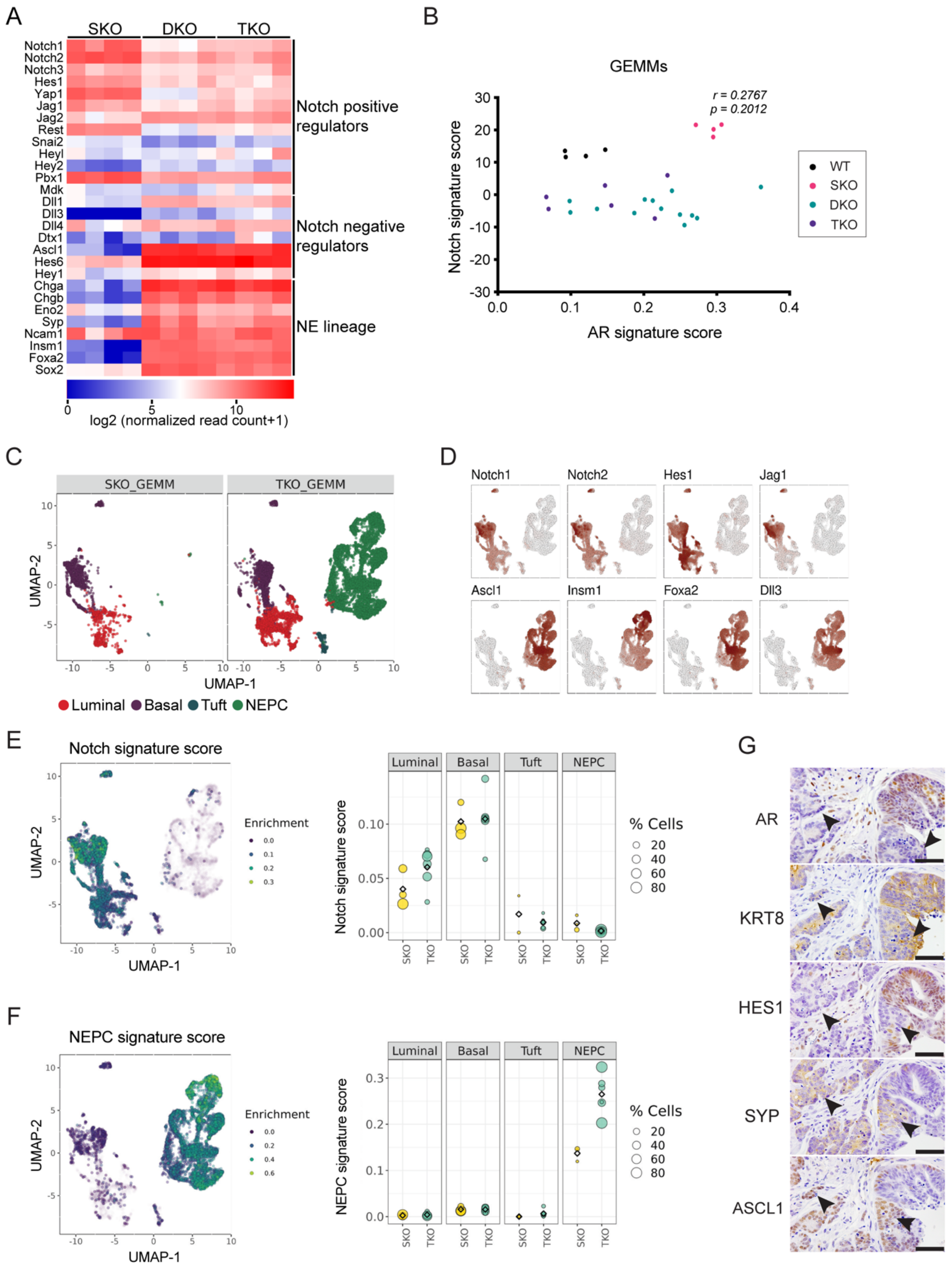

Supplemental Figure 4. Notch signaling in GEMMs with prostate cancer. (A) End-stage tumors from SKO, DKO, and TKO mice (n=4 mice per genotype) were dissected and gene expression profiled by bulk RNA-seq. Log2 (normalized read count +1) data for the indicated Notch signaling genes and neuroendocrine (NE) marker genes were plotted as a color coded heatmap. **(B)** Spearman correlation analysis did not detect a significant correlation of Notch signaling and AR signature scores in GEMMs. **(C)** Prostate tissues from SKO (n=3) and TKO (n=5) mice spanning ages from early to late prostate cancer progression were analyzed by scRNA-seq and presented as a UMAP with transcriptional clusters color coded based on their expression of lineage marker genes. **(D)** Expression levels of indicated genes are shown in each cluster. **(E)** Notch signaling score is enriched in the luminal and basal clusters, but not in NEPC. **(F)** NEPC score enriches in the NEPC cluster, but not in the luminal, basal, and tuft clusters. **(G)** Images of representative tissue sections from TKO mice at the early age stained by H&E or indicated proteins are shown. Arrows identify early NEPC lesions developing in these mice. Scale bar is 50 µm.

Supplemental Figure 5

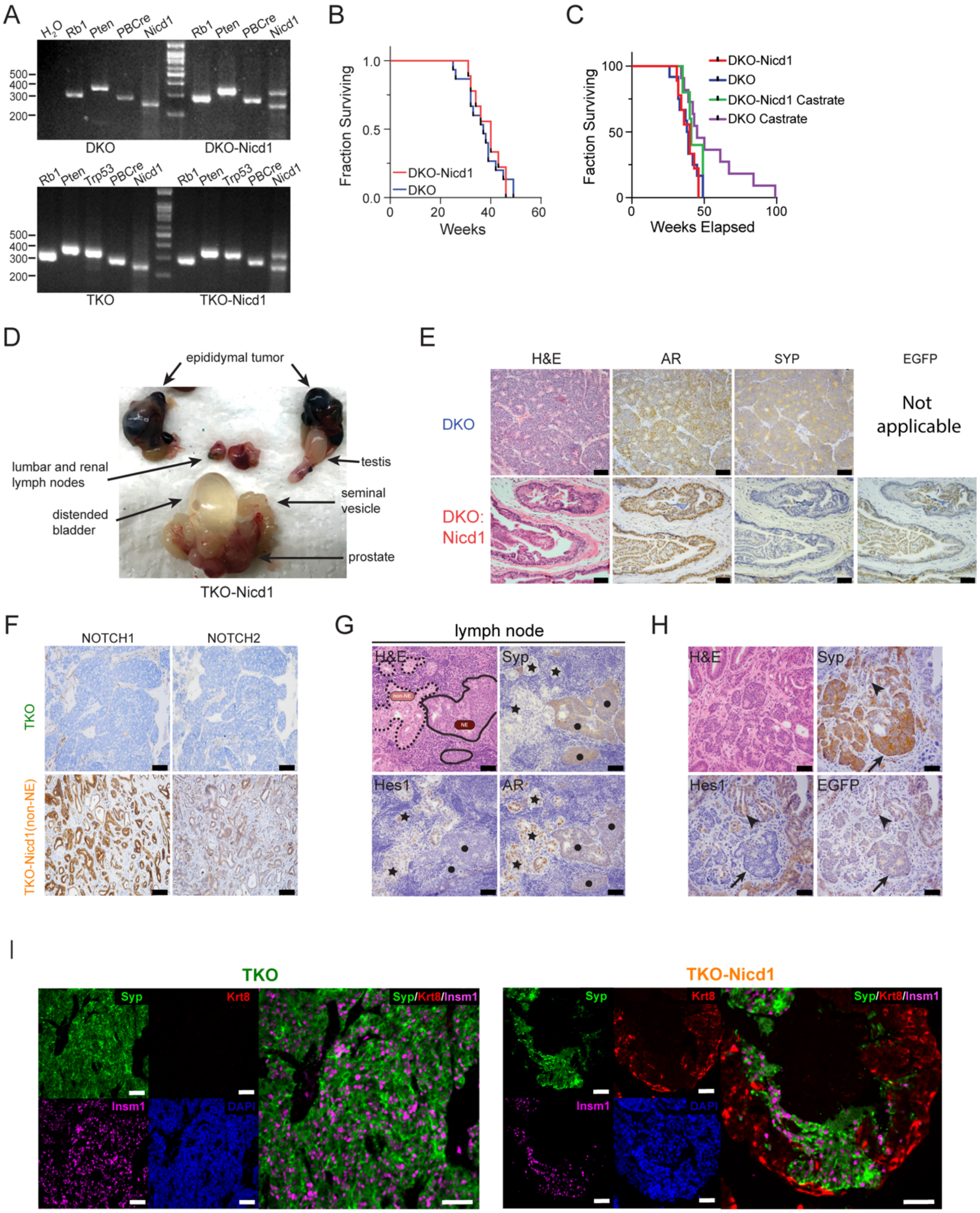

Supplemental Figure 5. Characterization of DKO/DKO-*Nicd1* and TKO/TKO-*Nicd1* GEMMs. (A) Genotyping of indicated mice was performed by PCR amplification of genomic DNA extracted from the mouse tail. Primers used amplify fragments diagnostic for the *Rb1* floxed allele (295 bp, wild type 247 bp), *Pten* floxed allele (328 bp, wild type 156 bp), *Trp53* floxed allele (316 bp, wild type 169 bp), PBCre4 transgene (296 bp), or *Nicd1* transgene (300 bp, wild type 241 bp). **(B)** The survival curve of DKO and DKO-*Nicd1* mice is shown. Median survival is 39 weeks for DKO and 40 weeks for DKO-*Nicd1* mice. It is not significantly different. **(C)** Survival of intact and castrated DKO and DKO-*Nicd1* mice. **(D)** Image showing gross phenotype of epididymal tumor, enlarged lymph nodes, distended bladder, and prostate tumor in TKO-*Nicd1* mice. **(E)** Histological and immunostaining analysis revealed AR, SYP and EGFP levels in primary DKO and DKO-*Nicd1* prostate tumors. Scale bar is 50 μm. **(F)** Primary tumor sections of TKO and TKO-*Nicd1* models were stained with NOTCH1 and NOTCH2. Scale bar is 50 μm. **(G)** Regional lymph node tissue from TKO-*Nicd1* mice. A representative image is shown for H&E and SYP, HES1 as a measure of Notch signaling activity, and AR to indicate a mix of AR-positive adenocarcinoma (★) and SYP-positive neuroendocrine phenotypes (·) that correlated with HES1 expression. Scale bar is 50 μm. **(H)** Low EGFP transgene expression associates with the NE phenotype and reflects the mutual exclusivity of HES1 and SYP expression in TKO-*Nicd1* tumors. **(I)** Immunofluorescent staining of TKO and TKO-*Nicd1* tumors for SYP (green), KRT8 (red), INSM1 (magenta), and DNA (blue). The NE lineage is labeled as SYP+/INSM1+/KRT8-; and the luminal lineage is labeled as SYP-/INSM1-/KRT8+. Scale bar is 50 μm.

Supplemental Figure 6

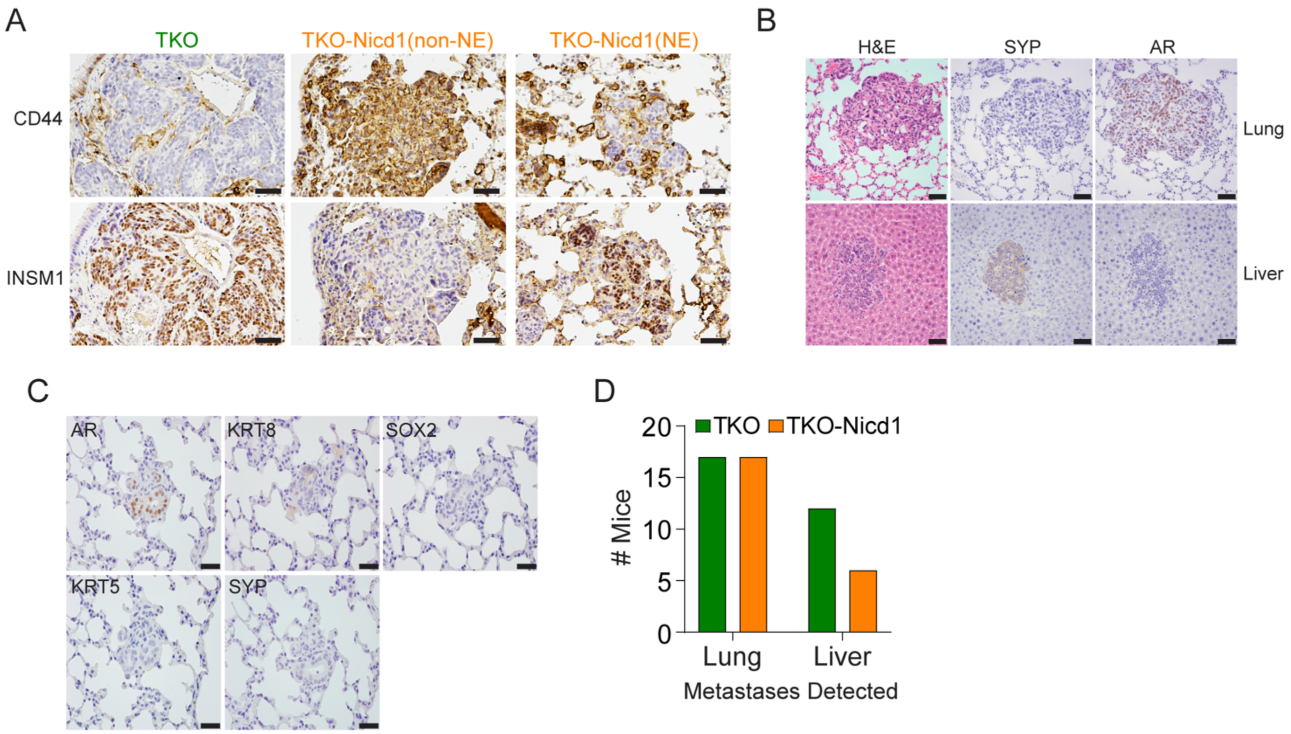

Supplemental Figure 6. Characterizations of metastasis in TKO and TKO-*Nicd1* GEMMs. (A) Lung tissue sections from TKO and TKO-*Nicd1* mice stained with CD44 and INSM1 to indicate stem cell and NE phenotypes. Scale bar is 50μm. **(B)** Representative images of lung and liver tissue sections used to detect distant prostate cancer metastasis in TKO-*Nicd1* mice, stained with AR and SYP to highlight both non-NE (lung) and NE (liver) metastatic foci. Scale bar is 50μm. **(C)** Lung tissue sections of TKO-*Nicd1* stained with indicated protein to indicate non-NE lesions. Scale bar is 50μm. **(D)** The fraction of mice examined (n=17 for both TKO and TKO-*Nicd1*) exhibiting liver and lung metastasis is shown.

Supplemental Figure 7

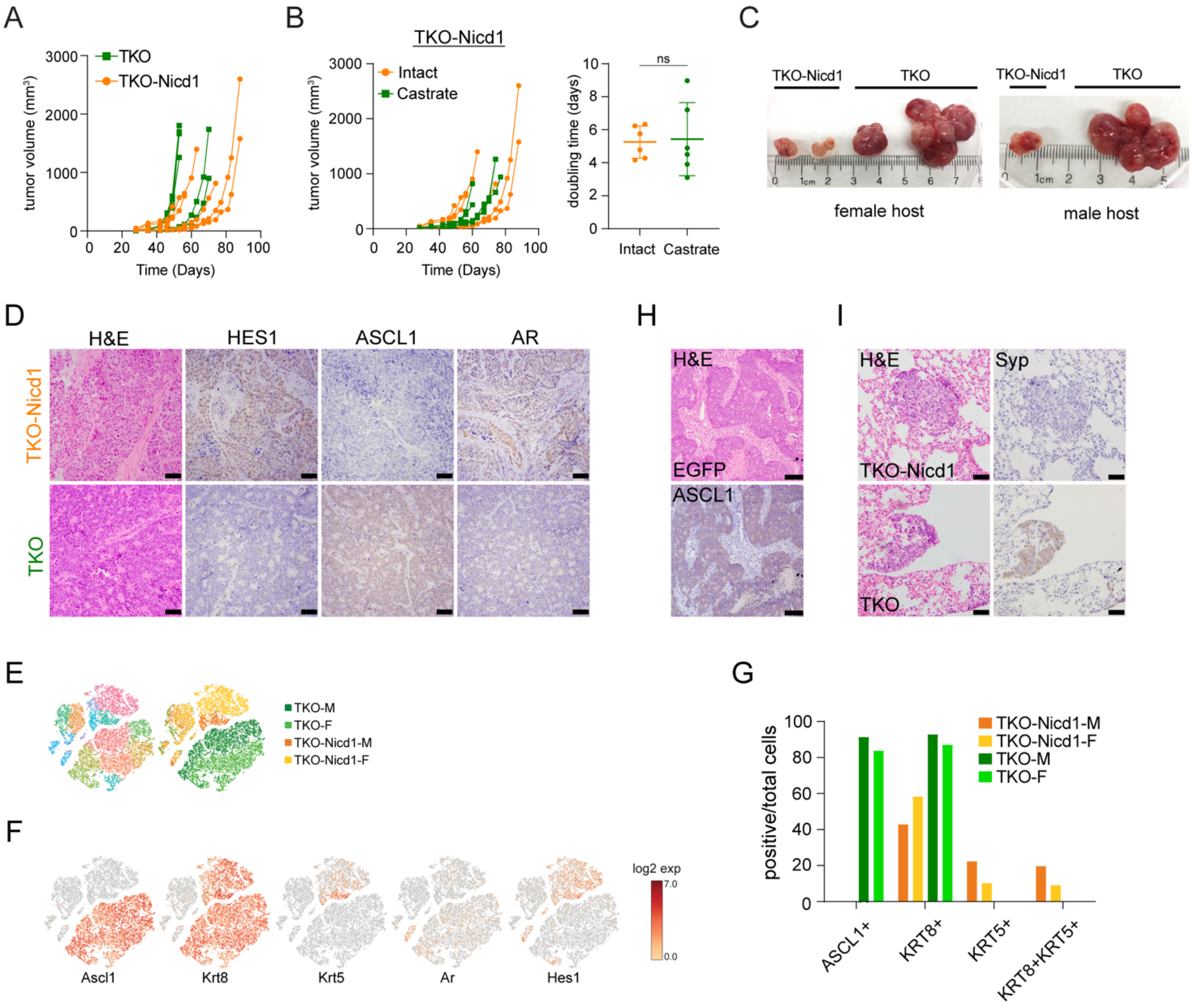

Supplemental Figure 7. Phenotypes of TKO and TKO-*Nicd1* organoid derived tumors *in vivo*. (A) TKO-*Nicd1* organoids (selected for high EGFP) or TKO organoids were transplanted subcutaneously in SCID mice (n=6 for each genotype), and tumor volumes measured over time. Each line represents tumor growth in an individual mouse. **(B)** Tumor volumes of TKO-*Nicd1* organoids were measured over time. Non-linear regression was used to calculate the average tumor growth rate for each condition. There is no significant difference between intact and castration. **(C)** The indicated organoids were transplanted subcutaneously into either male of female mice, allowed to grow for 8 weeks, then the mice euthanized and tumors dissected. **(D)** Representative images of tissue sections from transplant tumors are shown, stained with H&E or antibodies directed against the indicated antigens. Scale bar is 50 µm. **(E)** scRNA-seq of transplanted tumors shown in **(C).** The left panel shows graphical clustering of all analyzed cells (17,421) while the right panel shows cells color coded by genotype and mouse host (TKO-*Nicd1*-M=2,949 cells; TKO-*Nicd1*-F=4,230; TKO-M=5,554; TKO-F=4,688). **(F)** The cluster diagrams in **(E)** were color coded for log2 expression of the indicated lineage marker genes. **(G)** The graph shows the proportion of cells analyzed positive for the indicated marker genes for each genotype and mouse host. **(H)** TKO-*Nicd1* organoids with low EGFP expression were selected and transplanted subcutaneously into male mice. Tumors were dissected at end point and histology analyzed. Representative images of tissue sections from transplant tumors are shown, stained with H&E or antibodies directed against the neuroendocrine marker ASCL1. Scale bar is 100 µm. **(I)** Lung tissue was dissected from mice with transplanted with the indicated organoids, and lung tissue sections analyzed for evidence of metastasis. Representative lung tissue sections containing metastatic lesions are shown, stained for H&E or immunostained for the neuroendocrine marker SYP. Scale bar is 50 µm.

Supplemental Figure 8

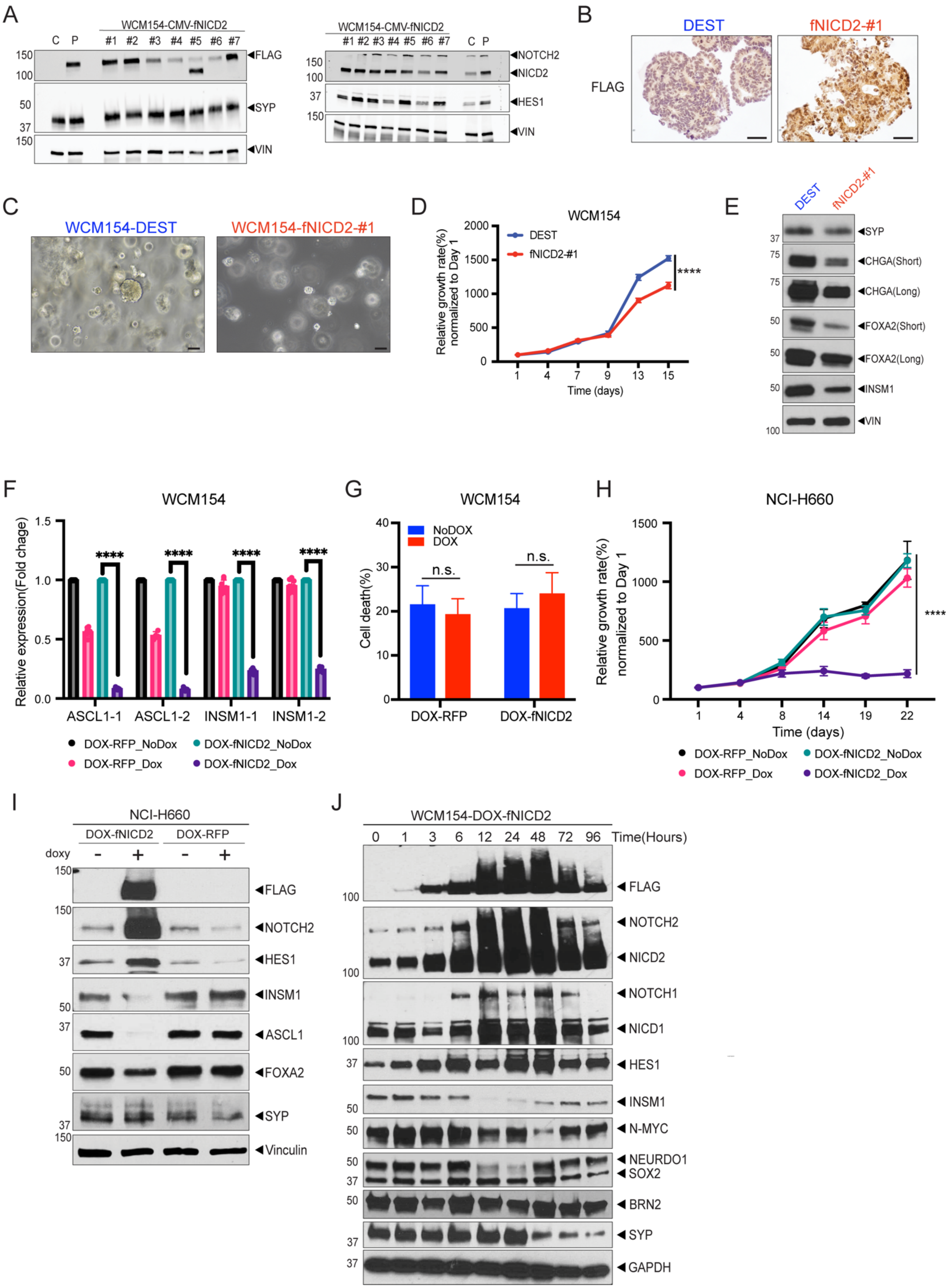

Supplemental Figure 8. NOTCH2 expression in human NEPC organoids and cell line. (A) A CMV-fNICD2 expressing plasmid was introduced in WCM154 organoids and stable clones were selected by puromycin. Pooled infected WCM154 organoids (P) were then plated in multi-well plates as a single cell to identify pure fNICD2-expressing clones. **(B)** FLAG expression in WCM154-DEST and fNICD2-#1 organoids. **(C)** Brightfield images of WCM154-DEST and fNICD2-#1 organoids corresponding to Fig 3A. Scale bar is 100 μm. **(D)** Relative organoid growth revealed significantly decreased in fNICD2-#1. Data represent mean ± SD. Two-way ANOVA was performed (*****p<0.0001*). **(E)** Immunoblot analysis indicated downregulated neuroendocrine markers, CHGA, FOXA2, INSM1, in fNICD2-#1 organoids. **(F)** WCM154-DOX-RFP and -fNICD2 organoids were treated with doxycycline for 24 hours. *ASCL1* and *INSM1* mRNA levels were measured by QPCR with two independent pairs of primers in WCM154-DOX-fNICD2 organoids. Two-tailed t test was performed to determine statistical significance between NoDox and Dox (*****p<0.0001*). **(G)** Cell death was measured by trypan blue staining. Data was collected form two independent experiments with multiple technical replicates. Data represent mean ± SD. Two-tailed t test was performed to determine statistical significance between NoDox and Dox. *n.s.* : not significant. **(H)** H660-DOX-RFP and -fNICD2 cells were treated with doxycycline. Relative growth rate was measured at indicated timepoints. Data represent mean ± SD. Two-way ANOVA was performed (*****p<0.0001*). **(I)** fNICD2 expression was induced in H660-DOX-fNICD2 cells resulting in downregulation of SYP and INSM1. **(J)** Expression of Notch signaling (NOTCH1, NOTCH2, HES1), and neuroendocrine genes (INSM1, N-MYC, NEUROD1, SOX2, BRN2, SYP) at indicated timepoints post-doxycycline treatment in WCM154-DOX-fNICD2 organoids.

Supplemental Figure 9

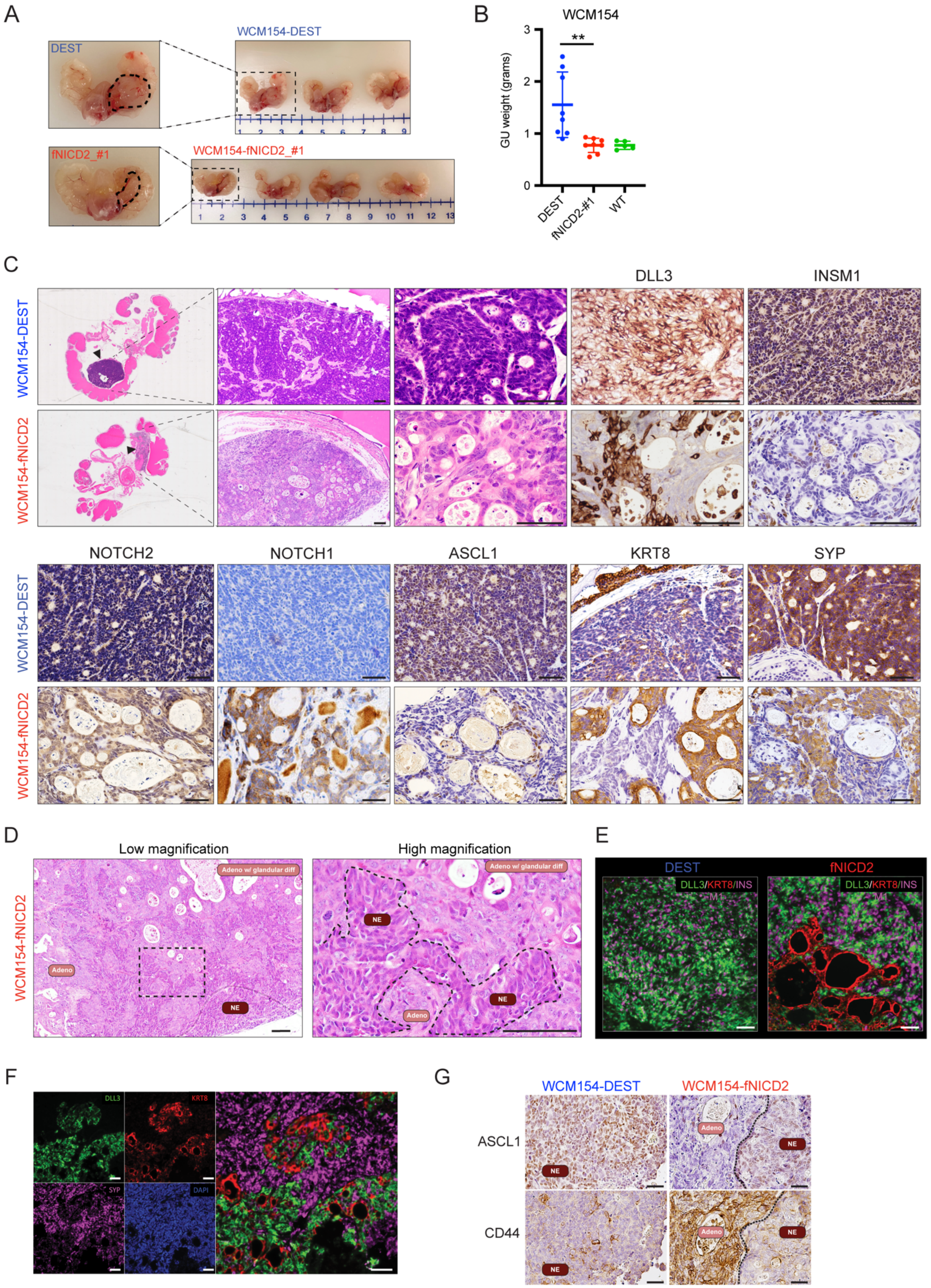

Supplemental Figure 9. *In vivo* tumor development of WCM154-fNICD2-#1 organoids. (A) Representative images of mouse genitourinary where WCM154-DEST (control) and fNICD2-#1 organoids were injected into one side of anterior prostate. Images were taken at 4 months after injection. **(B)** The end-stage GU weight of WCM154-DEST (n = 8) and fNICD2-#1 mice (n = 8). Two-tailed t test was performed (***p* < 0.01). **(C)** WCM154-DEST and fNICD2-#1 orthotopical tumors were stained by H&E and indicated antibodies for Notch signaling, luminal and NE lineages. Scale bar is 100μm. **(D)** fNICD2 tumor displays both NE and adenocarcinoma (Adeno) components. Scale bar is 100μm. **(E)** WCM154-DEST and fNICD2-#1 tumors were co-stained with DLL3(green), KRT8(red), and INSM1(magenta). Scale bar is 50μm. **(F)** fNICD2-#1 tumors were co-stained with DLL3(green), KRT8(red), and SYP(magenta), and DAPI (blue). Scale bar is 50μm. **(G)** WCM154-DEST and fNICD2-#1 tumors were stained for ASCL1 and CD44. Scale bar is 50μm.

Supplemental Figure 10

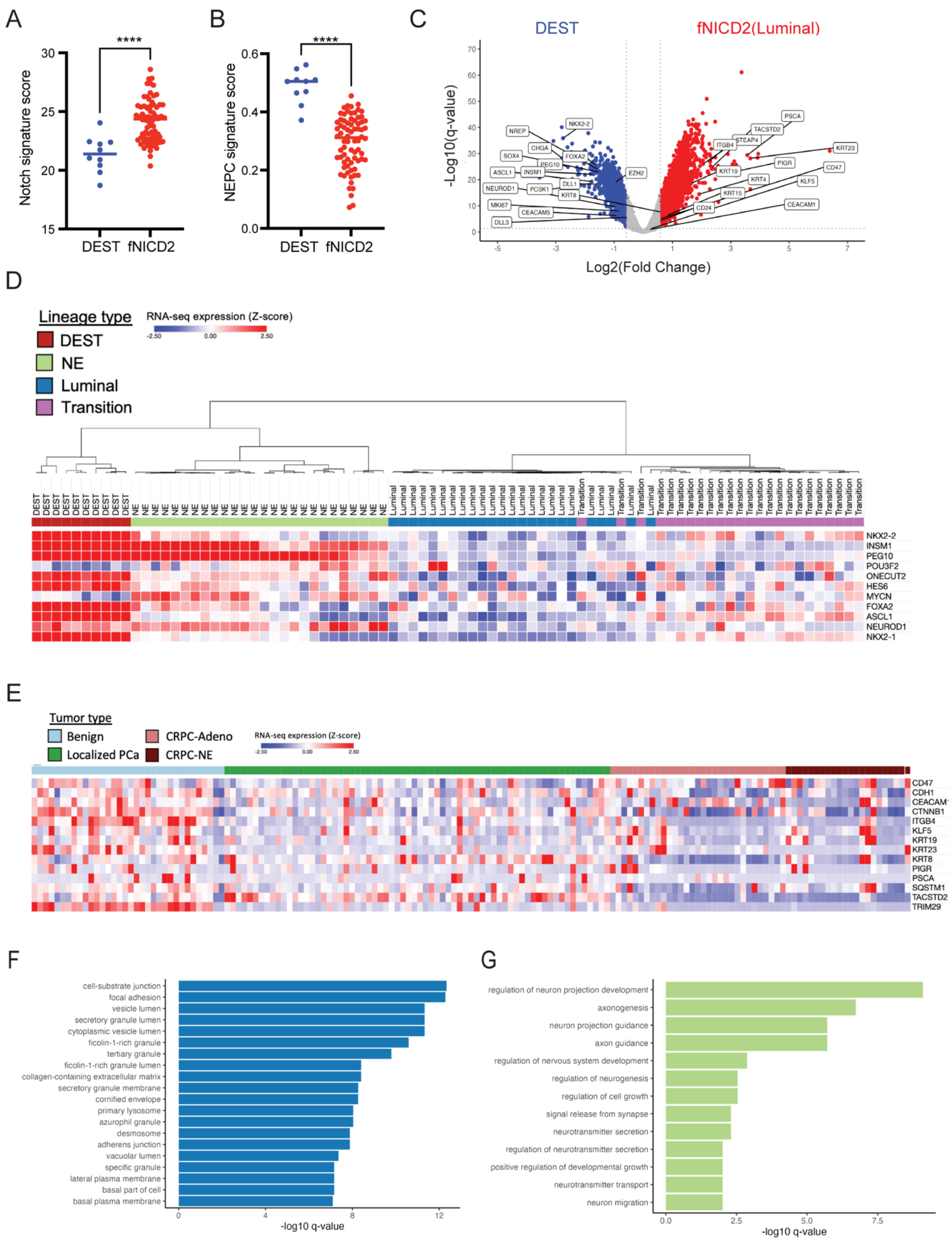

Supplemental Figure 10. Whole transcriptome digital spatial profiling on WCM154-DEST and fNICD2-#1 tumor lineages. (A) Notch signaling and **(B)** NEPC signature scores in WCM154-DEST and fNICD2-#1 tumors. **(C)** Volcano plot indicated differential gene expression of WCM154-DEST and Luminal lineages in fNICD2-#1 tumors. **(D)** Expression of NE lineage transcription factors in WCM154-DEST tumor and NE, mixed/transition, and luminal lineages of fNICD2-#1. **(E)** Expression of Luminal enriched genes in benign, localized prostate cancer (PCa), CRPC-Adeno, and CRPC-NE in the Beltran dataset(3, 4). **(F)** GO analysis of enriched biological processes in the luminal lineage of fNICD2-#1. **(G)** GO analysis of enriched biological processes in the NE lineage of fNICD2-#1.

Supplemental Figure 11

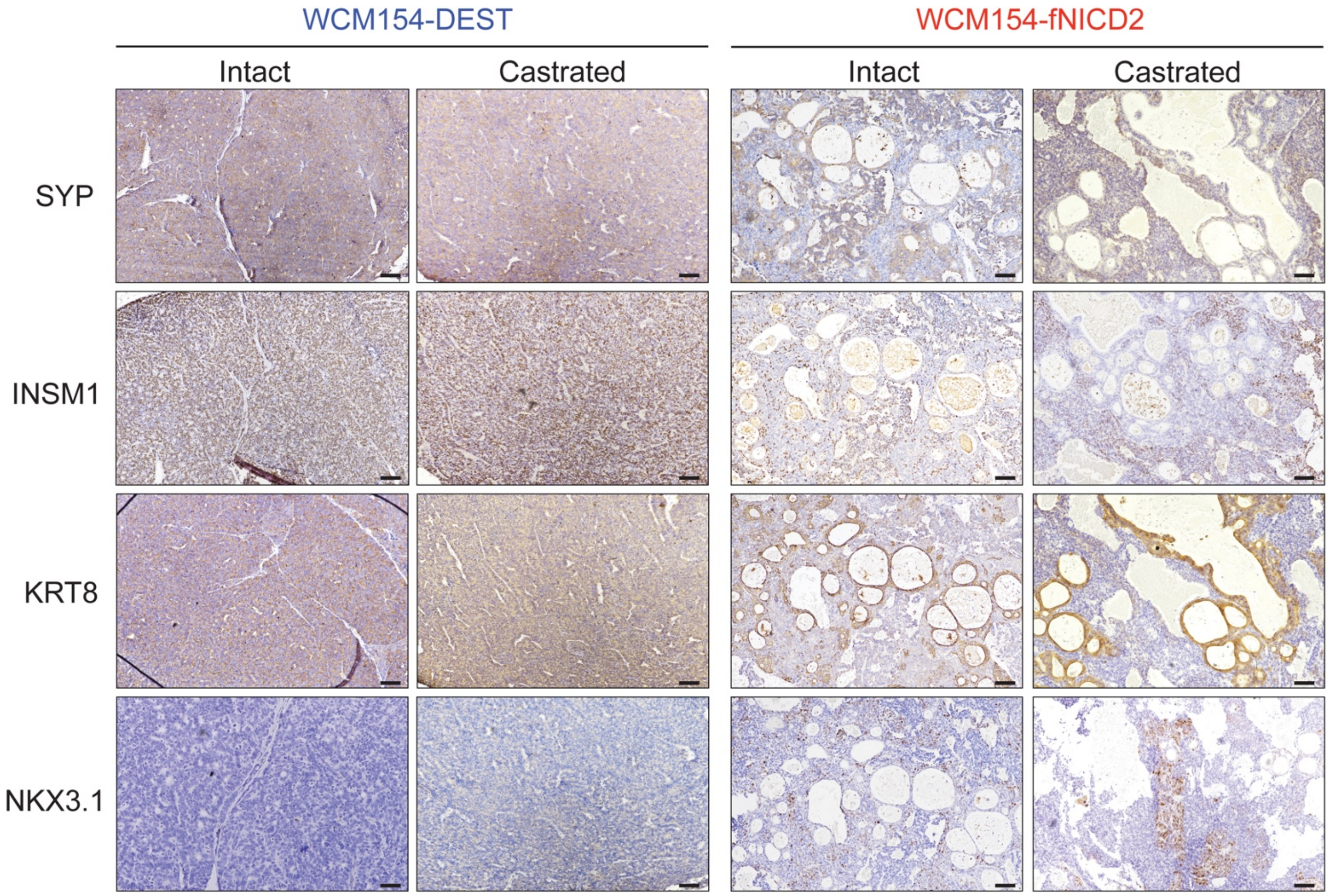

Supplemental Figure 11. Histology of intact and castrated WCM154-DEST and fNICD2 tumors. Both intact and castrated WCM154-DEST tumors express SYP, INSM1, low KRT8 but not NXK3.1. In contrast, intact and castrated fNICD2-#1 tumors display reduced levels of SYP and INSM1, increased KRT8 and NKX3.1. Scale bar is 200 μm.

Supplemental Figure 12

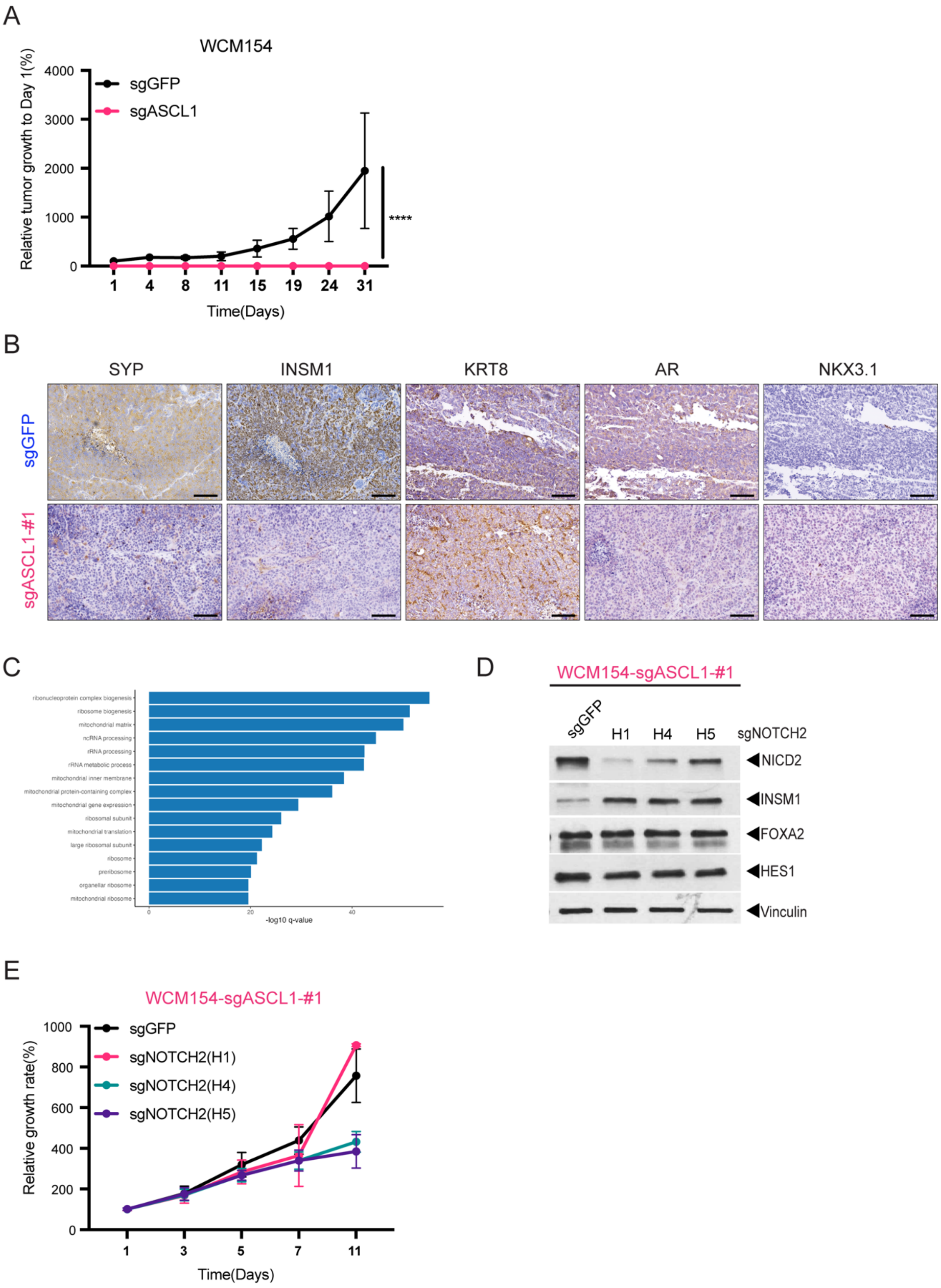

Supplemental Figure 12. Lineage characterizations of WCM154-sg*ASCL1* tumor. (A) WCM154-sg*GFP* and -sg*ASCL1*-#1 organoids were subcutaneously implanted in NSG mice (n=3/group). The study was terminated when sg*GFP* tumors reached the endpoint and no tumors were formed in the sg*ASCL1* group. Data represent mean ± SD. Two-way ANOVA was performed (****$p < 0.0001$). **(B)** WCM154-sg*GFP*(control) and WCM154-sg*ASCL1* tumors were stained with NE markers (SYP, INSM1) and luminal markers (KRT8, AR, NKX3.1). Scale bar is 100 μm. **(C)** The GO analysis shows upregulated biological processes in *ASCL1* knockout tumors. **(D)** Three independent sgRNAs against *NOTCH2* were used. Expression of NICD2, INSM1, FOXA2, and HES1 in NOTCH2 knockout WCM154-sg*ASCL1* organoids. Vinculin serves as a loading control. **(E)** Relative organoid growth of *NOTCH2* knockout in WCM154-sg*ASCL1*. Data represent mean ± SD.

Supplementary Fig 13

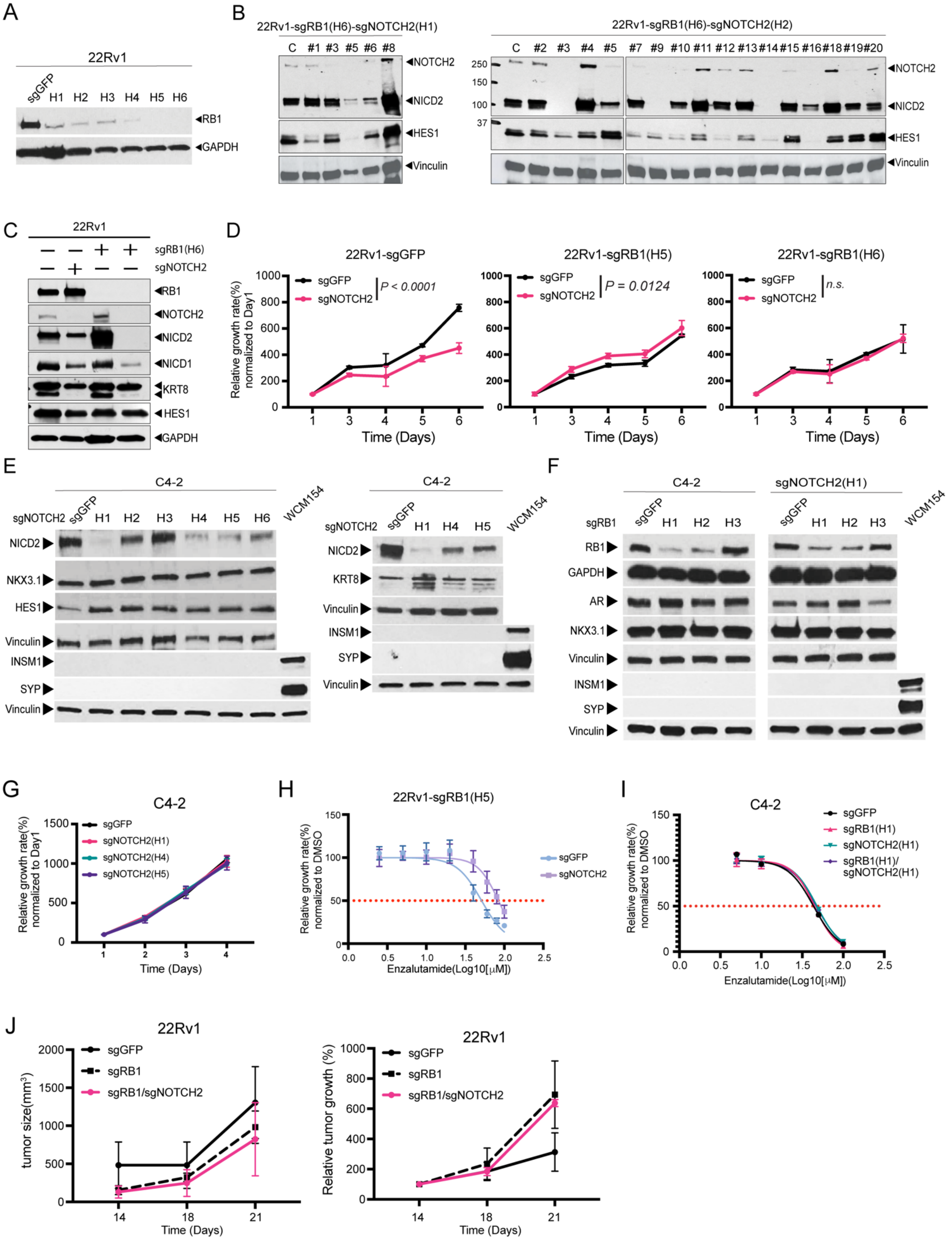

Supplemental Figure 13. Deletion NOTCH2 in *RB1*-loss 22Rv1 cells. (A) Six independent sgRNAs were used to target *RB1* in 22Rv1 cells. Clone H5 & H6 were selected for the study. **(B)** *NOTCH2* was deleted using CRISPR-Cas9 using two different sgRNAs (H1 and H2). Single cell selection was conducted to identify knockout clones by evaluating NICD2 and HES1 expression. Vinculin serves as a loading control. **(C)** Expression of RB1 and NOTCH2 in selected 22Rv1 knockout clones. GAPDH serves as a loading control. **(D)** Cell growth of *NOTCH2* knockout combing with *RB1* loss. Data represent mean ± SD. Two-way ANOVA was used to determine statistical significance. **(E)** Six independent sgRNAs were tested to target *NOTCH2* in C4-2 cells. Expression of NICD2, NKX3.1, HES1, KRT8, INSM1, and SYP in selected C4-2 knockout clones. WCM154 serves as a positive control for INSM1 and SYP. Vinculin serves as a loading control. **(F)** Three independent sgRNAs were used to target *RB1* in C4-2 and C4-2-sg*NOTCH2*(H1) cells. **(G)** Relative cell growth of C4-2 sg*NOTCH2* were measured using CellTiter-Glo at indicated timepoints. **(H)** 22Rv1-sg*RB1*(H5) cells with/out *NOTCH2* loss were treated with DMSO or Enzalutamide with indicated concentrations for 6 days. Relative cell growth was measured by CellTiter-Glo on Day 6 and normalized to DMSO. IC50 was determined using GraphPad. 22Rv1-sgGFP: 51.1μM; 22Rv1-sg*RB1*/sg*NOTCH2*: 84.1μM. **(I)** C4-2 cells with/out *RB1 and NOTCH2* loss were treated with DMSO or Enzalutamide with indicated concentrations for 6 days. Relative cell growth was measured by CellTiter-Glo on Day 6 and normalized to DMSO. **(J)** 22Rv1 cells with/out *RB1* and *NOTCH2* loss were subcutaneously injected into castrated NSG mice (n=3/group). Tumor size was measured at indicated timepoints. Relative growth was calculated by normalizing to initial tumor size. Data represent mean ± SD.

Supplemental Figure 14

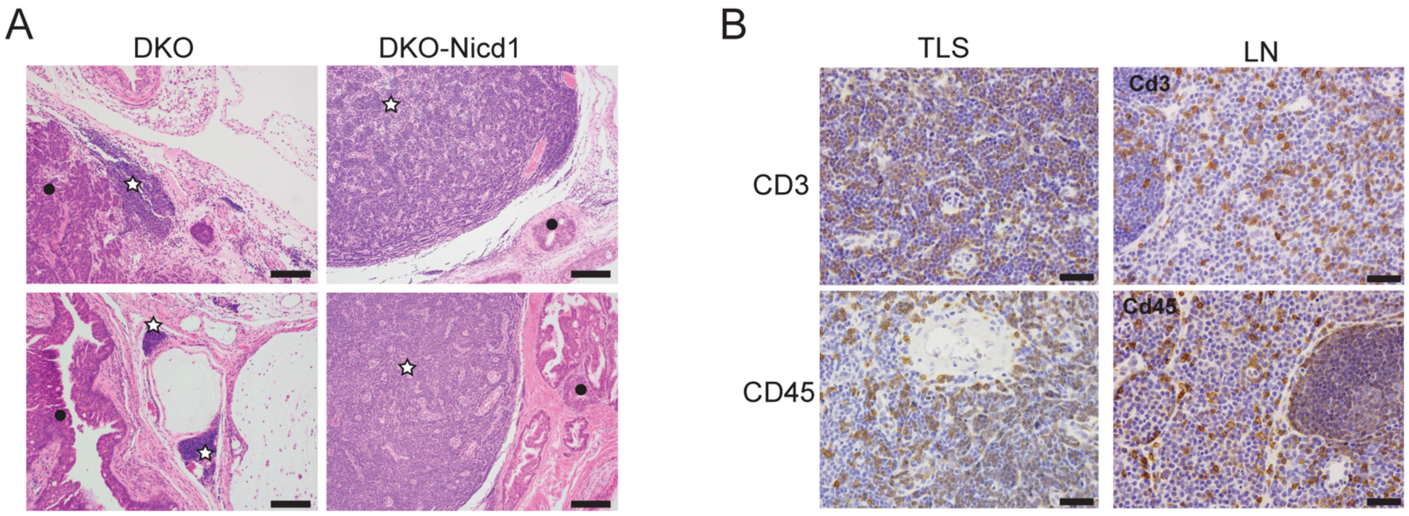

Supplemental Figure 14. Tumor microenvironment in NEPC GEMMs. (A) Prostate tissue was dissected from DKO and DKO-*Ncd1* mice, fixed, sectioned, and H&E stained for histopathological analysis. Representative examples of DKO-*Nicd1* mice developing TLS (★) within the prostate (·) are shown along with DKO control mice where TLS are less frequent and smaller. Scale bar is 100 µm. **(B)** Prostate TLS tissue and regional lymph nodes sections were immunostained with the T cell marker CD3 and the lymphocyte marker CD45. Representative images are shown. Scale bar is 20 µm.

Supplemental Figure 15

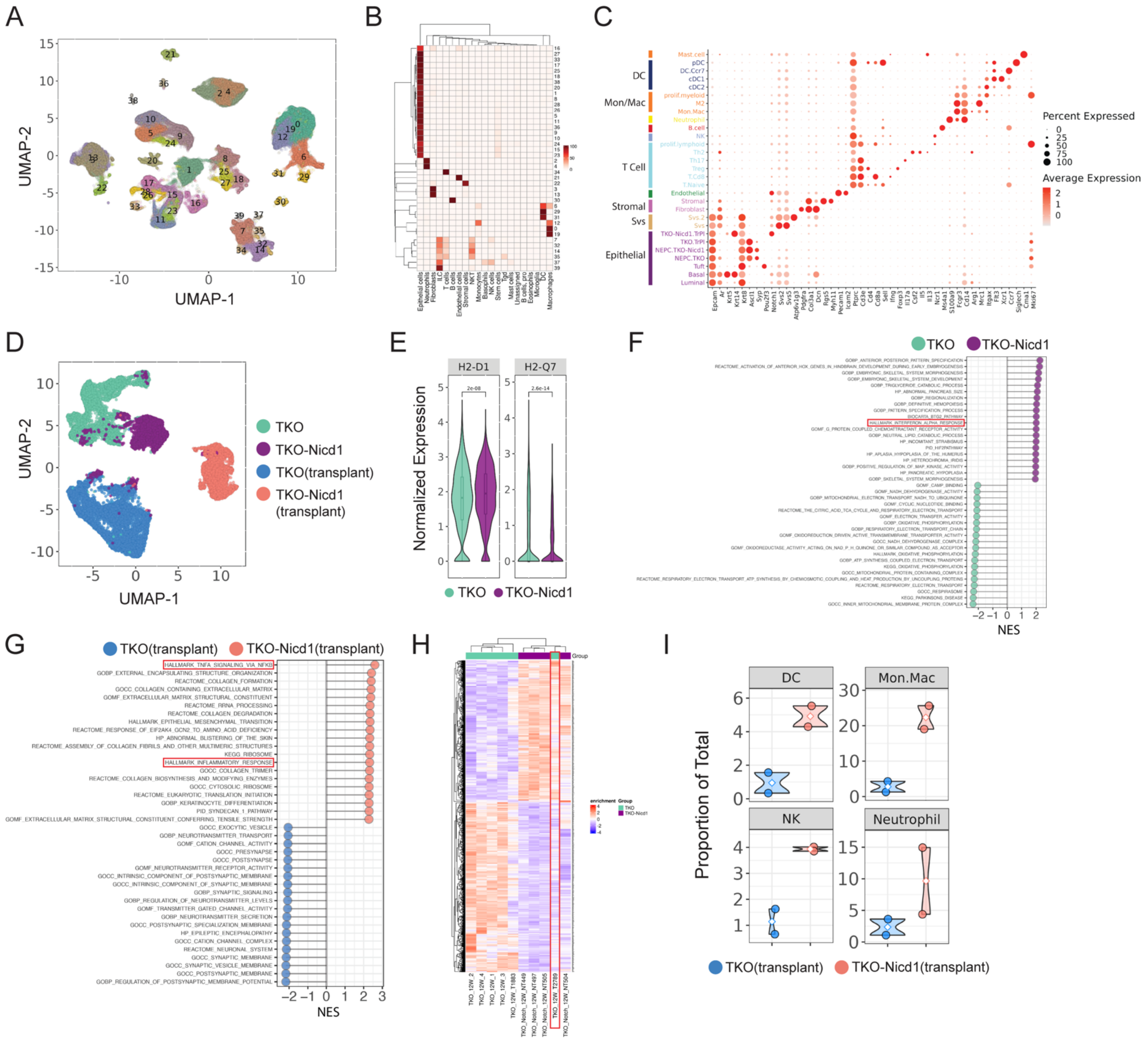

Supplemental Figure 15. Notch signaling alters the tumor immune microenvironment in TKO and TKO-*Nicd1* tissue and transplant tumors. (A) UMAP visualization of cells transcriptionally clustered using a partitioned KNN graph-based approach (Louvain). **(B)** Heatmap depicting the proportion of cells within each cluster annotated to major cell lineages as defined by the ImmGen database using a supervised classification approach (SingleR). **(C)** The graph shows the expression of lineage markers in individual cell types. Cell types are color coded including epithelial tumor cells, stroma, and immune cells. Expression levels are indicated by the size and intensity. **(D)** The UMAP displays malignant cells from the indicated samples transcriptionally clustered separately, indicating that transcriptional patterns within the cancer cells are mostly distinct. **(E)** Normalized expression of interferon/inflammatory (*Ifitm1, Ckap4*) and MHC genes (B2m, H2-K1, H2-D1, H2-Q7) in neoplastic cells from TKO and TKO-*Nicd1* GEMMs was determined by scRNA-seq (Supplemental Figure 15D). Wilcox tests were used to assess differences between genotypes with *p* values shown. **(F)** GSEA analysis comparing prostate tissue from TKO and TKO-*Nicd1* GEMMs. Interferon-α response (highlighted) is enriched in TKO-*Nicd1*. **(G)** GSEA analysis comparing TKO and TKO-*Nicd1* transplant tumors. TNF-α signaling and inflammatory response pathways (highlighted) are enriched in TKO-*Nicd1* tumors. **(H)** Heatmap of differential gene expression indicates an outlier TKO tumor clustered with the TKO-*Nicd1* tumors. **(I)** Proportion of immune cells is compared in TKO and TKO-*Nicd1* transplant tumors.

Supplemental Figure 16

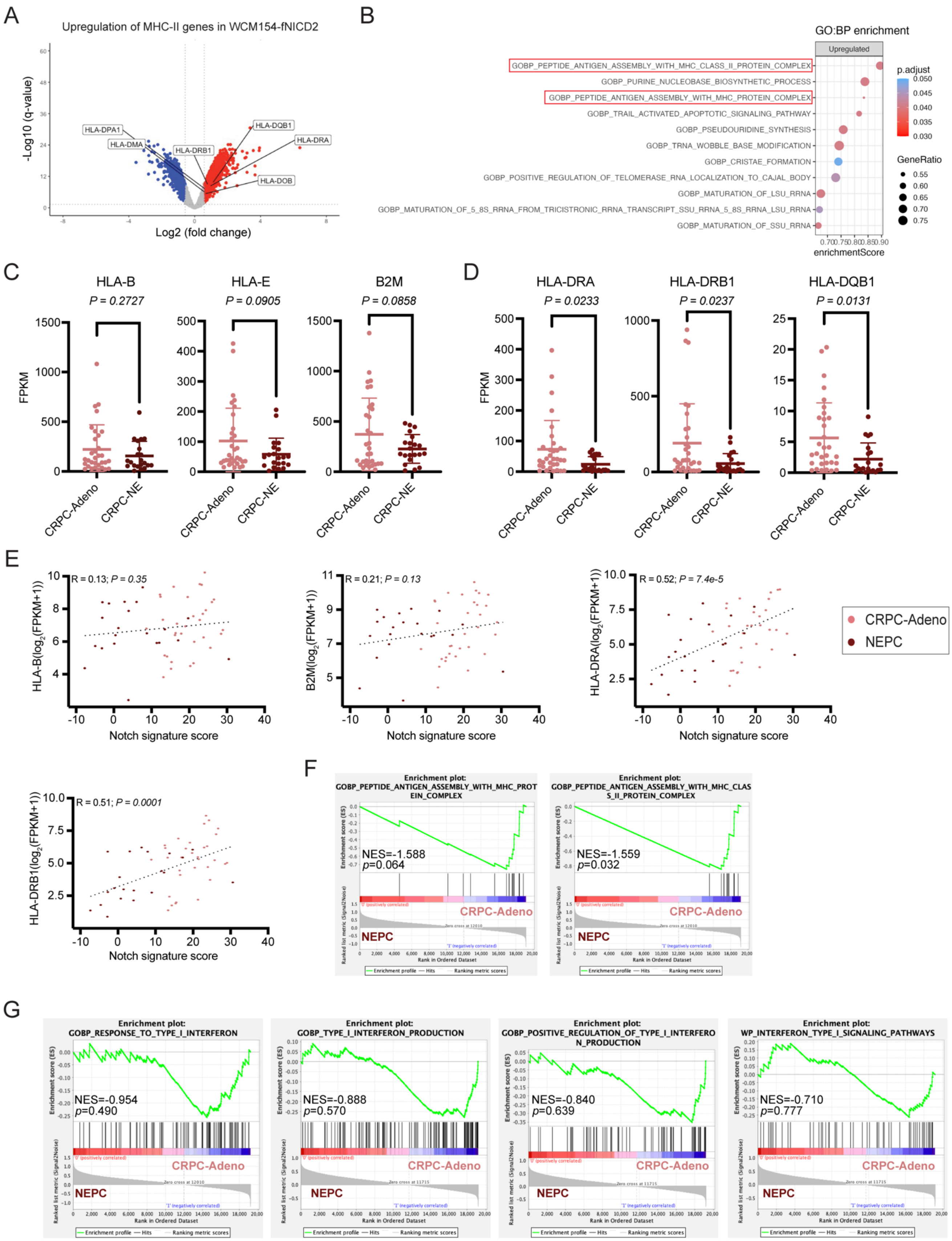

Supplemental Figure 16. Notch signaling increases MHC-I/II in human NEPC models (A) Volcano plot comparing gene expression in fNICD2 and control transplant tumors. Increased expression of MHC-II genes was labeled. **(B)** GO analysis indicates *ASCL1* knockout tumors exhibit elevated expression of genes related to MHC and antigen presentation. **(C**) MHC-I and **(D)** MHC-II expression in the Beltran dataset. Two-tailed T test was performed to determine the statistical significance. **(E)** Correlation of MHC-I and -II with Notch signature score. Pearson correlation analysis was performed, and the statistical significance is shown on the graph. **(F)** GSEA analysis indicates the enrichment of MHC GO in CRPC-Adeno in the Beltran dataset. Enrichment score and stats are shown on the graph. **(G)** GSEA analysis indicates the enrichment of type I interferon signaling in CRPC-Adeno in the Beltran dataset. Enrichment score and stat are shown on the graph.

Supplemental Figure 17

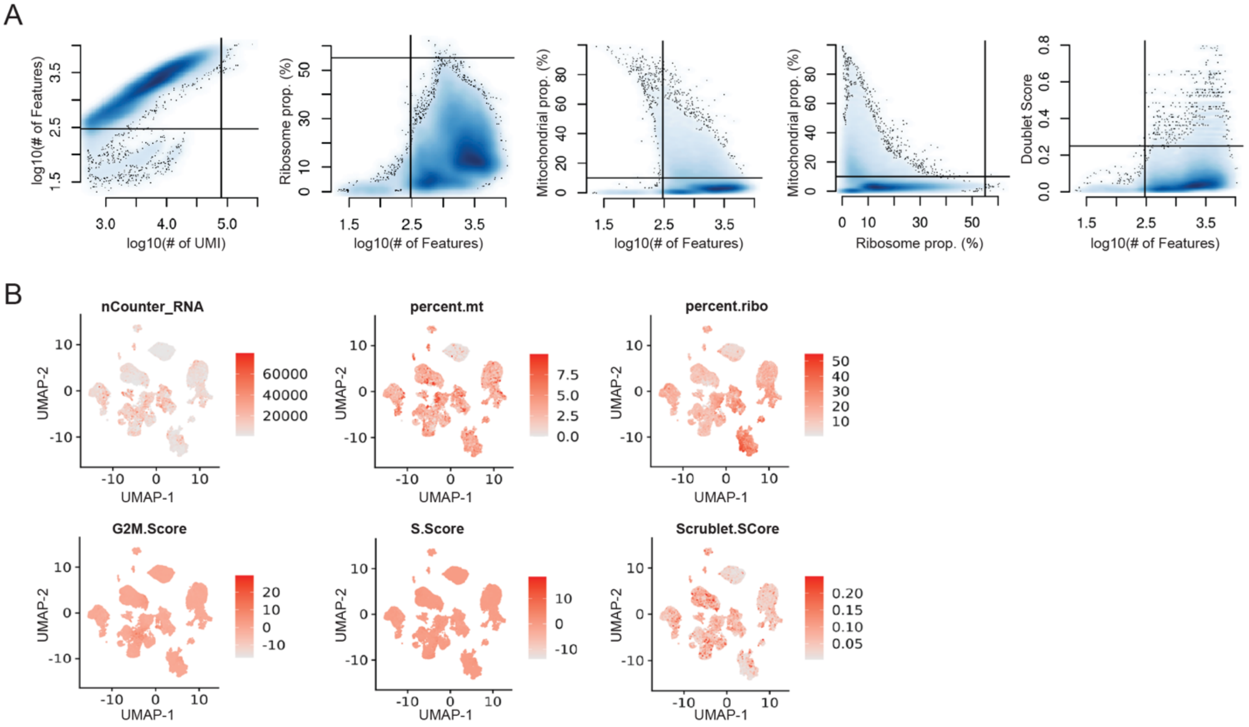

Supplemental Figure 17. Quality control of scRNA-seq related to Fig 8. (A) scRNA-seq quality control metrics (mitochondrial expression < 10%, features > 300, UMI < 80,000, ribosomal expression < 55%, Doublet Score < 0.25) used for filtering high quality cells for downstream analysis are shown. **(B)** Distribution of cell quality criteria across the transcriptional clusters is shown.

Supplemental Methods

Single cell RNA-seq raw data processing, quality control, and subsequent analyses

Raw sequence data demultiplexing, barcode processing, alignment (mm10) and filtering for true cells were performed using the Cell Ranger Single-Cell Software Suite (v6.0.0), yielding 92,973 putative cells for analysis. Subsequent filtering for high quality cells, and downstream analyses were performed using Seurat (v4) (5) (Supplemental Figure 17A-B). Genes expressed in less than 3 cells and cells that express less than 300 genes were excluded from further analyses. Additional filtering of cells was determined based on the overall distributions of total RNA counts (< 80,000) and the proportion of mitochondrial genes (< 10%) detected to eliminate potential doublets and dying cells, respectively (6). Additional detection of doublets was performed using Scrublet (6). Thresholding for doublet detection was set based on total distribution of doublet scores (doublet threshold = 0.25). Quantification of mitochondrial and ribosomal gene expression was calculated using the PercentageFeatureSet function, using gene sets compiled from the HUGO Gene Nomenclature Committee database. Ultimately, 18,076 cells were removed (19.44% of total cells) after quality control assessment, and 74,897 high quality cells were included in downstream analyses. Normalization and variance stabilization were conducted using regularized negative binomial regression (sctransform) implemented with Seurat. Principle component analysis (PCA) was performed on normalized data and optimal dimensionality of the dataset was decided by examination of the Elbow plot, as the total number of PCs where gain in cumulative variation explained was greater than 0.1% (PCs = 42). The FindNeighbors function was utilized that implements a graph based nearest neighbor clustering approach, and the FindClusters function was used to identify final cell clusters using a resolution of 0.08 (Supplemental Figure 15A). UMAP was applied for non-linear dimensional reduction to obtain a low dimensional representation of cellular states. Single-cell gene set enrichment was performed using UCell (7). Smoothing of gene expression across cells for visualization purposes was performed using MAGIC (8), with parameters knn = 15, t = 3. Initial unbiased cell type annotation was performed using SingleR (9) against the ImmGen database to infer major cell lineage (Supplemental Fig 15B). Higher resolution cell annotation

was performed using curated gene sets for various established immune and epithelial cell states (Figure 8A and Supplemental Figure 15C). Tumor subsets were filtered from total cell populations using final cell annotations after careful pruning for ambiguously annotated cells and re-analyzed as described above. Differential expression (DE) between tumor populations amongst TKO and TKO-*Nicd1* counterparts was determined using a pseudobulk based approach. Gene counts were aggregated across cells per sample (TKO n = 6; TKO-*Nicd1* n = 4; TKO_TrPl n = 2; TKO-*Nicd1*_TrPl n = 2), and samples with > $1x10^6$ total counts used for DE analysis. Pseudobulk counts were normalized and DE analysis carried out using DESeq2 (10). Differential expression rank order was used for subsequent gene set enrichment analysis (GSEA) (11), performed using the clusterProfiler package in R. Gene sets queried included the Hallmark, Canonical pathways, and GO Biological Processes Ontology collections available through the Molecular Signatures Database (MSigDB) (12).

FLAG-tagged NICD2 expressing models

The FLAG-tagged *NICD2* (fNICD2) expressing plasmids were constructed using Invitrogen™ Gateway™ cloning. Briefly, FLAG-tagged *NICD2* ORF was excised from the 3XFlagNICD2 plasmid (Addgene plasmid #20184), and subcloned into *NcoI* and *BamHI* sites of pENTR4 entry vector (Addgene plasmid #17423). Subsequently, FLAG-tagged *NICD2* was shuttled to the destination vector pLenti CMV Puro DEST (Addgene plasmid #17452) using Gateway™ LR Clonase™ II Enzyme mix (ThermoFisher Scientific) to generate a pLenti-CMV-fNICD2 expressing plasmid. The pLenti CMV Puro DEST (DEST) plasmid was used as an empty vector control. The pLenti-CMV-fNICD2 plasmid was delivered into WCM154 organoids using lentiviral infection following 1ug/ml puromycin selection for 3-7 days. WCM154-CMV-fNICD2 organoids were then seeded as two-dimension adherent cells in 96-well plates pre-coated with collagen, one cell per well. After 4-6 weeks, wells that only contained single clones were collected and propagated to examine the level of fNICD2. For the DOX inducible system, pInducer20 vector (Addgene plasmid #44012) was used to carry fNICD2 ORF and the cloning method was similar as above.

CRISPR-Cas9 knockout models

CRISPR-Cas9 constructs were generated using all-in-one lentiCRISPR v2 (Addgene plasmid #52961), following the published protocol (13). The sgRNAs sequences are listed in SupplementalTable 6. CRISPR-Cas9 constructs were delivered into target organoids or cells using lentiviral infection, and then infected cells were selected in 1ug/ml puromycin for 3-7 days. Subsequently, CRISPR-Cas9 cells were plated in 96-well plates, one cell per well. After 1-2 months, wells with only one single cell clone were collected and expanded to determine the knockout efficiency. For 22Rv1 and C4-2 *NOTCH2* knockout models, *NOTCH2* crRNA, tracrRNA, and Cas9 (Integrated DNA Technology) were delivered in 22Rv1-sg*GFP* and 22Rv1-sg*RB1* cells by nucleofection following the manufacturer's protocol (Lonza), and then plated in 96-well plates as single cells to establish *NOTCH2* knockout clones. For C4-2 *NOTCH2* knockout models, *NOTCH2* crRNA, tracrRNA, and Cas9 (Integrated DNA Technology) were delivered in C4-2 and C4-2-sg*RB1* cells by nucleofection following the manufacturer's protocol (Lonza).

Bulk RNA-seq

Total RNA was extracted from snap frozen tumors (three per group) using Qiagen RNeasy Plus Universal mini kit following manufacturer's instructions (Qiagen, Hilden, Germany). RNA samples were quantified using Qubit 2.0 Fluorometer (Life Technologies, Carlsbad, CA, USA) and RNA integrity was checked using Agilent TapeStation 4200 (Agilent Technologies, Palo Alto, CA, USA). RNA sequencing libraries were prepared using the NEBNext Ultra II RNA Library Prep for Illumina using manufacturer's instructions (NEB, Ipswich, MA, USA). Briefly, mRNAs were initially enriched with Oligod(T) beads. Enriched mRNAs were fragmented for 15 minutes at 94°C. First strand and second strand cDNA were subsequently synthesized. cDNA fragments were end repaired and adenylated at 3'ends, and universal adapters were ligated to cDNA fragments, followed by index addition and library enrichment by PCR with limited cycles. The sequencing libraries were validated on the Agilent TapeStation (Agilent

Technologies, Palo Alto, CA, USA), and quantified by using Qubit 2.0 Fluorometer (Invitrogen, Carlsbad, CA) as well as by quantitative PCR (KAPA Biosystems, Wilmington, MA, USA). The sequencing libraries were clustered on a lane of a HiSeq flowcell. After clustering, the flowcell was loaded on the Illumina instrument (4000 or equivalent) according to manufacturer's instructions. The samples were sequenced using a 2x150bp Paired End (PE) configuration. Image analysis and base calling were conducted by the Control software. Raw sequence data (.bcl files) generated from Illumina HiSeq was converted into fastq files and de-multiplexed using Illumina's bcl2fastq 2.20 software. One mismatch was allowed for index sequence identification.

Data analysis

After investigating the quality of the raw data, sequence reads were trimmed to remove possible adapter sequences and nucleotides with poor quality using Trimmomatic v.0.36. The trimmed reads were mapped to the Homo sapiens reference genome available on ENSEMBL using the STAR aligner v.2.5.2b. BAM files were generated as a result of this step. Unique gene hit counts were calculated by using feature Counts from the Subread package v.1.5.2. Only unique reads that fell within exon regions were counted. Differential gene expression between *ASCL1* KO and sgGFP was calculated using DESeq2 (10). Differentially expressed genes (DEGs) were selected based on the cut-off criterion (adjusted P value < 0.05 and |log2 (fold change)| > 0.58). Additionally, the protein-coding genes were pre-ranked based on log2 (fold change), and Gene Set Enrichment Analysis (GSEA) was performed using the pre-ranked gene list with 10,000 permutations (14). The Molecular Signatures Database (MSigDB) hallmark gene set and a GO Biological Process was used for the analysis (15).

Immunoblot

Whole cell lysates were extracted from organoids or cells using RIPA (Sigma-Aldrich) buffer supplemented with protease inhibitor cocktail (Roche) and 1x phosphatase inhibitor (ThermoFisher Scientific). Protein concentrations were quantified using the DC protein assay kit (Bio-Rad). 50μg

lysates were subjected to Mini-PROTEAN® TGX™ Gel (Bio-Rad) and then transferred to nitrocellulose membranes (Bio-Rad). Membranes were blocked using 5% Blotting-Grade Blocker (Bio-Rad) and subsequently incubated with primary antibodies overnight at 4℃. Primary antibodies are listed in Supplementary Table 7. The secondary antibodies conjugated with horseradish Peroxidase-conjugated (HRP)(Bio-Rad) were used to detect primary antibody conjugates. Immune complexes were then visualized using Immobilon Classico western HRP substrate (Millipore).

Immunohistochemistry

FFPE slides were prepared as described in the *Histology* section. Antigen retrieval was performed in pre-boiled 10mM pH6 sodium citrate buffer (Sigma-Aldrich) in a microwave and then blocked by 3% hydrogen peroxide for 10 min at room temperature. VECTASTAIN® Elite ABC-HRP Kit (Vector Laboratories) was used to proceed the staining per manufacturer's protocol and developed using a DAB Substrate Kit (Vector Laboratories #SK-4100) followed by hematoxylin counterstaining. Primary antibodies used for immunostaining are listed in Supplemental Table 7. DLL3 staining was performed on the Leica Bond III automated staining platform using the Leica Biosystems Refine Detection Kit. Ventana DLL3 Assay (Roche, 790-7016, clone SP347) was run at the ready to use dilution with EDTA antigen retrieval. Slides were imaged by the NIS-elements imaging system (Nikon) fitted to a Nikon ECLIPSE Ti2 microscope.

Multiplex Immunofluorescence.

After blocking with serum (Vector Lab #S-1000), FFPE slides were incubated with synaptophysin antibody overnight. The next day, Alexa Fluor™ 488 Tyramide Superboost Kits was used to detect synaptophysin antibody conjugates per the manufacturer's protocol (ThermoFisher Scientific). Slides were boiled in 10mM sodium citrate buffer and incubated with KRT8 antibody overnight. Alexa Fluor™ 594 Tyramide Superboost Kits were applied to detect KRT8 conjugates. Following this, slides were boiled in 10mM sodium citrate buffer and incubated with INSM1 antibody overnight. Alexa Fluor™ 647

Tyramide Superboost Kits were then used to capture INSM1 conjugates. Lastly, slides were stained with NucBlue DAPI (ThermoFisher Scientific) and mounted with VECTASHIELD. Vibrance™ Antifade Mounting Medium (Vector Lab # H-1700-10). Slides were imaged in a week by the NIS-elements imaging system (Nikon) fitted to a Nikon ECLIPSE Ti2 microscope. FIJI/Image J software (version 2.1.0/.53c) was used to process and re-color individual fluorescent signals.

<u>Supplemental Tables</u> (corresponding to attached data)

Supplemental Table 1: WTA_DSP_WCM154-CMV-fNICD2

Supplemental Table 2: DEGs_Tumor_TKO-Nicd1_vs_TKO

Supplemental Table 3: DEGs_Tumor_TKO-Nicd1_TrPl_vs_TKO_TrPl.

Supplemental Table 4: GSEA.Results_Tumor_TKO-Nicd1_vs_TKO

Supplemental Table 5: GSEA.Results_Tumor_TKO-Nicd1_TrPl_vs_TKO_TrPl

Supplemental Table 6: sgRNA sequences

Supplemental Table 7: The list of antibodies used in this study

References

1. Lin D, et al. High fidelity patient-derived xenografts for accelerating prostate cancer discovery and drug development. *Cancer Res.* 2014;74(4):1272-83.
2. Labrecque MP, et al. Molecular profiling stratifies diverse phenotypes of treatment-refractory metastatic castration-resistant prostate cancer. *J Clin Invest.* 2019;129(10):4492-505.
3. Beltran H, et al. Divergent clonal evolution of castration-resistant neuroendocrine prostate cancer. *Nat Med.* 2016;22(3):298-305.
4. Beltran H, et al. Molecular characterization of neuroendocrine prostate cancer and identification of new drug targets. *Cancer Discov.* 2011;1(6):487-95.
5. Butler A, et al. Integrating single-cell transcriptomic data across different conditions, technologies, and species. *Nat Biotechnol.* 2018;36(5):411-20.
6. Wolock SL, et al. Scrublet: Computational Identification of Cell Doublets in Single-Cell Transcriptomic Data. *Cell Syst.* 2019;8(4):281-91 e9.
7. Andreatta M, and Carmona SJ. UCell: Robust and scalable single-cell gene signature scoring. *Comput Struct Biotechnol J.* 2021;19:3796-8.
8. van Dijk D, et al. Recovering Gene Interactions from Single-Cell Data Using Data Diffusion. *Cell.* 2018;174(3):716-29 e27.
9. Aran D, et al. Reference-based analysis of lung single-cell sequencing reveals a transitional profibrotic macrophage. *Nat Immunol.* 2019;20(2):163-72.
10. Love MI, et al. Moderated estimation of fold change and dispersion for RNA-seq data with DESeq2. *Genome Biol.* 2014;15(12):550.
11. Reimand J, et al. Pathway enrichment analysis and visualization of omics data using g:Profiler, GSEA, Cytoscape and EnrichmentMap. *Nat Protoc.* 2019;14(2):482-517.
12. Liberzon A, et al. Molecular signatures database (MSigDB) 3.0. *Bioinformatics.* 2011;27(12):1739-40.
13. Sanjana NE, et al. Improved vectors and genome-wide libraries for CRISPR screening. *Nat Methods.* 2014;11(8):783-4.
14. Subramanian A, et al. Gene set enrichment analysis: a knowledge-based approach for interpreting genome-wide expression profiles. *Proc Natl Acad Sci U S A.* 2005;102(43):15545-50.
15. Liberzon A, et al. The Molecular Signatures Database (MSigDB) hallmark gene set collection. *Cell Syst.* 2015;1(6):417-25.
