# Supplementary material for: Notch signaling suppresses neuroendocrine differentiation and alters the immune microenvironment in advanced prostate cancer
Source: J Clin Invest. 2024 Jul 18;134(17):e175217. doi: 10.1172/JCI175217 (PMC11364388; doi:10.1172/JCI175217)

# Fig3C

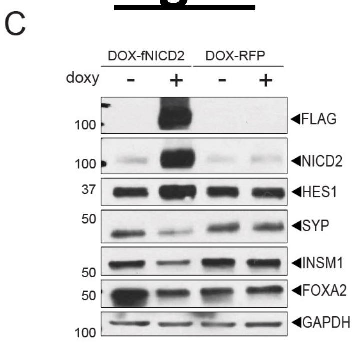


Figure on manuscript

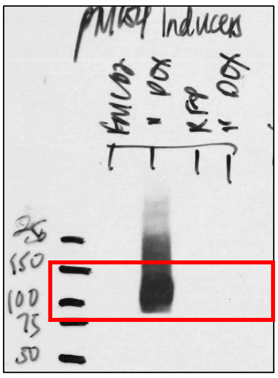

FLAG

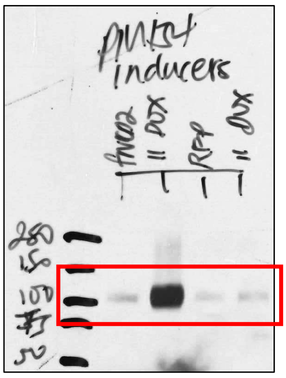

NICD2

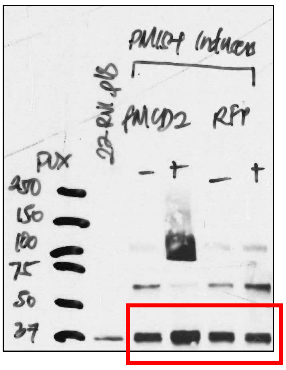

HES1

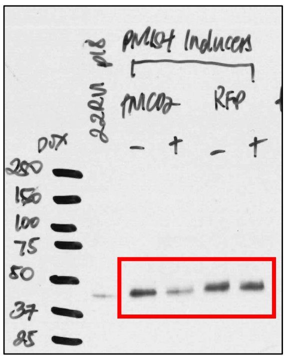

SYP

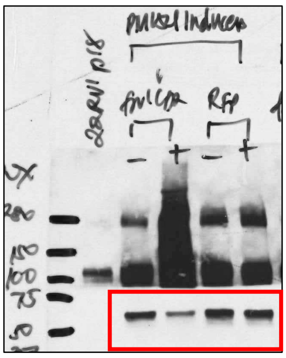

INSM1

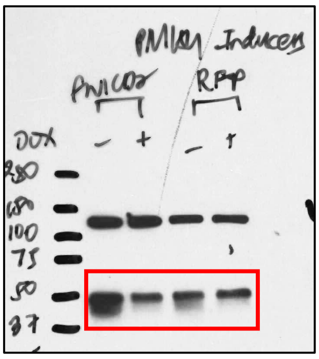

FOXA2

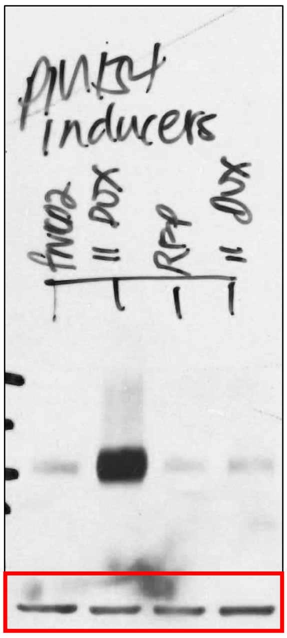

GAPDH

# Supplementary Fig 8

A

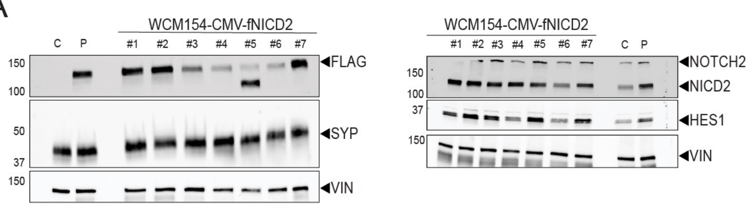


Figure on manuscript

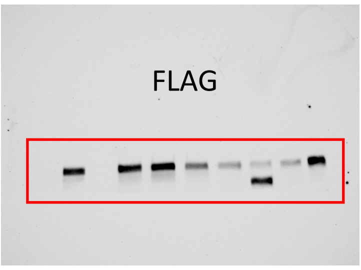


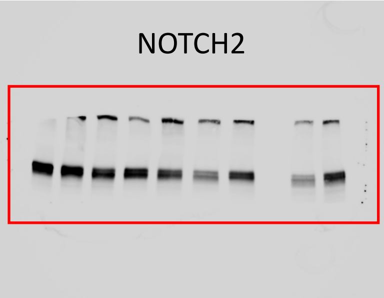


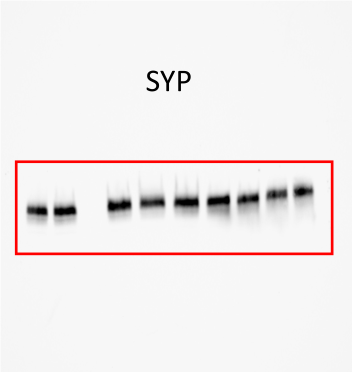


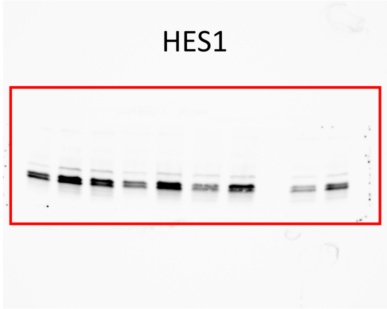


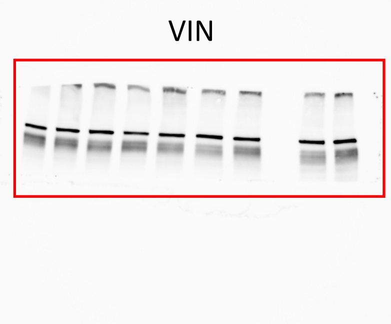


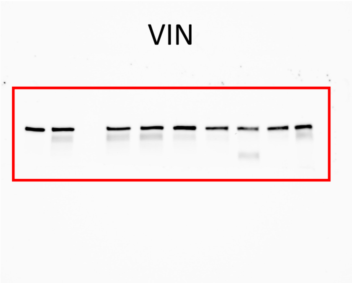

# Supplementary Fig 8E

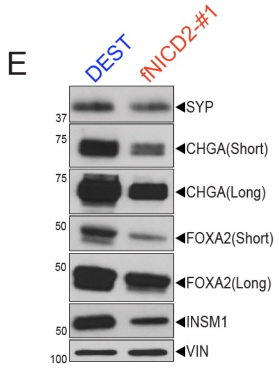


Figure on manuscript

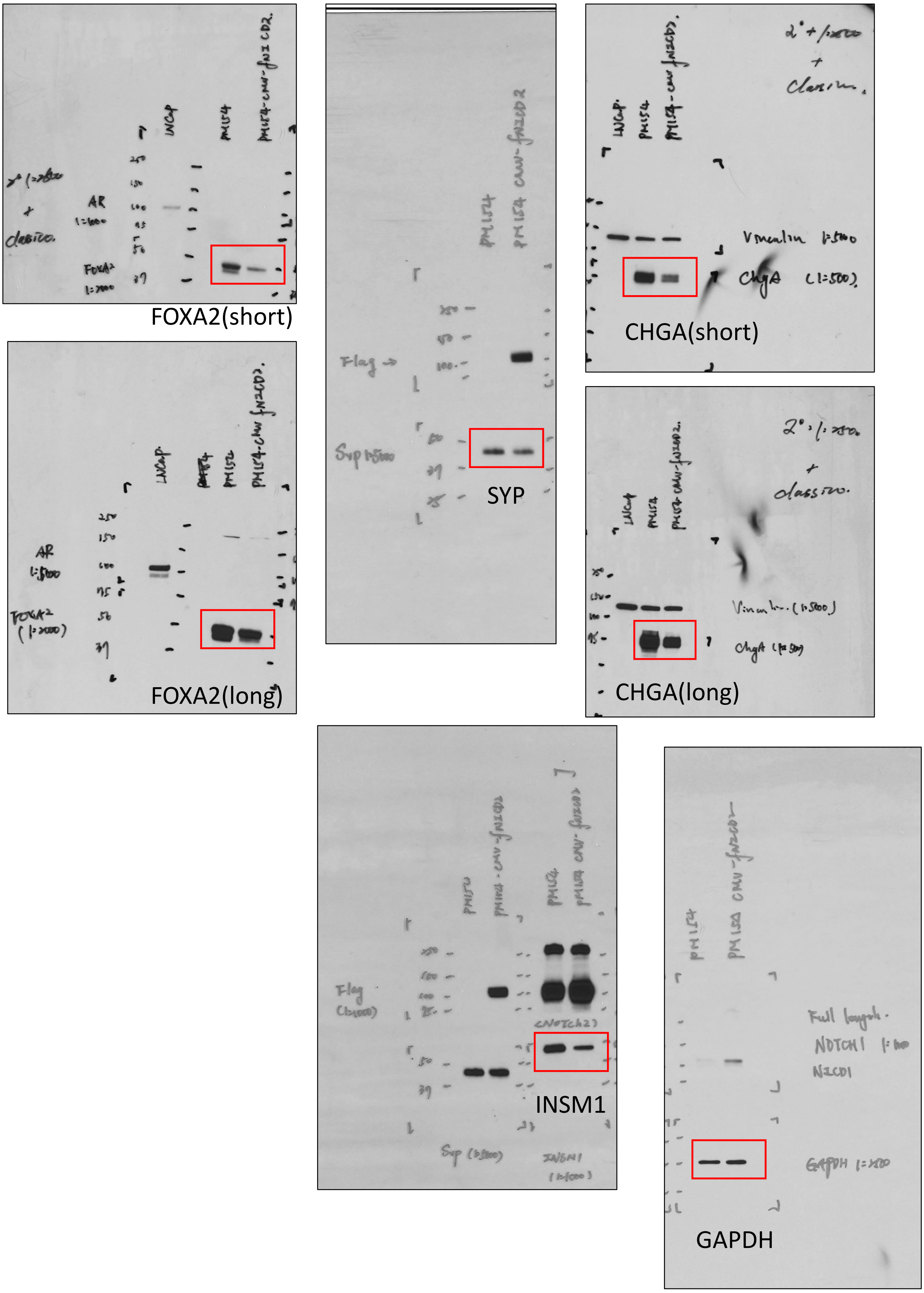

# Supplementary Fig 8I

H

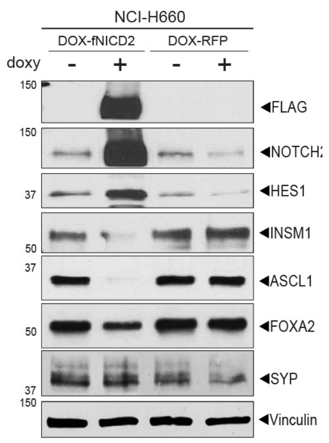


Figure on manuscript

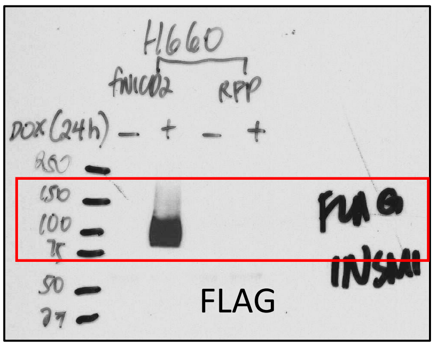


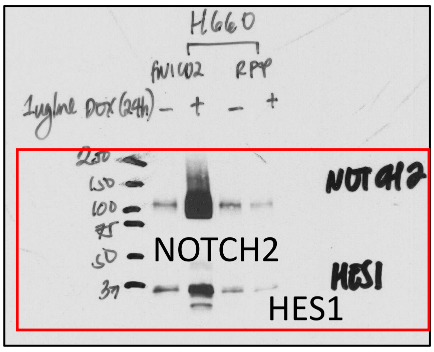


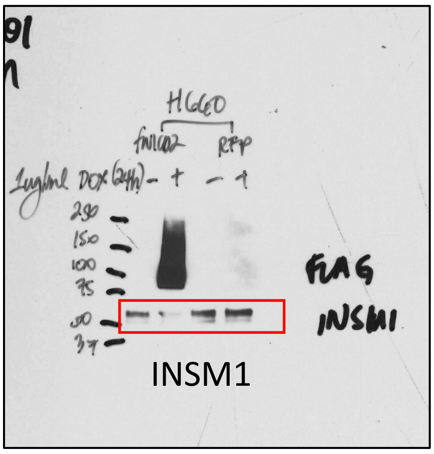


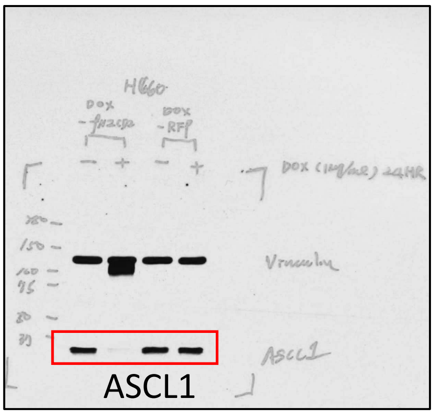


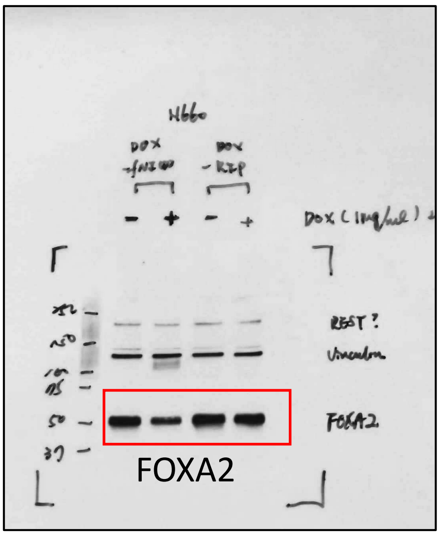


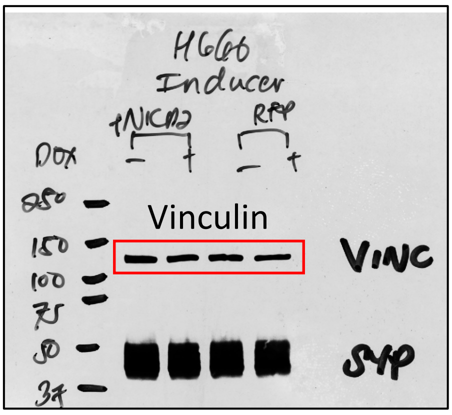

# Supplementary Fig 8J

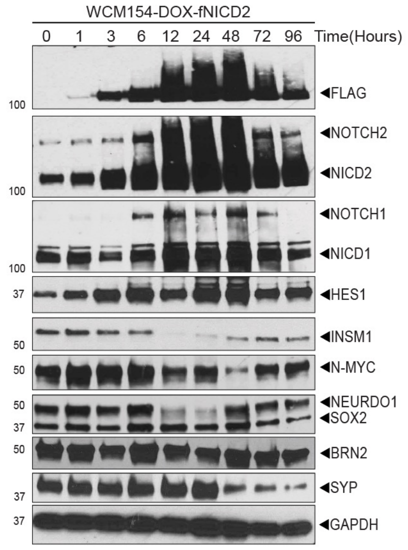


Figure on manuscript

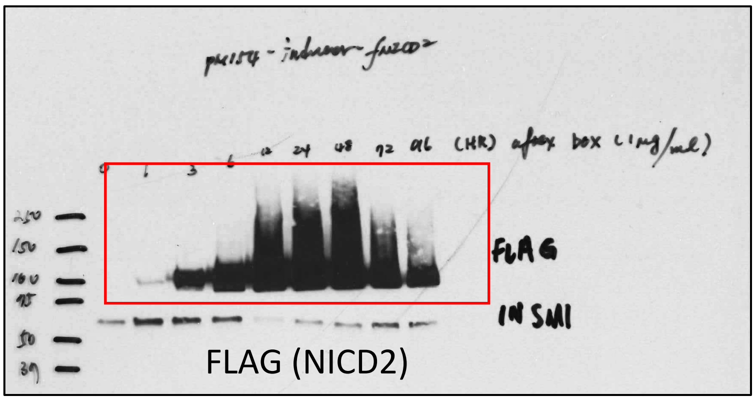


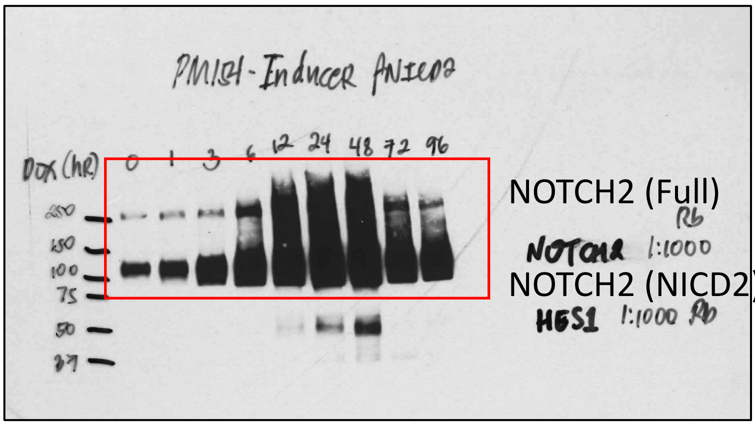


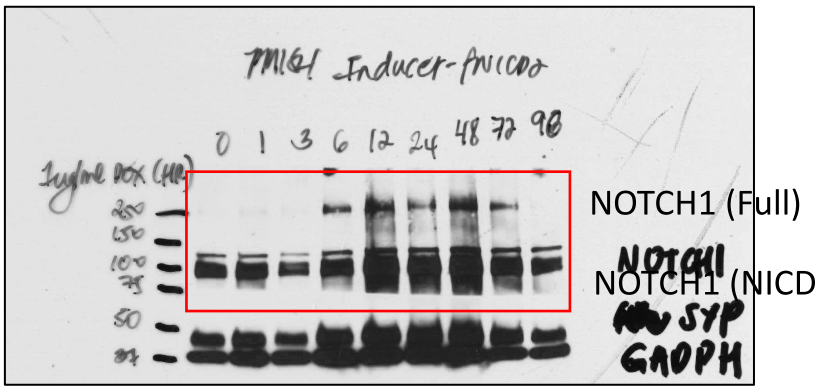


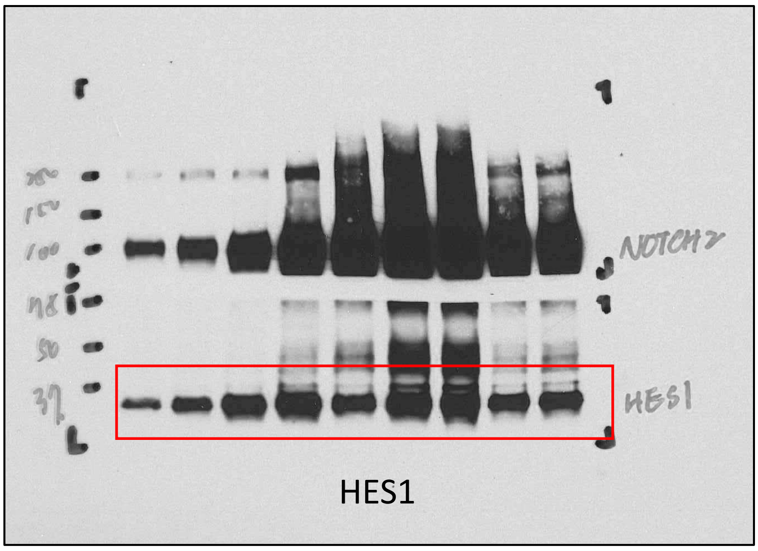


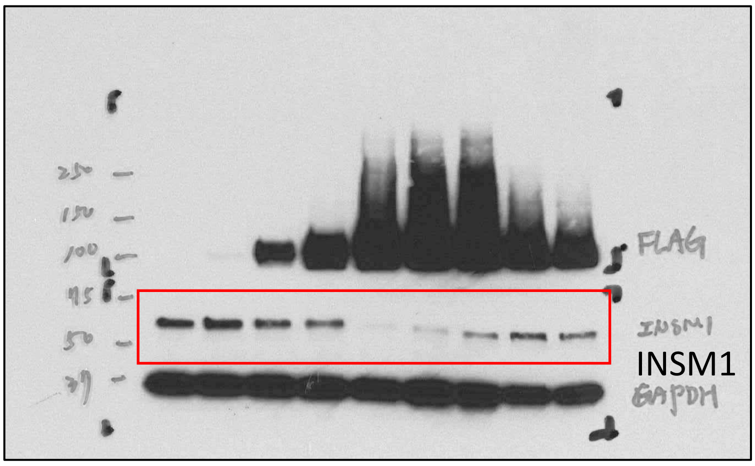

# Supplementary Fig 8J

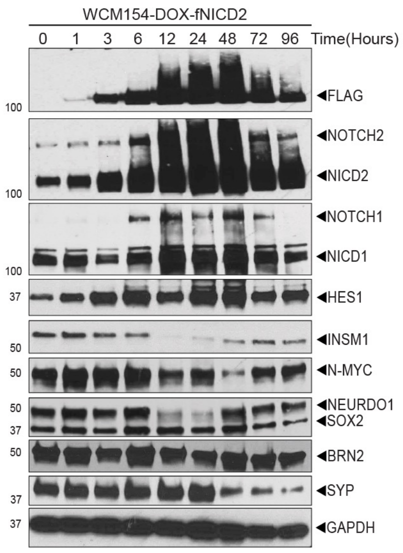


Figure on manuscript

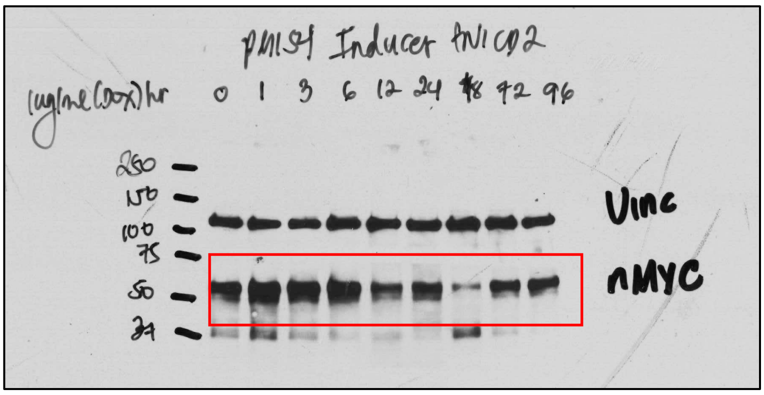


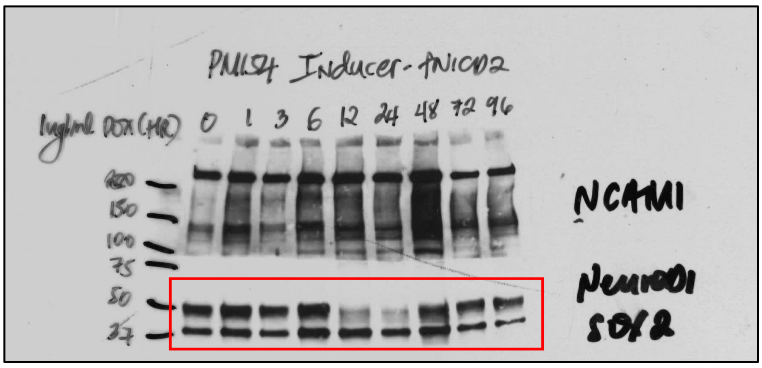


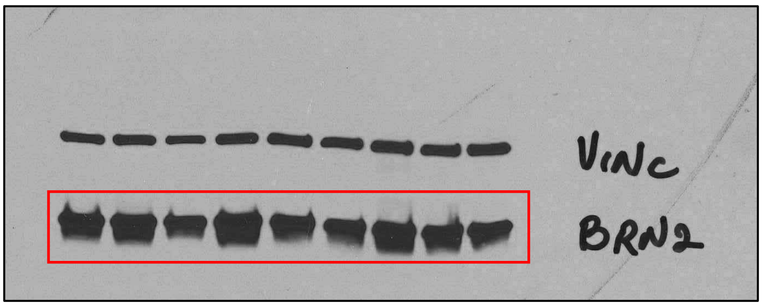


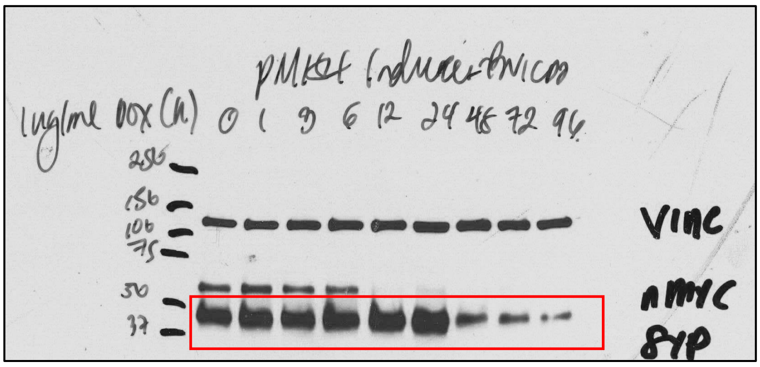


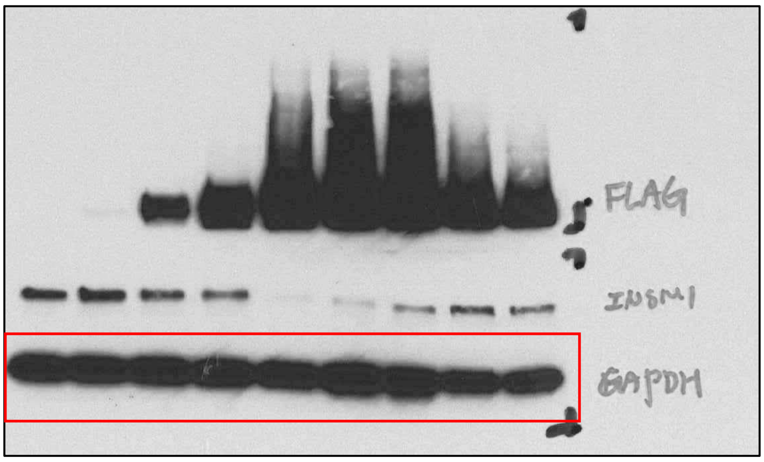

# Fig 6C

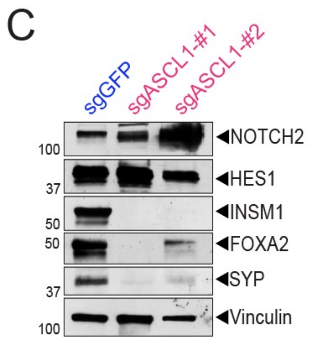


Figure on manuscript

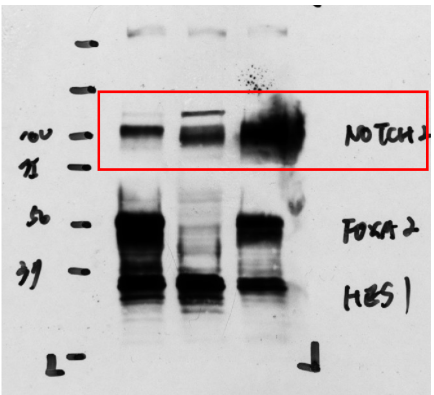


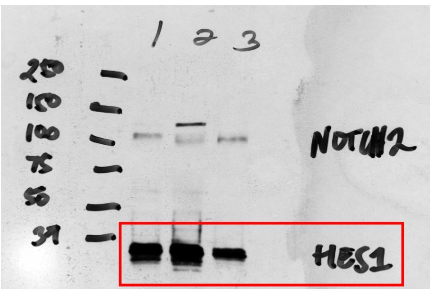


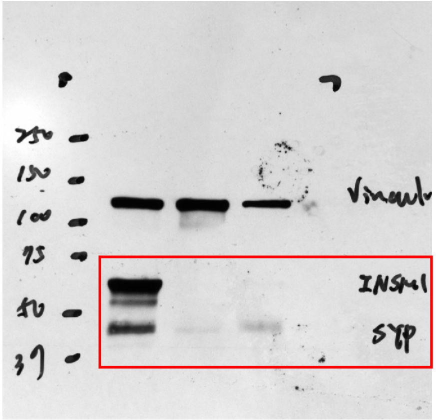


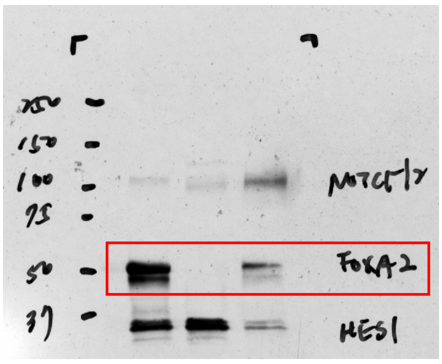


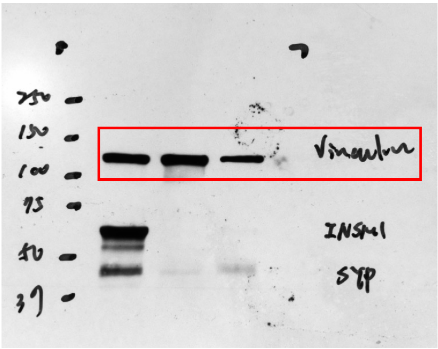

# Supplementary Fig12C

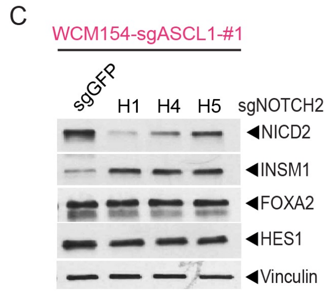


Figure on manuscript

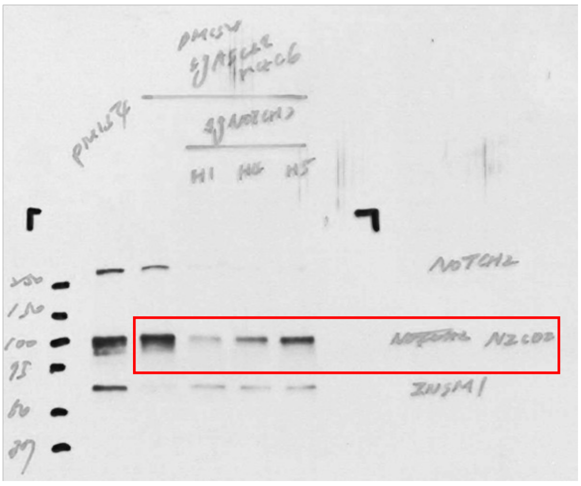

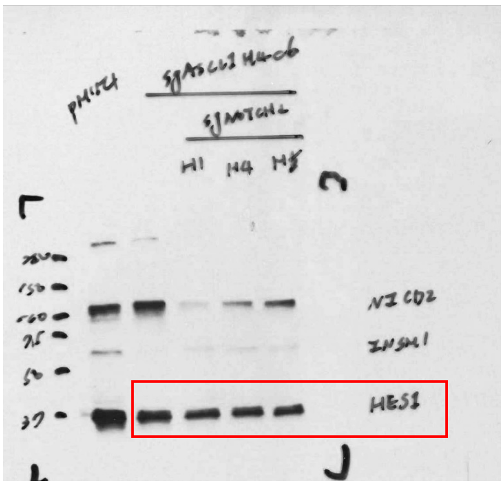

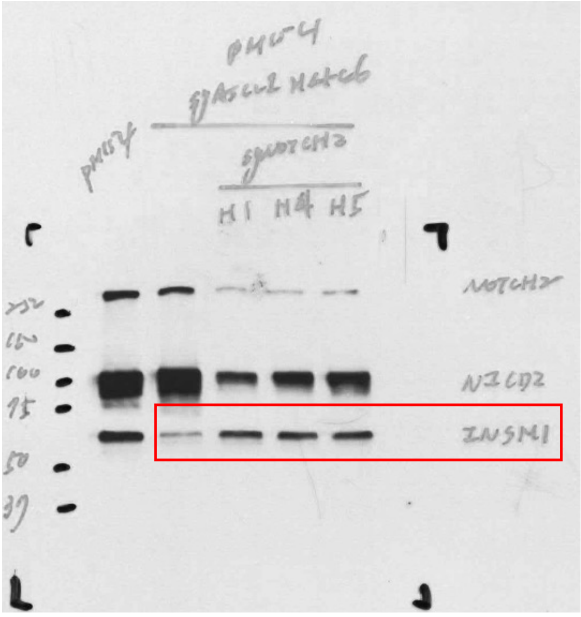

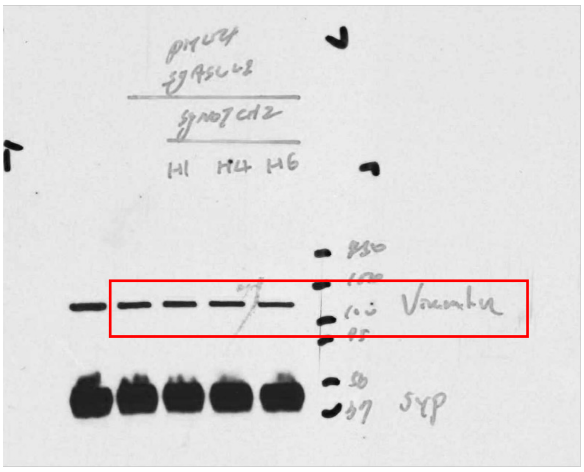

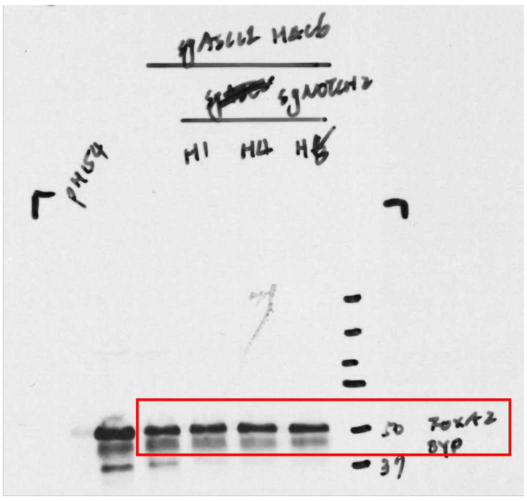

# Fig7

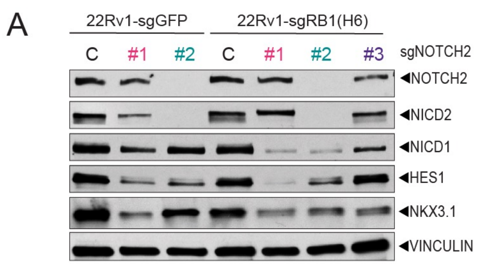


Figure on manuscript

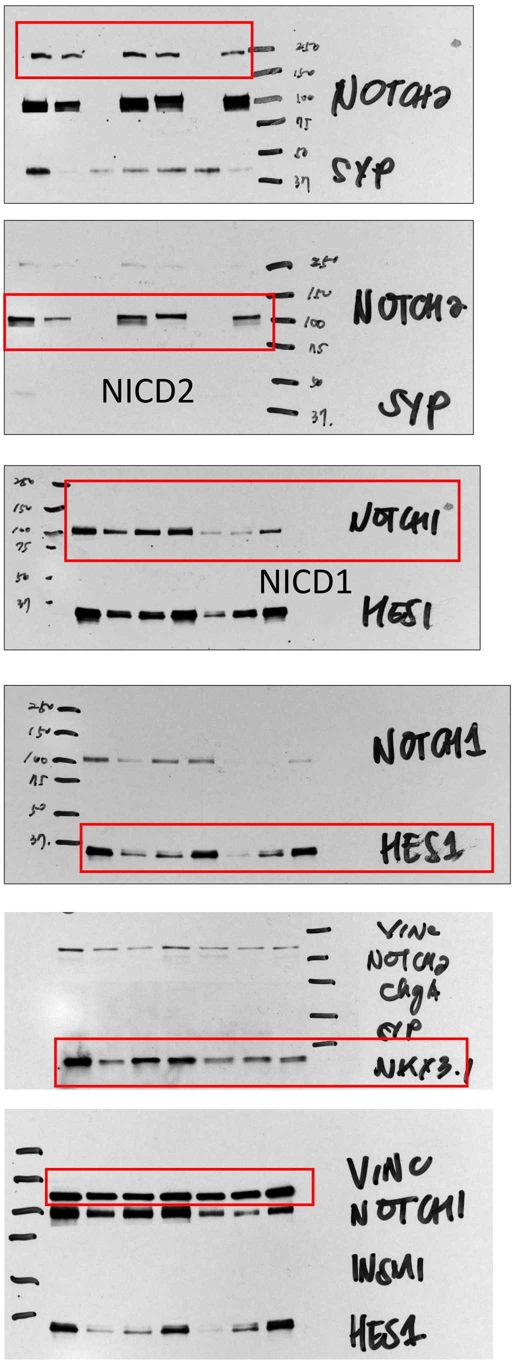

# Supplementary Fig13A

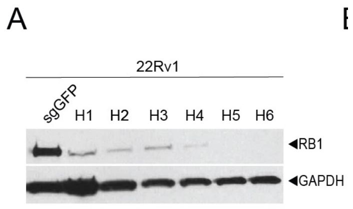


Figure on manuscript

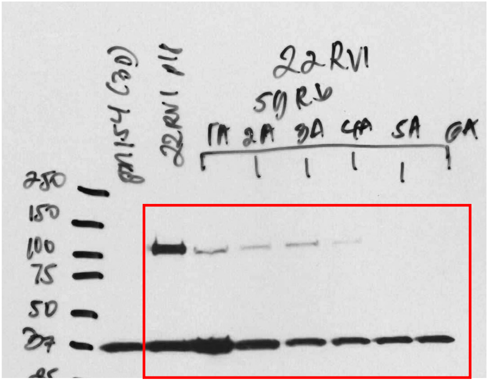

# Supplementary Fig13B

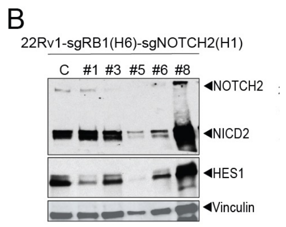


Figure on manuscript

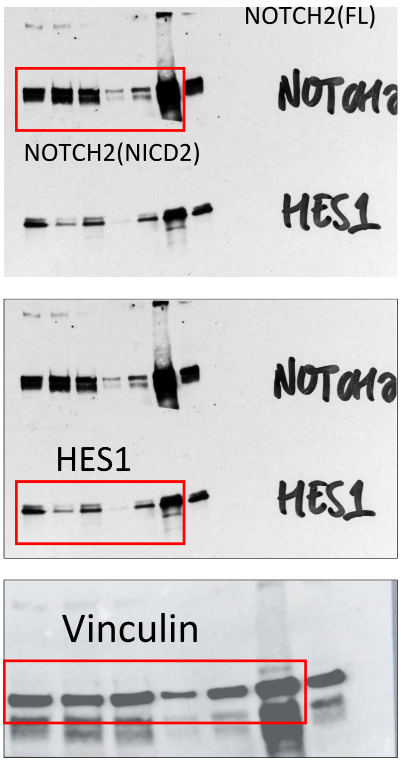

# Supplementary Fig13B

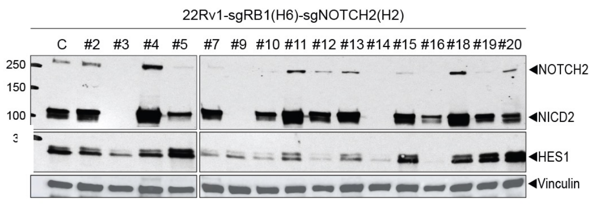


Figure on manuscript

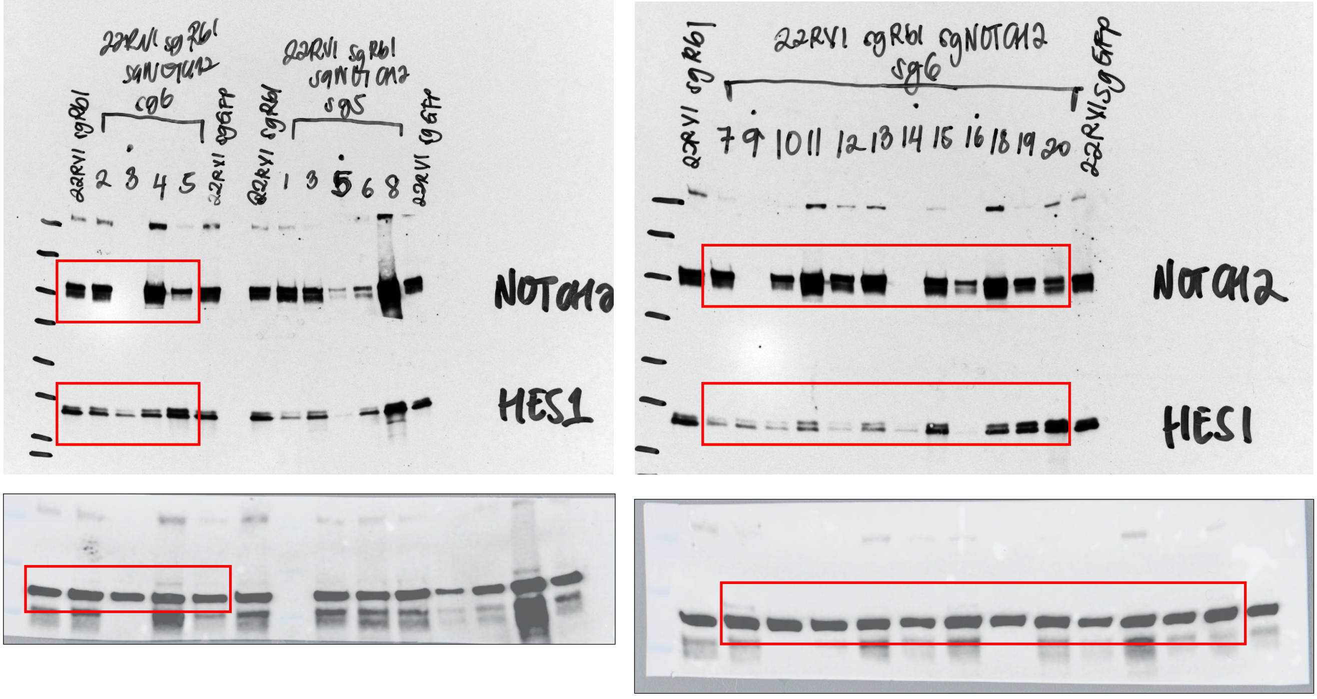

# Supplementary Fig13D

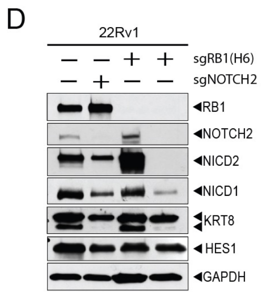


Figure on manuscript

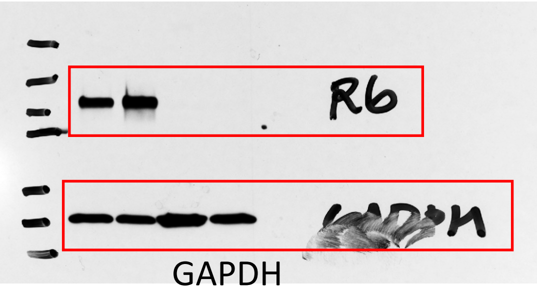


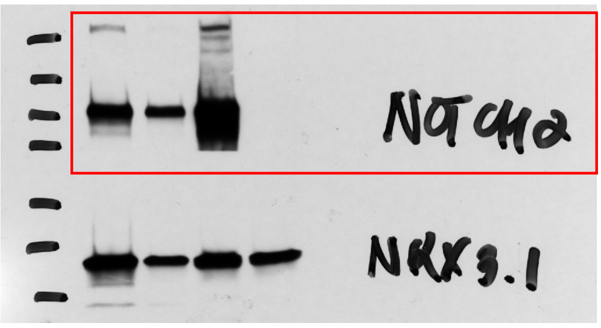


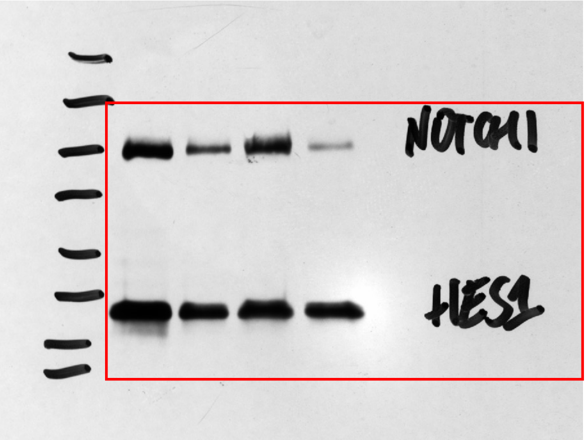


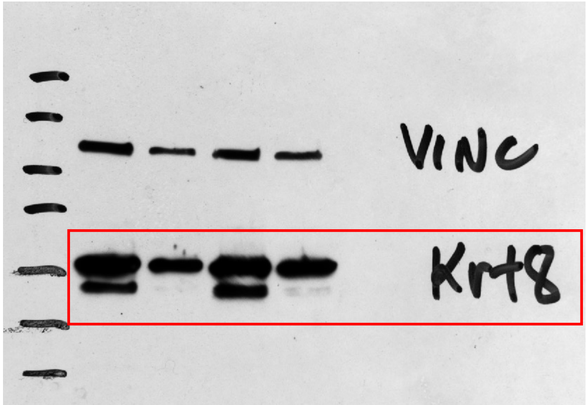

# Supplementary Fig13E

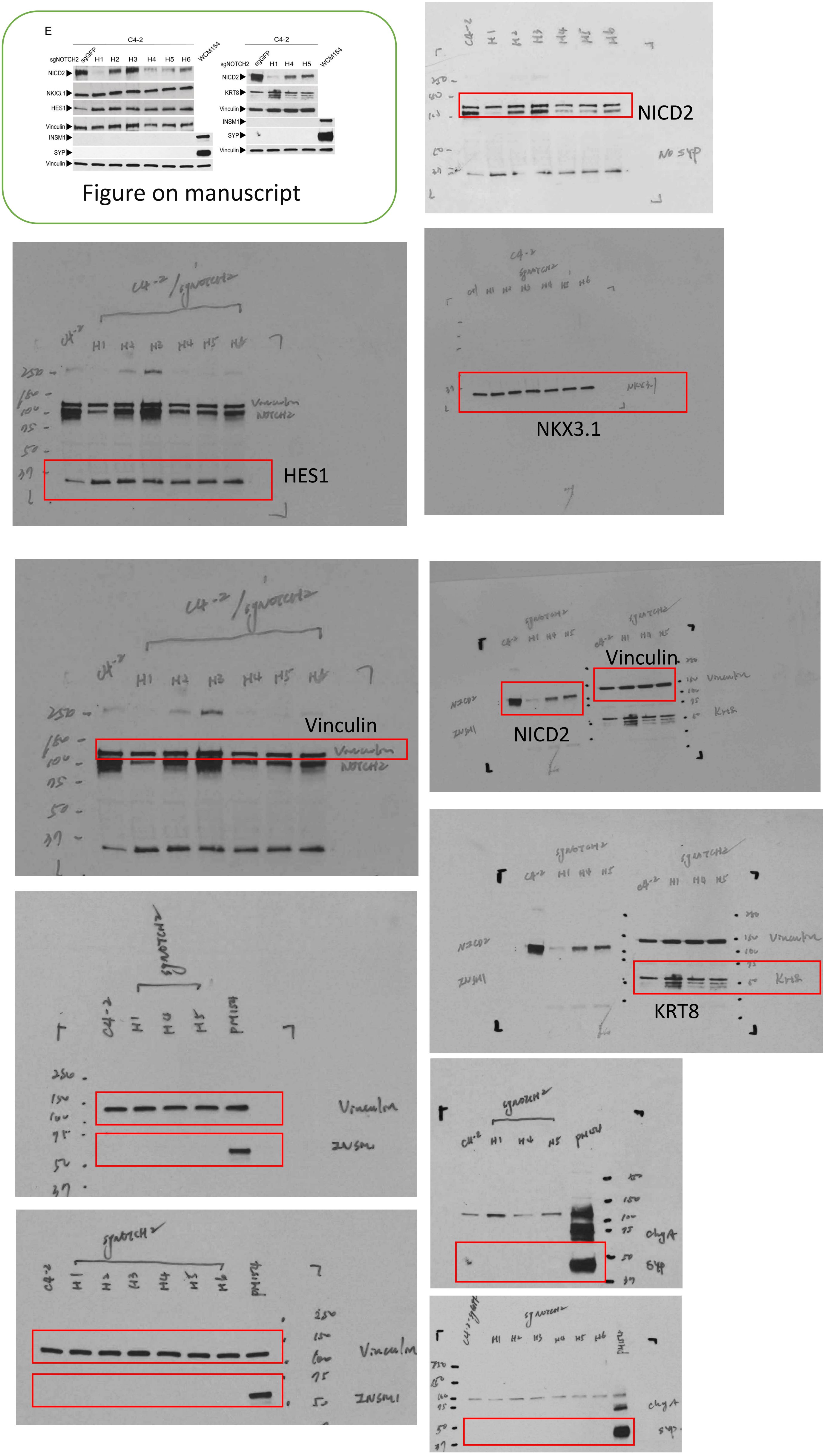

# Supplementary Fig13F

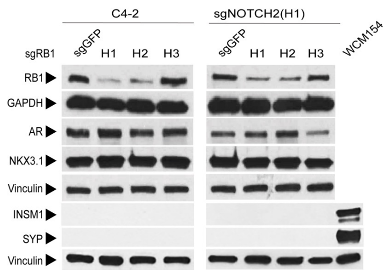

Figure on manuscript

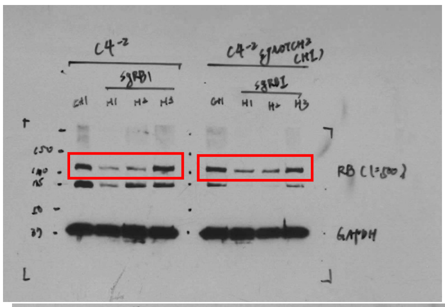


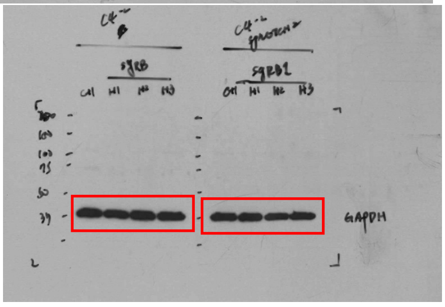


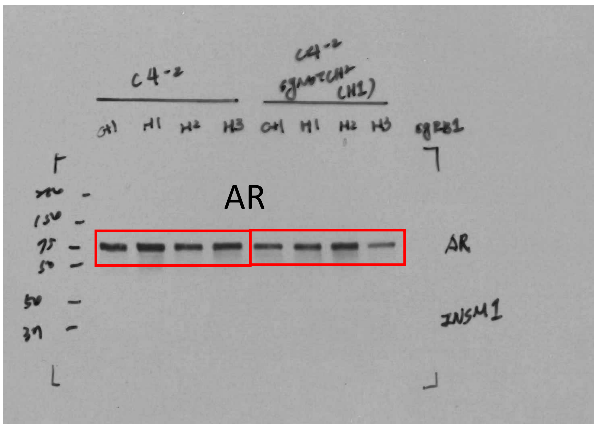


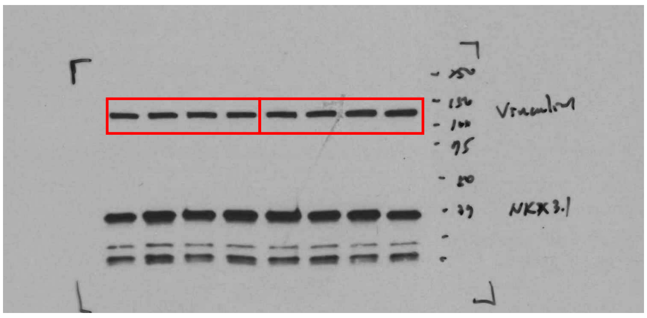


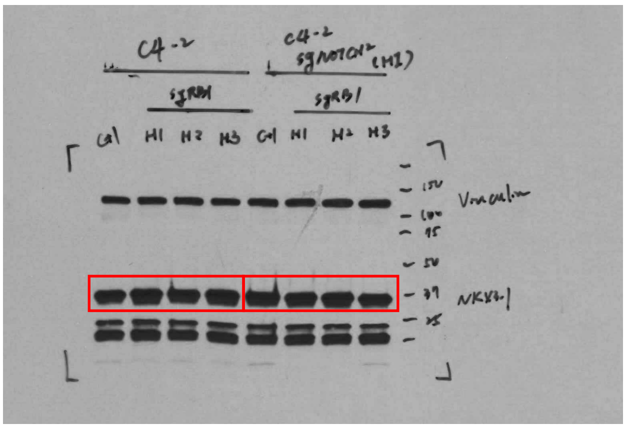


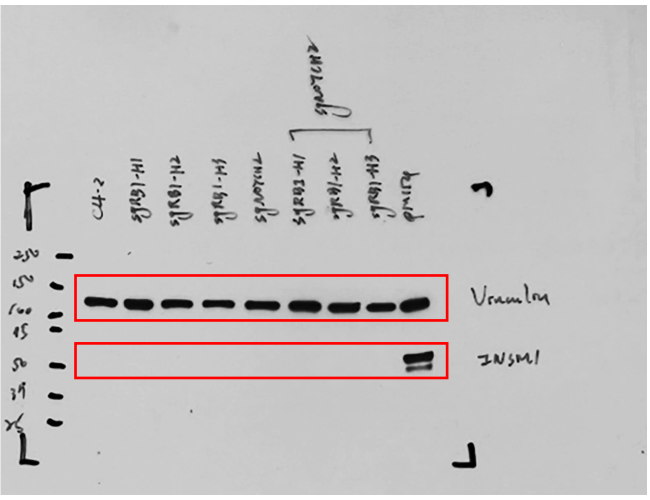


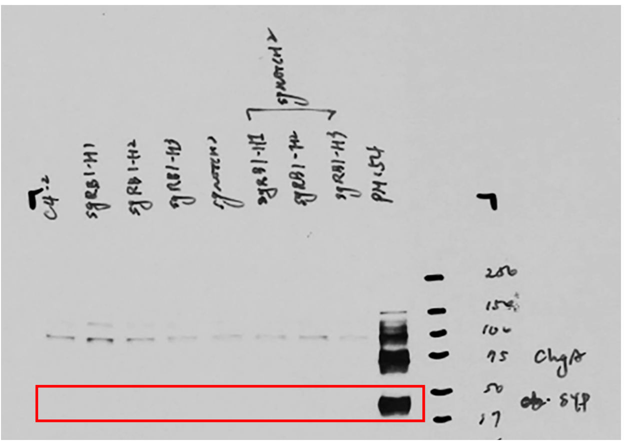

Supplement: Unedited blot and gel images [file jci-134-175217-s227.pdf]
